# Designing a Climate Change Resilient Landscape Connectivity Network From a Multi‐Species Perspective

**DOI:** 10.1002/ece3.71956

**Published:** 2025-09-18

**Authors:** Carlos P. E. Bedson, Ben L. Payne, Chris Sutherland, Danielle J. Greaves, Heather E. White, Fraser Buchanan, Humphrey Q. P. Crick

**Affiliations:** ^1^ Natural England Cambridge UK; ^2^ Department of Natural Sciences Manchester Metropolitan University Manchester UK; ^3^ School of Mathematics University of St Andrews, Mathematical Institute St Andrews UK; ^4^ Defra London UK

**Keywords:** Circuitscape, climate change, conservation, landscape connectivity, protected areas, species

## Abstract

There is strong evidence that climate change causes species range shifts and declines. Protected areas and suitable habitats are important for maintaining biodiversity. Species range changes depend on landscape connectivity between areas, facilitating movement and colonisation. Conservationists should identify landscape connectivity, as climate change causes species to move at different points in time. We quantified national connectivity for England for a sample of nationally important taxa associated with limestone and upland habitats, reflecting the White Peak as example focal region. We generated England‐wide species distribution models for 15 species for three climate change time scenarios (Shared Socioeconomic Pathway 245): current, 2050 and 2090. We inverted these models, applying circuit theory analysis, to create connectivity maps. We applied *z*‐score standardisation to compare differences between scenarios. We considered the top decile of connectivity occurring across the time periods as the ‘landscape connectivity network’. We compared this with the National Character Area framework of land parcels, the Site of Special Scientific Interest (SSSI) map, and quantified landcover in the network. The landscape connectivity network showed future species requirements becoming more diffuse, i.e., the landscape becoming more permeable. High connectivity value land lay in South West or South East England, and the central Pennines; implying range shifts to diverging latitudes. The network measured 1,029,000 ha, with 13% inside SSSIs. In the White Peak focal example, there were 7600 ha, with 38% inside SSSIs. Across England, the network's landcover included broadleaved woodland (365,000 ha), calcareous grassland (55,000 ha), and improved grassland (305,000 ha), the latter thought to be of low biodiversity value. This research innovates by combining connectivity assessments for widely different taxa associated with one habitat type for three climate change time scenarios. It shows how connectivity tends to be concentrated in certain areas of England, thereby identifying important national and regional connectivity areas to support species conservation planning.

## Graphical abstract

We assessed connectivity requirements for 15 species associated with upland and limestone habitats in England and identified climate change mediated shifts: currently showing concentrations of connectivity flows in the far south of England and the northern Pennines; and with the landscape becoming more permeable by 2050 and on to 2090. Of the highest connectivity value land, 13% lay within the protected area SSSI network. Measuring the area of high connectivity value land by landcover type, there were 305,000 hectares of improved grassland, thought to be of low ecological value. This research can help inform land management planning for species conservation.

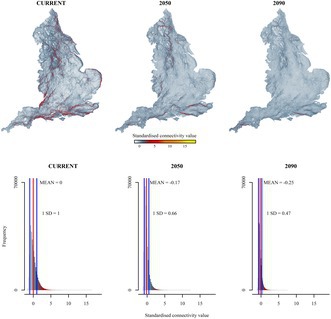

## 1 Introduction

Climate change and anthropogenic sprawl have constrained areas of habitat suitable for many wild animal species, resulting in their decline or demise (Hilty et al. 2020). Climate change contributes to the extinction risk of over 10,000 species worldwide (IUCN 2019; IPCC 2022) and causes range shifts of many species polewards and to higher elevations (Wilson et al. 2005; Hickling et al. 2006; Chen et al. 2011). In Britain, climate change has contributed, at least in part, to the decline of 753 terrestrial and freshwater species by 19% since 1970, with substantial risk forecast for birds, amphibians, and mammals (Burns et al. 2023). In northern Britain, some cold‐adapted species are declining, whilst southern species shift their ranges northwards (Hickling et al. 2006).

Species persistence is also affected by agricultural intensification, land use change, and habitat fragmentation (Fox et al. 2014). Examples include intensive farming, which has been correlated with reductions of animal populations, such as arthropods and birds (Benton et al. 2009); coniferous forest monoculture plantations with low structural complexity, providing low biodiversity (Najera and Simonetti 2010); forests in England being patchy, i.e., one third of their area being less than 75 m from the nearest open edges, not supporting the same levels of ecological soil fauna activity as forest cores (Riutta et al. 2016); peatland ecosystems where birds may be negatively associated with proximity to forest plantations (Wilson et al. 2014); and urbanisation processes generally reduce habitat available for wildlife (Mainwaring et al. 2024). The effects of climate and land‐use change may also be additive, shown by studies of bird, butterfly, moth, and plant distributions in the UK over the past 75 years (Suggitt et al. 2023). Microclimates may moderate these patterns, although they are unlikely to wholly shelter species from climate warming (Suggitt et al. 2012).

The term ‘landscape connectivity’ was originally defined as ‘the degree to which the landscape facilitates or impedes movement among resource patches’ (Taylor et al. 2006), a concept now widely applied in conservation science (Riordan‐Short et al. 2023). Connectivity areas may comprise habitats which provide ecological resources, food and shelter, and facilitate wildlife movements (Taylor et al. 2006). They may also encompass impoverished habitats if these do not impede movement. Connectivity areas support animal migration and dispersal patterns (Keeley et al. 2016). Therefore understanding movement connectivity is important, and especially so when constricted by human activity, compromising metapopulation structures of vulnerable species (e.g., Dickson et al. 2013; Proctor et al. 2015). We have to identify where species move and respond to climate change and fragmented landscapes, to consider protections for these connectivity areas or support the establishment, restoration or management of suitable habitats (Critchlow et al. 2022; Payne and Bro‐Jorgensen 2020). Although there are many studies of climate change mediated species distribution shifts (e.g., Hedrick et al. 2023), few such show *connectivity* shifts (Costanza and Terando 2019).

If landscape connectivity is supported with protections or suitable habitat, this can maintain and improve biodiversity, create resilience, and increase the likelihood of species persistence under climate change (Crick et al. 2020; Hodgson et al. 2022). Moreover, reducing habitat fragmentation and increasing habitat patch size can support bigger populations of some species (Lawton et al. 2010; Crick et al. 2020), improving their resilience to environmental shocks and pressures (Verboom et al. 2010; Oliver et al. 2013, 2015; Newson et al. 2014).

In many countries, protected areas were conceived to conserve important habitats, enabling the persistence of flora and fauna. When prescriptions of protected areas were determined, they often considered discrete ecological features rather than wider ecosystems that facilitate population processes (Cunningham et al. 2021). Studies of landscape connectivity reveal different spatial requirements than those of historically planned protected area networks (Dickson 2016). Some protected areas were contemplated before climate change effects were perceived and are now thus impacted (Araújo et al. 2011; Johnston et al. 2013).

The UK possesses a substantial portfolio of protected areas for nature: including National Nature Reserves and Sites of Special Scientific Interest (SSSIs), chosen to represent important habitats and species at a site level, rather than an ecological network of mutually reinforcing sites through functional connectivity (Gaston et al. 2006; Lawton et al. 2010; Cunningham et al. 2021). These protected areas host a high number of range‐expanding and colonising species (Thomas et al. 2012). Landscapes with relatively high coverage of protected areas support more species than other areas, and whilst species declines occur in all areas, protected landscapes show slower declines in species loss (Cunningham et al. 2021). Separately, contemporary conservation planning in England also references National Character Area profiles (NCA's). This 1990's cartography project considered environmental land forms, habitats and land uses, segmenting England into 159 areas, articulating natural and human land requirements (DEFRA 2024; Natural England 2014a, 2014b, 2024a, 2024b; Swanwick 2002). NCAs have been partly used to inform the designation of protected sites across the UK.

There is increasing momentum towards improving connectivity in the UK. This was proposed in the Lawton et al. (2010) review for the UK Government, ‘Making Space for Nature’, as: ‘An ecological network comprises a suite of high quality wildlife sites, and associated surrounding habitats, which collectively contain the diversity and area of habitat that are needed to support species and which have ecological connections between them that enable species, or at least their genes, to move’. The UK government's 25 Year Environment plan (DEFRA 2018, 2023) set targets for creating or restoring 5000 km^2^ of wildlife‐rich habitat outside the protected site portfolio, and to develop a Nature Recovery Network, complementing and connecting the best wildlife sites. The Protected Site Strategies of the Environment Act (2021) places core protected sites at the heart of Nature Recovery Networks, highlighting the importance of these sites in establishing increased nature connectivity.

We consider designing landscape connectivity as: identifying linkage areas promoting species persistence, considering animal distributions, movements, environmental enablers, and constraints. Contemporary methods may examine species distribution models (SDMs) and circuit theory assessments (Zeller et al. 2012). Correlative SDMs often inform conservation planning, where detailed ecological monitoring mechanisms are lacking (Elith and Leathwick 2009; Araújo et al. 2019). Finding statistical relationships between species occurrence and environmental factors enables prediction and projection to current or new areas or time periods or distribution shifts following climate change (Sillero et al. 2021). The projections of species occurrence probability values are used to derive resistance surfaces, which symbolise the propensity of an animal to occur at each and every location, providing data for connectivity analysis (Zeller et al. 2012; Araújo et al. 2019).

Circuit theory is used to generate ecologically informed spatially explicit landscape‐scale connectivity surfaces. It works by replicating a random walk of a species, modelling all possible pathways between patches, represented as a grid of nodes from which simulated electrical current flows, measured in amperes, through a landscape represented by the resistance surface (Shah and McRae 2008; McRae et al. 2013; Spear et al. 2015, 129; Marrotte and Bowman 2017). The resistance surface may comprise a rich combination of species‐associated data: environmental characteristics, habitat associations, anthropogenic influences, and *sensu* SDM probability of occurrence (Zeller et al. 2012). The continuous surface enables analysis of multiple alternative movement pathways of a species metapopulation without ancillary data e.g., dispersal parameters (Grafius et al. 2017), sub‐populations, breeding rates (Spear et al. 2015). Resultant flows indicate high connectivity value areas: concentrated, channelled, impeded, or constrained; or alternatively, more diffuse, wider pathways.

### 1.1 Research Objectives

We demonstrate an approach for developing management‐relevant projections of connectivity based on predicted future species–habitat relationships under climate change. We apply SDM techniques and circuit theory to identify connectivity opportunities for nationally important species.

This study aims to track species range shifts of connectivity, as we seek understanding of where animals move at different points in time, for enduring conservation planning. We consider the NCA framework and protected area network of England, which may only partly encompass natural connectivity (Travers et al. 2021) and may be revised. The study also considers improved grassland, which receives nutrient enrichment, known to reduce biodiversity (Isbell et al. 2013) and which covers and fragments large parts of England, including potential areas for habitat transformation to help species recovery.

As case study, we considered the White Peak NCA (hereafter ‘White Peak’) of Derbyshire UK (Natural England 2014a). This high elevation grassland plateau hosts a narrow spectrum of habitats and was chosen as a pilot region for implementing a Protected Site Strategy (Environment Act 2021), addressing the vulnerability of its 14 SSSI areas to neighbouring intensive agricultural land use. It is one of the most southerly high elevation landscapes in England and is particularly affected by climate change (Kendon et al. 2024).

Pertaining to this area, and limestone and upland habitats more widely across England, we selected a sample of species: four Lepidoptera; five birds; five mammals; one reptile. We constructed connectivity models for the entirety of all England. We assessed three climate scenarios (Shared Socioeconomic Pathway (SSP) 245; current climate, 2050, 2090 (IPCC 2022)) to understand species responses. We considered highest connectivity value land occurring at two consecutive or all three time points in time as the ‘landscape connectivity network’. We ranked connectivity by NCA, and measured the amount of protected land and landcover.

Our hypotheses were:

H1: Climate change results in substantial shifts in high connectivity value land for species across England.

H2: A large portion of high connectivity value land is designated SSSI.

H3: A large portion of high connectivity value land is improved grassland.

H4: The White Peak has a relatively large proportion of high connectivity value land and within the SSSI network.

H5: Most remaining White Peak high connectivity value land outside SSSIs is improved grassland.

## 2 Materials and Methods

### 2.1 Geography and Study Site

**FIGURE 1:**
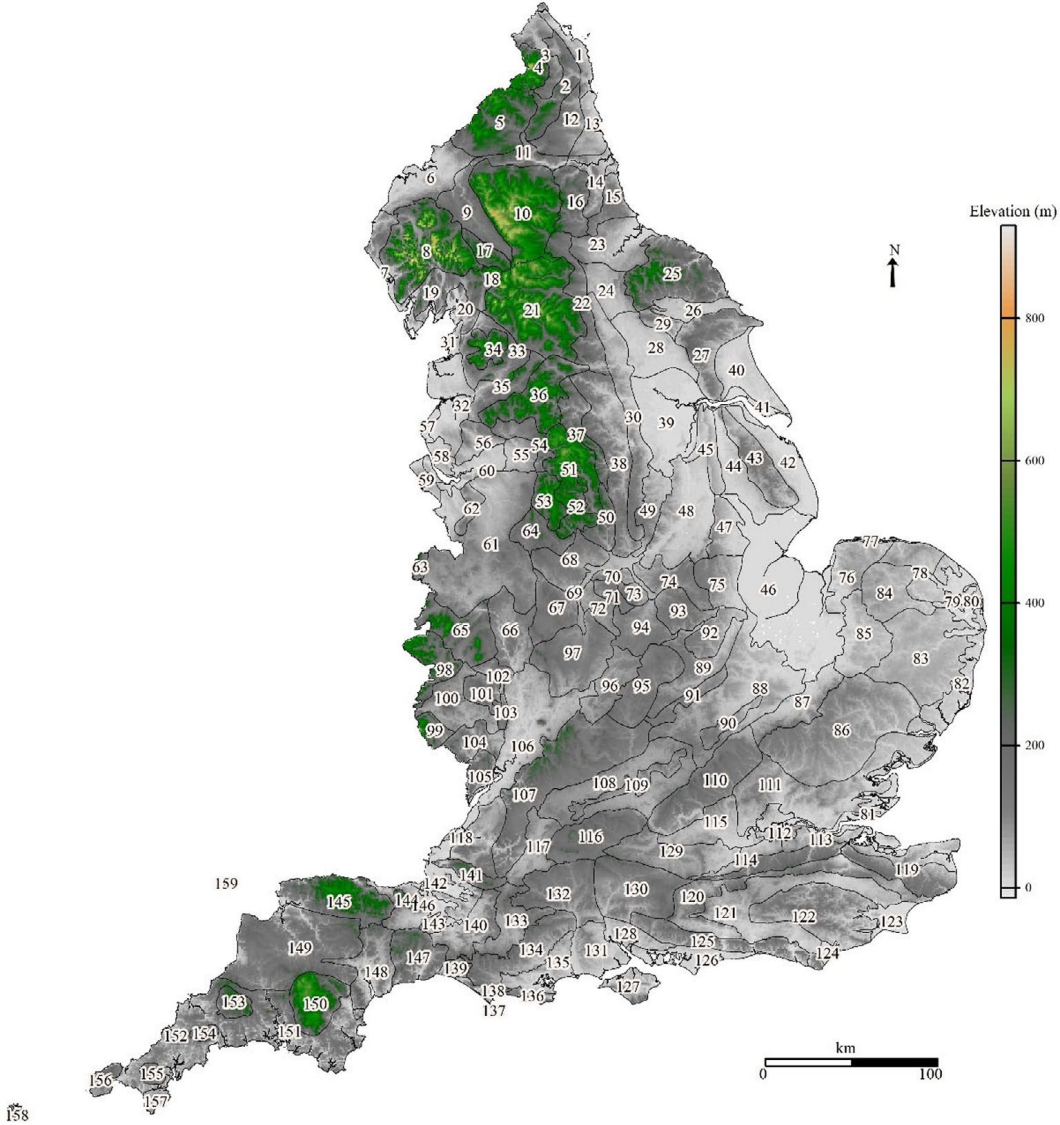
Relief map of England. Numbers indicated NCAs identities (Table S1). Number 52 = White Peak, south end of Pennine mountain range. Shape file sourced from Natural England datasets (https://www.data.gov.uk/dataset/21104eeb‐4a53‐4e41‐8ada‐d2d442e416e0/national‐character‐areas‐england1).

Our country‐wide assesment referenced the framework of regional geographies of England defined by the NCA national mapping project (Figure 1, Table S1). This characterised interactions of natural and human factors, referring to varied data sources: altitude, land form, surface geology, farm types, settlement, woodland and field patterns, archaeology, industry and parkland (Swanwick 2002), assessing data with a polythetic hierarchical classification, TWINSPAN (Hill 1979), producing 159 separate contiguous regions with distinct characteristics (Chris Blandford Associates 1997). This made no reference to climate or species data. The NCA framework has been used to inform human land use and ecological planning and monitoring.

For our case study, we focused on the White Peak (Derbyshire, UK) (Natural England 2004, 2014b, 2024b). This area of the south Pennines (Figure 1) was designated in 2005 under EC Habitats Directive 92/43 on Conservation of Natural Habitats and of Wild Fauna and Flora, as Peak District Dales Special Area of Conservation (English Nature 2005), referencing the rare carboniferous limestone calcareous grassland plateau, incised by rivers and dales, containing diverse habitats: ash woodland, heath, scrub, rocky slopes, alpine screes, and scrub mosaics, hosting important or rare flora and fauna (English Nature 2005), much within 14 separate SSSIs. Over recent decades, intensive dairy farms replaced calcareous grassland, with improved grassland, i.e., *Lolium* spp., providing poor cover and nutrition for wildlife (Riley 2005; Isbell et al. 2013).

### 2.2 Species Data for Landscape Connectivity Modelling

**FIGURE 2:**
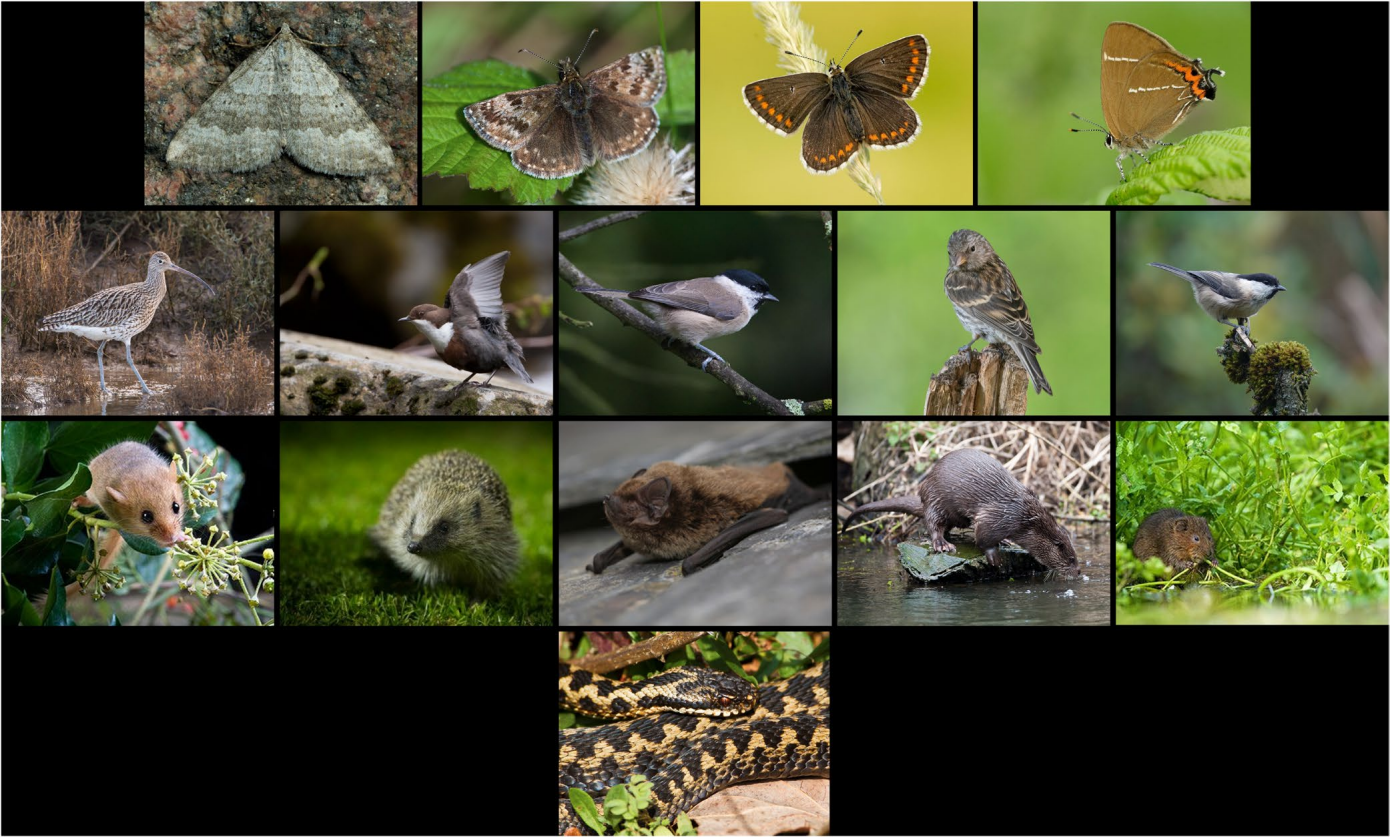
Species sample representative of limestone and upland habitats. Top row: lepidoptera: chalk carpet moth, dingy skipper, northern brown argus, white letter hairstreak. Second row: birds: curlew, dipper, marsh tit, twite, willow tit. Third row: mammal: hazel dormouse, hedgehog, Leislers' bat, otter, water vole. Fourth row: reptile: adder. Photos with thanks (see Acknowledgements).

We collated a list of nationally important species representative of limestone and upland habitats, informed by Natural England's Species Recovery Programme (which incorporates those listed in Section 41 habitats of the Natural Environment and Rural Communities Act 2006) and species listed by the UK Biodiversity Action Plan (1994); or listed on Joint Nature Conservation Committee (JNCC) red lists as amber/red status (JNCC 2023); or classified as threatened or near threatened on the IUCN Red Lists at England, Great Britain, or International Scale. We considered 15 species: some generalist, some specialist; four Lepidoptera, five birds, five mammals, one reptile (Table 1, Figure 2), being overall a sample comprising phylogenetic breadth and diversity of habitat preferences and diets. The selection was not considered an assemblage, i.e., a community of species having strongly specialised ecological interactions (Ovaskainen and Abrego 2021). Each was considered separately from others.

**TABLE 1:** Species for connectivity modelling. Red lists and national trend definitions differ per taxa, according to source. Section 41 priority species identified from Natural Environment and Rural Communities Act 2006 (England). For Lepidoptera, ‘occupancy’ defined as probability of occurrence at 1 km scale 1970–2019; birds ‘population’ means trend vs. relative index 1994–2021; for mammals ‘population’ means extrapolated abundance estimate; for adder ‘population’ means absolute abundance from expert opinion and literature. Habitat preferences sourced from references under ‘Red list status by source’ or ‘National Trend’.

Taxa		Red list status by source	National trend	Section 41 species	Habitat preference
**Lepidoptera**		**British Butterflies (Fox et al**. 2019, 2022 **)**	**Occupancy 2010–2019 (Fox et al**. 2022 **)**		
Chalk carpet moth	*Scotopteryx bipunctaria*	Least concern	NA	Yes	Calcareous grassland
Dingy skipper	*Erynnis tages*	Least concern	−3%	Yes	Calcareous grassland
Northern brown argus	*Aricia Artaxerxes*	Vulnerable	−39%	Yes	Calcareous grassland
White letter hairstreak	*Satyrium w‐Album*	Vulnerable	+35%	Yes	Woodland
**Birds**		**Birds of Conservation Concern (Stanbury et al**. 2021 **)**	**Population 1995–2021 (Harris et al**. 2022 **)**		
Curlew	*Numenius arquata*	Red	−48%	Yes	Grassland
Dipper	*Cinclus cinclus*	Amber	−62%	No	Riparian
Marsh tit	*Poecile palustris*	Red	−46%	Yes	Woodland
Twite	*Linaria flavirostris*	Red	NA	Yes	Grassland
Willow tit	*Poecile montanus*	Red	−90%	Yes	Woodland
**Mammals**		**Mammal Society (Mathews and Harrower** 2020 **)**	**Population 1995–2018 (Mathews and Harrower** 2020 **)**		
Hazel dormouse	*Muscardinus avellanarius*	Vulnerable	−48%	No	Woodland
Hedgehog	*Erinaceus europaeus*	Vulnerable	−39%	Yes	Urban/general
Leisler's bat	*Nyctalus leisleri*	Near threatened	NA	No	Woodland/grassland
Otter	*Lutra lutra*	Least concern	+62%	Yes	Riparian
Water vole	*Arvicola amphibius*	Endangered	−94%	Yes	Riparian
**Reptiles**		**Amphibian and Reptile Conservation (Foster et al**. 2021 **)**	**Population (Foster et al**. 2021 **)**		
Adder	*Vipera berus*	Near threatened	Declining	Yes	Grassland/moorland

We gathered species records from National Biodiversity Network Atlas or taxa‐specific sources (see acknowledgements) for 1980–2020. We considered species identifications as correct. We assumed species had accomplished range filling and were at equilibrium with their environment, occurring in all suitable areas. We noted species such as Eurasian curlew ( *Numenius arquata* ) and twite ( *Linaria flavirostris* ) are migratory, i.e., their full range of environmental predictor values might not be represented.

We sought sufficient species records to achieve at least 10 per environmental predictor layer. In most cases, we gathered records for 2001–2020 with resolution < 1 km (Table 2). For species with exceptionally few records (Chalk carpet moth *Scotopteryx Bipunctaria*, Northern brown argus *Aricia Artaxerxes*, Twite *Linaria flavirostris* and Leisler's bat *Nyctalus leisleri* ), we extended dates to 1980–2000 or lowered resolution to maximise the number of presence points. Species record locations were allocated one per 1 km raster cell present, using the R function [fuzzysim::gridRecords] (Barbosa 2015), i.e., removing duplicates and thinning points.

**TABLE 2:** Species record data selection and ensemble model evaluation results for SDMs. Columns: ‘Years of records’ shows the time period of data selection. ‘Resolution (km)’ shows the scale of data selection from 1 to 1.5 to 10 km. ‘Presences points’ shows the number of records elicited with ‘Pseudo‐absence points’ being ~3× presence points. Model evaluation scores: True Skill Statistic (TSS) > 0.90 = perfect; 0.70–0.90 = very good; 0.50–0.70 = good; Receive operating characteristic (ROC) > 0.90 = excellent; 0.70–0.90 = good (Fielding and Bell 1997).

Species	Years of records	Resolution (km)	Presence points	Pseudo‐absences (PA)	Ensemble weighted mean		Ensemble committee average	
					TSS	ROC	TSS	ROC
**Lepidoptera**								
Chalk carpet moth	2001–2020	1.5	262	786	0.79	0.97	0.82	0.98
Dingy skipper	2001–2020	1.0	913	2739	0.72	0.94	0.67	0.93
Northern brown argus	1981–2020	1.5	328	987	0.91	0.99	0.93	0.99
White letter hairstreak	2001–2020	1.0	532	1596	0.71	0.93	0.67	0.93
**Birds**								
Curlew	2001–2020	1.0	11,955	35,909	0.55	0.86	0.57	0.86
Dipper	2001–2020	1.0	4423	13,267	0.69	0.93	0.80	0.95
Marsh tit	2001–2020	1.0	10,198	30,599	0.61	0.89	0.63	0.89
Twite	2001–2020	10.0	552	1659	0.57	0.87	0.62	0.88
Willow tit	2001–2020	1.0	4129	12,387	0.58	0.88	0.57	0.88
**Mammals**								
Hazel dormouse	2001–2020	1.0	2919	8755	0.70	0.93	0.78	0.95
Hedgehog	2010–2020	1.0	30,281	90,868	0.55	0.86	0.62	0.87
Leisler's bat	1981–2020	1.5	441	1322	0.71	0.93	0.68	0.93
Otter	2001–2020	1.0	4566	13,701	0.56	0.87	0.55	0.88
Water vole	2001–2020	1.0	2267	6801	0.65	0.91	0.65	0.91
**Reptiles**								
Adder	2001–2020	1.0	892	2676	0.72	0.94	0.67	0.94

We generated three pseudo‐absences per presence point with the R function: [fuzzySim:selectAbsences], facilitating prediction through all geographies and environmental characteristics (Barbet‐Massin et al. 2012; Thuiller et al. 2024). To mitigate potential spatial autocorrelation of species responses, data were allocated to geographic blocks, using the R function [blockCV:cv_spatial] (Valavi et al. 2018). This enabled cross‐validation, testing each block and its respective sample of presence and absence of data separately for model validity. We found 40 hexagonal blocks, 1/20th the square root of England area, provided adequate representation of presence/absence data, allocated randomly into five groups, i.e., block folds (advice, M. Barbosa).

### 2.3 Species Distribution Modelling

Current climate temperature and precipitation data (1970–2000) were sourced from WorldClim (Fick and Hijmans 2017) (Table 3). Future climate values were accessed from WorldClim, referencing forecasts of Hadley Centre HadGEM3‐GC31‐LL Global Circulation Model data provision to the Coupled Model Intercomparison Project Phase 6 (CMIP6) (Webb 2019). SDMs were sensitive to future climate forecasts of different Shared Socio‐economic Pathways (SSPs) and time horizons. We modelled with SSP ‘245’ a mid‐range forecast of greenhouse gas emissions with some climate protection measures being taken (IPCC 2022) and two time periods: 2050 and 2090. Data was downloaded at 30 s resolution, with R function [terra::project] projecting to kilometre scale when creating model algorithms, and hectare scale when projecting distributions.

**TABLE 3:** Environmental predictors in SDMs. Those with asterisks were included. Those without asterisks were excluded as colinear. Landcover categories L5, L9, L10, L11 collapsed into one, we assigned as ‘L30 Bog and grass and heather’. Landcover descriptions: Marston et al. (2022).

	WorldClim (Fick and Hijmans 2017)		Landcover (UK CEH) (Marston et al. 2022)
*	BIO1 = Annual Mean Temperature	*	L1 Broadleaved woodland
*	BIO2 = Mean Diurnal Range (Mean of monthly (max temp—min temp))	*	L2 Coniferous woodland
*	BIO3 = Isothermality (BIO2/BIO7) (×100)	*	L3 Arable
*	BIO4 = Temperature Seasonality (standard deviation ×100)	*	L4 Improved grassland
	BIO5 = Max Temperature of Warmest Month		L5 Neutral grassland
*	BIO6 = Min Temperature of Coldest Month	*	L6 Calcareous grassland
	BIO7 = Temperature Annual Range (BIO5‐BIO6)	*	L7 Acid grassland
*	BIO8 = Mean Temperature of Wettest Quarter		L8 Fen, marsh, swamp (Fen, marsh, swamp; saltmarsh)
	BIO9 = Mean Temperature of Driest Quarter		L9 Heather
	BIO10 = Mean Temperature of Warmest Quarter		L10 Heather grassland
	BIO11 = Mean Temperature of Coldest Quarter		L11 Bog
*	BIO12 = Annual Precipitation		L12 Rock (Inland rock; supralittoral rock/sediment; littoral rock/sediment)
	BIO13 = Precipitation of Wettest Month		L13 Water (Saltwater; freshwater)
	BIO14 = Precipitation of Driest Month		L20 Urban (Urban; suburban)
*	BIO15 = Precipitation Seasonality (Coefficient of Variation)	*	L 30 Bog, grass and heather = L5 + L9 + L10 + L11
	BIO16 = Precipitation of Wettest Quarter		Rivers, roads, topography (Ordnance Survey 2023)
	BIO17 = Precipitation of Driest Quarter		
	BIO18 = Precipitation of Warmest Quarter		
	BIO19 = Precipitation of Coldest Quarter		

Landcover data representing shelter, food resource, enabler or impediment to animal movement, was sourced from UK Centre for Hydrology and Ecology (UK CEH) Landcover Map at 10 m resolution (Marston et al. 2022). We used dominant types comprising > 1% of England area, collapsing most remaining into one category (Table 3). We transformed landcover data to continuous ‘distance in metres to feature’ layers, using R functions [terra::segregate] and [terra::gridDist] (distance to feature, eight directions): aggregated to kilometre resolution for modelling, hectare for distribution projections.

Rivers and waterways represent water sources, movement pathways or barriers to animals. We used Ordnance Survey (2023) Master Map Water Network vector data. Roads represent movement paths or barriers to animals; road density implies human infrastructure, either deterrent or shelter for some species. We used Ordnance Survey (2023) Open Roads vector data. Both were converted to binary presence/absence rasters, hectare resolution, with R function [terra::rasterize], calculating ‘distance in metres’ to ‘rivers’ or ‘roads’ as continuous data layers, using R function [terra:: gridDist], aggregating to kilometre resolution for model creation, hectare for projections.

For topography we sourced Ordnance Survey (2023) Lidar Digital Terrain Model 50 m resolution, transforming to topographic position index (tpi) (De Reu et al. 2013). Data was resampled to hectare resolution, applying R function [spatialEco::tpi] with a 250 m focal window, aggregated to kilometre resolution for model creation, hectare for distribution projections.

All environmental variables were assessed for multi‐collinearity; those with Pearson correlation > 0.70 were excluded, retaining the one we considered to most likely act as an ecological predictor (Guisan et al. 2017, 104–108). SDM model creation referenced variables at kilometre resolution. Distribution projections used variables reprojected at hectare resolution. For maps of environmental variables, see Supporting Information.

Preparation of England‐extent SDMs followed correlative methods (Guisan et al. 2017, chapter 19). We used a range of models with different capabilities or accuracies, reported by error matrix metrics (Allouche et al. 2006): General Linear Model (GLM) using Ordinary Least Squares regression may provide lower accuracy yet is more generalizable; Flexible Discriminant Analysis (FDA) fits piecewise functions accommodating non‐linear responses with strong discriminatory capability; Random Forest (RF) fits several recursive partition trees to different samples of data, providing high accuracy, though with possible overfitting; MAXENT assesses a point‐presence probability distribution encompassing all pixels i.e., background information, applying a penalised maximum likelihood model to trade off model fit and complexity. For details, see Guisan et al. (2017) chapters 10–13.

We made five model runs for each block fold: GLM, quadratic, zero interactions, selection by AIC, family = binomial (link = ‘logit’); FDA, method = ‘mars’; RF, number of trees = 500, node size = 5; MAXENT, quadratic and hinge features. We used 80/20 data split for model calibration and evaluation as per the block folds; the results were assessed using true skill statistic (TSS) and receiver operating characteristic (ROC) metrics, independent of prevalence (Allouche et al. 2006). Models scoring > 0.70 ROC were retained.

We produced ensemble models from GLMs, FDAs, RFs, and MAXENT and subsequently evaluated accuracy with TSS and ROC. We compared weighted‐mean and committee averaged (summarised agreements) ensemble models on TSS and ROC values. We found that the committee‐averaged model values were overall marginally higher than weighted‐mean models; hence, we used these for projections (Table 2). We produced graphs showing variable importance and species responses for the committee‐averaged ensemble model (Thuiller et al. 2024) (Supporting Information).

We projected probability of occurrence maps, referencing ensemble models for current climate, 2050, and 2090 scenarios, with climate predictors per time period; and including distances to rivers, roads, landcover types, and tpi which we assumed the same throughout. we assumed the same throughout. We produced a multi‐variate environmental similarity surface (MESS) map showing how many predictor variables were invoked for future climate scenarios (Elith et al. 2010).

### 2.4 Connectivity Modelling

We used the SDM projections of probability of occurrence (current, 2050 and 2090 scenarios) to create resistance surfaces (Zeller et al. 2012). We considered eight negative exponential values (−0.25, −0.5, −1, −2, −4, −8, −16, −32) to tailor resistance surfaces dependent on species inclination to move away from highly suitable habitat (Keeley et al. 2017). No genetic or telemetry data was available to inform movement propensities. Instead, we consulted the literature (Supporting Information) and expert opinion to determine plausible exponential values. We considered 10 species unlikely to leave suitable habitat: i.e., strong resistance, exp. = −0.25. We regarded curlew, dipper, and twite, though volant, as preferring to alight within suitable habitat, exp. = −2 implying medium resistance. We regarded hedgehog, Leisler's bat, and otter as having low resistance to moving from suitable habitat, exp. = −4.

We modelled connectivity assessing each species' resistance layer in Circuitscape via Julia (Hall et al. 2021). Connectivity analysis concerned England (area = ~13,000,000 ha) and modelled at the hectare scale. We considered species free ranging and created omnidirectional flows with 25 nodes, making 300 pairwise connections (Koen et al. 2014; Brennan et al. 2020). To minimise the influence of node proximities on connectivity flows, we added a 123 km buffer (i.e., 20% Koen et al. 2014), total resolution = ~73 m pixels. To avoid undue overestimation of resistance, the buffer was filled with random values, with the same proportion of values as the resistance raster (Koen et al. 2010). To further eliminate node effects (where England's shape results in slightly higher current density in the South West), we made a resistance layer with all values at 1, which was divided into each species model, i.e., a null model to normalise them (Brennan et al. 2020).

We sought to identify how the spatial distribution of connectivity would change, comparing equally between climate change scenarios. We stacked (summed) the resulting species connectivity models, creating ‘all species’ connectivity maps for current, 2050, and 2090 scenarios (Calabrese et al. 2014; Brennan et al. 2020). To compare contemporary connectivity to future projected connectivity, we first scaled the current connectivity surface using a *z*‐score standardisation (Borcard et al. 2018, 32):

$$x_{i}^{\prime }=\left(x_{i}-{\bar{x}}_{i}\right)/\sigma_{x}$$

where $x_{i}$ is the connectivity value of pixel i=1,…,N, $\bar{x}$ is the landscape average connectivity, and $\sigma_{x}$ is the standard deviation of the connectivity surface. In order to directly compare surfaces, we scaled the 2050 values ($y_{i}$) and 2090 values ($z_{i}$) using the contemporary mean and standard deviation:

$$y_{i}^{\prime }=\left(y_{i}-{\bar{x}}_{i}\right)/\sigma_{x}$$

$$z_{i}^{\prime }=\left(z_{i}-{\bar{x}}_{i}\right)/\sigma_{x}$$

This ‘relative standardisation’ allows for the convenient interpretation of the 2050 and 2090 being y or z standard deviations different from the contemporary average. To understand differences between connectivity scenarios we produced frequency distribution histograms, and summary statistics using R function [pastecs::stat.desc] and calculated Pearson correlation between the scenarios using R function [terra::Layercor].

To select priority connectivity areas, Circuitscape output maps are typically represented with thresholds e.g., the top percentiles (Dickson 2016) or deciles (McRae et al. 2016). Thus, to determine the landscape connectivity network we selected top decile connectivity locations, enabling relative comparison of the same amount of space across the three time periods, providing transparency to the positions of climate mediated connectivity shifts. We determined the landscape connectivity network by considering top decile areas present in all time periods (i.e., climate analogues) as ‘permanent connectivity’, and those present in current/2050 and 2050/2090 as ‘stepping stones’ (regardless of contiguity to permanent connectivity), similar to principles of Wilson et al. (2009). These combined to represent the design of the ‘landscape connectivity network’. Top decile areas occurring only in single time periods current or 2050 or 2090, or both current and 2090 were considered impermanent and excluded. We ranked NCAs by area of landscape connectivity proportionate to NCA area (Dickson et al. 2013). We quantified how much of the network was within SSSIs, and the amount of land cover in the network, per NCA, including the White Peak.

### 2.5 Analytical Environment

All GIS data preparation and visualisation were conducted with ArcGIS (ESRI USA) and R Statistical Software (v.4.4.2; R Core Team 2025) using packages including ‘ecospat’ (Broennimann et al. 2023), ‘fuzzySim’ (Barbosa 2015), ‘sf’ (Pebesma and Bivand, 2023), ‘spatialeco’ (Evans 2023), ‘stars’ (Pebesma and Bivand, 2023), and ‘terra’ (Hijmans et al. 2023). Analysis referred to England scale shape (GB National Outlines shapefile, Ordnance Survey 2023), British National Grid (BNG) origin *x* = 85720, *y* = 7020. White Peak was origin *x* = 404020, *y* = 430320.

Both SDMs and Circuitscape required large amounts of computer processing, with long lead times for testing models. The utilitarian R package biomod2 is single‐threaded: analysing models with 10 k/30 k presence/absence points, projecting over three time periods, on a 128GB RAM computer with Ryzen 97950X3D 16‐Core 4.2GHz processor, took 6–10 h run time. Circuitscape runs (44 m pixels) with 4 parallel cores took up to 7 h. The eventual production of all 45 Circuitscape models (15 species, 3 climate scenarios) was computed by the Defra Data Analytics and Science Hub, employing Azure Databricks Single node cluster, Standard_E48ads_v5 (AMD EPYC 7763v (Milan) [x86‐54]), 384gb Memory and 48 cores, with Databricks runtime 16.3 and Julia v1.1.11.5 Circuitscape v.5.14.0 installed; 3 clusters with clean‐ups took 17 h.

## 3 Results

### 3.1 Species Models

SDMs achieved high or credible test scores (Table 2). Models for species with relatively small distributions (e.g., northern brown argus) showed higher scores. Lowest scores were for curlew and hedgehog, which had the highest number of presence points.

MESS predictions showed mostly seven or eight climate variables predicting for projecting species distributions, reducing in some areas to four in 2050 and two in 2090 (Supporting Information).

### 3.2 SDM Environmental Model Predictions

Species' responses to environmental variables showed patterns broadly consistent with species macro‐ecology (see Supporting Information). Lepidoptera were most attracted to warmer or drier climate values and specific landcover types. Chalk carpet moth showed strong attraction to calcareous grassland; dingy skipper to broadleaved woodland and calcareous grassland; northern brown argus to wetter conditions (BIO 12) and calcareous grassland; white letter hairstreak to less temperature variation (BIO 4) and broadleaved woodland.

Bird species were associated with colder, wetter climate values and specific landcovers. Curlew was associated with lower temperature variation values (BIO 3 and 4) and bog/grass/heather landcover class; dipper had mixed associations with temperature seasonality (BIO 4), a strong association with acid grassland, rather than rivers; marsh tit was strongly associated with broadleaved woodland; twite was strongly associated with colder winter temperatures (BIO 1); and willow tit was strongly associated with lower rainfall seasonality (BIO 15) and broadleaved woodland.

Mammal species were associated with drier climates and general landcovers. Hazel dormouse was associated with drier conditions (BIO 12) and woodland; hedgehog was less influenced by climate, more associated with arable land and roads; Leisler's bat was associated with a mixture of climate values, colder winters (BIO 1), less temperature seasonality (BIO 4), and acid grassland; otter was associated with less temperature seasonality (BIO 4) and rivers; and water vole was associated with lower rainfall (BIO 12) and rivers. Adder showed the strongest preference for drier conditions of BIO 12 (annual precipitation) and mixed landcovers.

### 3.3 Distributions and Connectivity Models

SDMs and connectivity maps showed projections of species, with range shifts from the current scenario, through 2050 and 2090, to very different climatically suitable areas of England. Chalk carpet moth, dingy skipper, and white letter hairstreak would increase connectivity over time. During the current climate, bird species showed distributions in northern England, mostly heading northwards with climate change, though curlew, dipper, and marsh tit also headed to South West England. Mammal species distributions with climate change were projected to be expanding. Adder was projected to expand in the south. The White Peak was important for chalk carpet moth, northern brown argus, white letter hairstreak, dipper, willow tit, hedgehog, and Leisler's bat. See Supporting Information.

### 3.4 National Species Connectivity Flows

**FIGURE 3:**
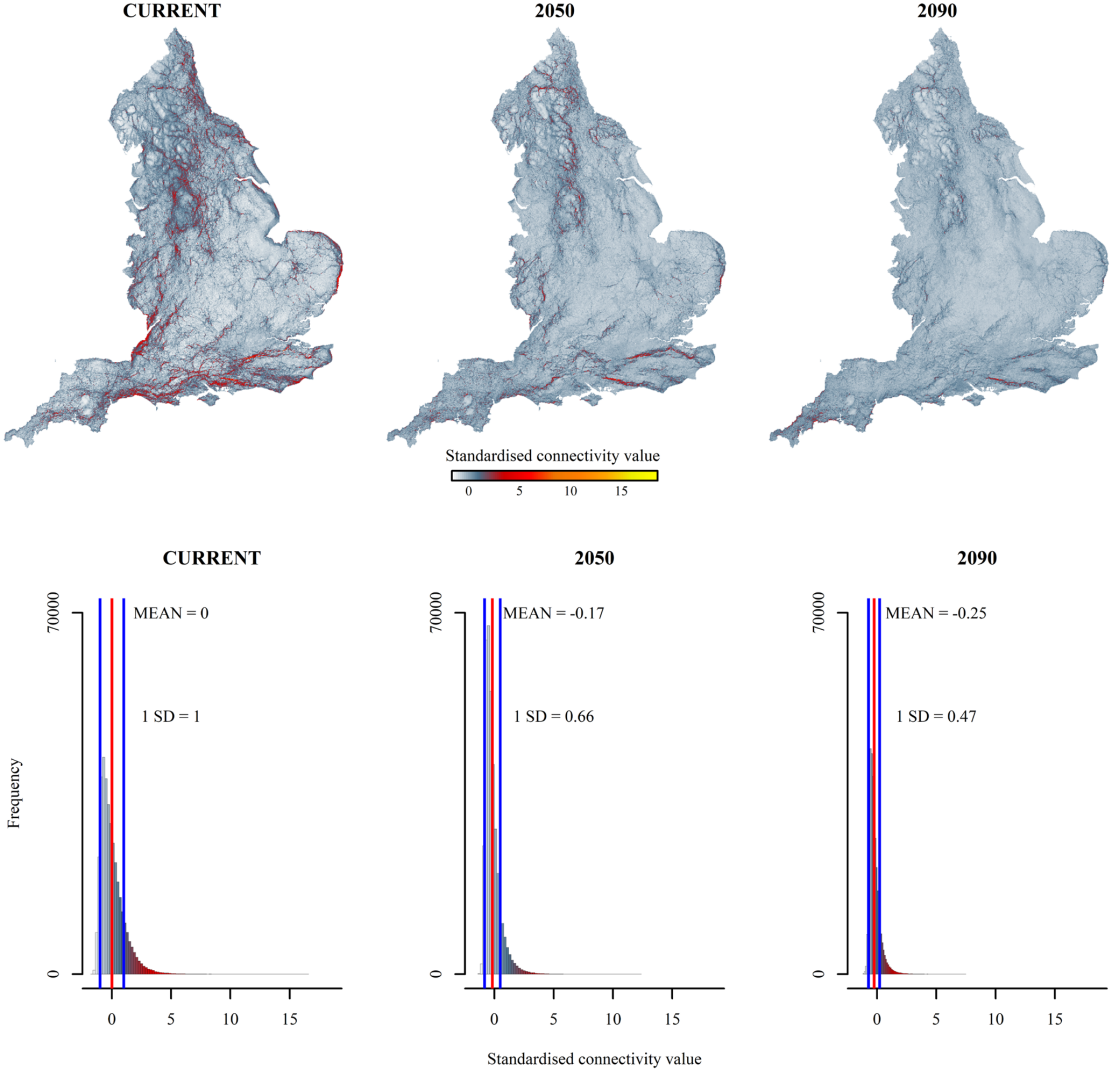
Connectivity for each climate scenario. Top row: Connectivity maps, stacked for all species, for current climate and future years 2050 and 2090. Pixel values in each scenario are *z*‐scores, standardised to current climate. Legend shows standardised connectivity units. Bottom row: Frequency histograms of the map pixels, i.e., frequency of the *z*‐scores for each scenario. Each histogram also shows its mean (red line) and 1 standard deviation (SD) (blue line) with its values.

Stacked species maps showed that with current climate, distinct high connectivity areas were to the Pennines northwards, the south coast of England, and patches of central England. This pattern was similar for 2050 and 2090; although high connectivity diminished in central England compared to that of the north and south (Figure 3).

Connectivity value frequency histograms showed positively skewed distributions with long tails of higher values (Figure 3). Summary statistics showed maximum connectivity values decreased from 18.57 to 9.73 between current and 2090 scenarios (Table 4). The mean value shifted to negative, i.e., left‐wise on the histogram. Ranges, standard deviations, and variances all decreased: a narrowing of the spread of connectivity values, suggesting that the country would become more permeable, although there were still some areas that were highly important for connectivity. Skewness values increased in 2050, and a weaker increase in 2090, i.e., shifting to the left of the histogram. Kurtosis values increased in 2050, i.e., a taller histogram, then decreased slightly in 2090. Pearson correlation values were: Current:2050 = 0.75; 2050:2090 = 0.86; Current:2090 = 0.68. Thus, comparing current climate with 2050, there was a future reduction of the higher connectivity flows; an increase in frequency of diffuse (lower connectivity value) pathways. This pattern continued, though more weakly, from 2050 to 2090.

**TABLE 4:** Summary statistics of histograms of connectivity for climate scenarios. Values for each scenario, standardised by *z*‐score units for current climate.

	Current climate	Year 2050	Year 2090
Min	−1.67	−1.41	−1.21
Max	18.57	12.99	9.73
Range	20.24	14.40	10.94
Mean	0.00	−0.17	−0.25
Standard deviation	1.00	0.66	0.47
Variance	1.00	0.44	0.22
Skewness	1.71	2.30	2.07
Kurtosis	8.39	12.93	11.53

### 3.5 The Landscape Connectivity Network

**FIGURE 4:**
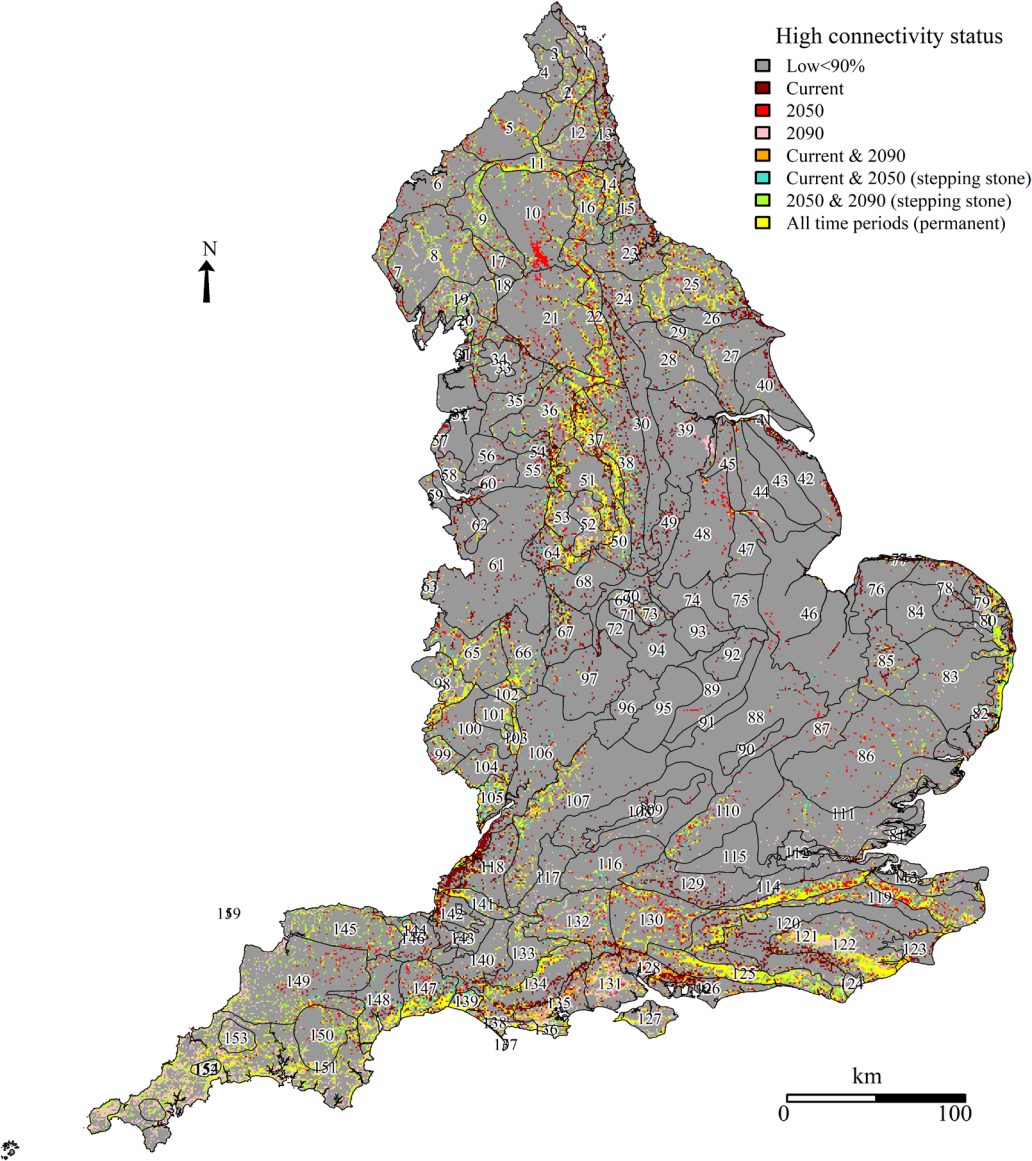
The landscape connectivity network elicited from top decile connectivity for England across three time periods. The landscape connectivity network comprised top decile areas of standardised connectivity values, for all time periods (‘permanent’), and ‘current & 2050’ and ‘2050 & 2090’ (i.e., ‘stepping stones’). Top decile connectivity locations for the other time periods are also shown but are regarded as impermanent and are not included in the landscape connectivity network. NCA shape locations are indicated numerically.

**FIGURE 5:**
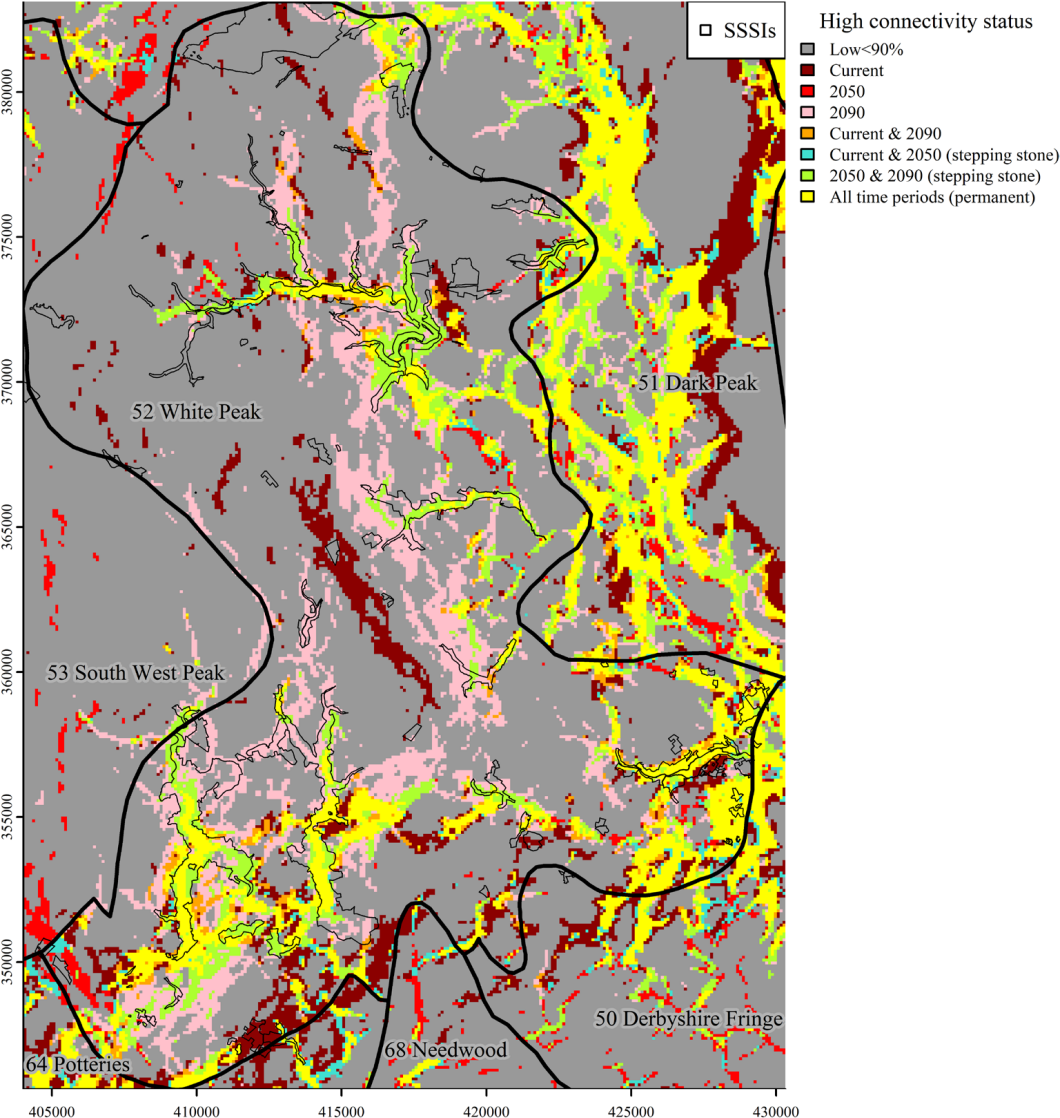
Excerpt from the landscape connectivity in Figure 4, for the White Peak. Only ‘All years permanent’ and ‘Current & 2050’ and ‘2050 & 2090’ stepping stone areas are included in the landscape connectivity network. Top decile connectivity locations for the other time periods are also shown but are regarded as impermanent and are not included in the landscape connectivity network. SSSIs are indicated by the black outlines. Axes are British National Grid references.

The landscape connectivity network comprised top decile areas common to all scenarios (‘permanent’) and those for current/2050 and 2050/2090 ‘stepping stones’ (Figure 4). The geography of the landscape connectivity network occurred mostly in the north, stretching down the western part of the country to the south of England. There were distinctly concentrated patterns within some NCAs, e.g., 121 Midvale Ridge, 125 South Downs, and 37 Yorkshire South Pennine Fringe (Table 5, Table S3). The White Peak showed substantial high connectivity land during current climate and 2090 scenarios (Figure 5). In the overall landscape connectivity network (i.e., permanent and stepping stone connectivity), the area ranked 39th of NCAs. Nearby NCAs also showed high connectivity: 50 Derbyshire Peak Fringe, 51 Dark Peak, 64 Potteries, and Churnet Valley; together, a cluster for species moving to or through northern England.

**TABLE 5:** NCAs ranked by the amount of landscape connectivity network as proportionate to area. NCA identity number and total size in hectares. Table shows top 10 and bottom 10 NCAs. For the full table of NCAs, refer to Table S1.

NCA Identity Number and Name	Total area, hectares	In the Landscape Connectivity Network								
		Rank	Percent of area	Area, hectares						
				Total	Broadleaved woodland	Coniferous woodland	Arable	Improved grassland	Calcareous grassland	Acid grassland
*Top 10*										
103 Malvern Hills	8331	1	56.50	4705	2042	60	77	1871	41	136
154 Hensbarrow	11,946	2	53.70	6414	2099	10	38	1760	—	4
139 Marshwood and Powerstock Vales	15,956	3	48.30	7702	1474	67	493	4554	174	1
125 South Downs	101,904	4	41.20	41,967	10,287	1023	6557	5864	14,627	—
37 Yorkshire Southern Pennine Fringe	58,553	5	37.40	21,921	5991	215	176	4780	265	275
105 Forest of Dean and Lower Wye	31,413	6	37.30	11,715	7169	1314	141	1710	30	36
136 South Purbeck	11,860	7	32.90	3905	681	40	222	1391	1275	—
11 Tyne Gap and Hadrian's Wall	43,455	8	31.80	13,810	3602	201	635	7358	107	109
119 North Downs	137,437	9	28.90	39,703	15,317	1266	3261	8330	5183	—
147 Blackdowns	80,859	10	28.70	23,194	7208	983	643	11,443	285	183
*Bottom 10*										
112 Inner London	33,023	149	0.10	31	2	—	—	8	—	—
114 Thames Basin Lowlands	32,797	149	0.10	32	22	1	—	1	—	—
75 Kesteven Uplands	69,038	154	0.00	30	14	—	—	—	—	—
91 Yardley‐Whittlewood Ridge	33,797	154	0.00	2	1	—	—	—	—	—
95 Northamptonshire Uplands	101,210	154	0.00	1	—	—	—	—	—	—
96 Dunsmore and Feldon	70,648	154	0.00	32	5	—	—	—	—	—
115 Thames Valley	86,107	154	0.00	14	—	—	—	2	—	—
158 Isles of Scilly	1636	157	0.00	—	—	—	—	—	—	—
159 Lundy	451	158	0.00	—	—	—	—	—	—	—
90 Bedfordshire Greensand Ridge	27,350	159	0.00	—	—	—	—	—	—	—

**FIGURE 6:**
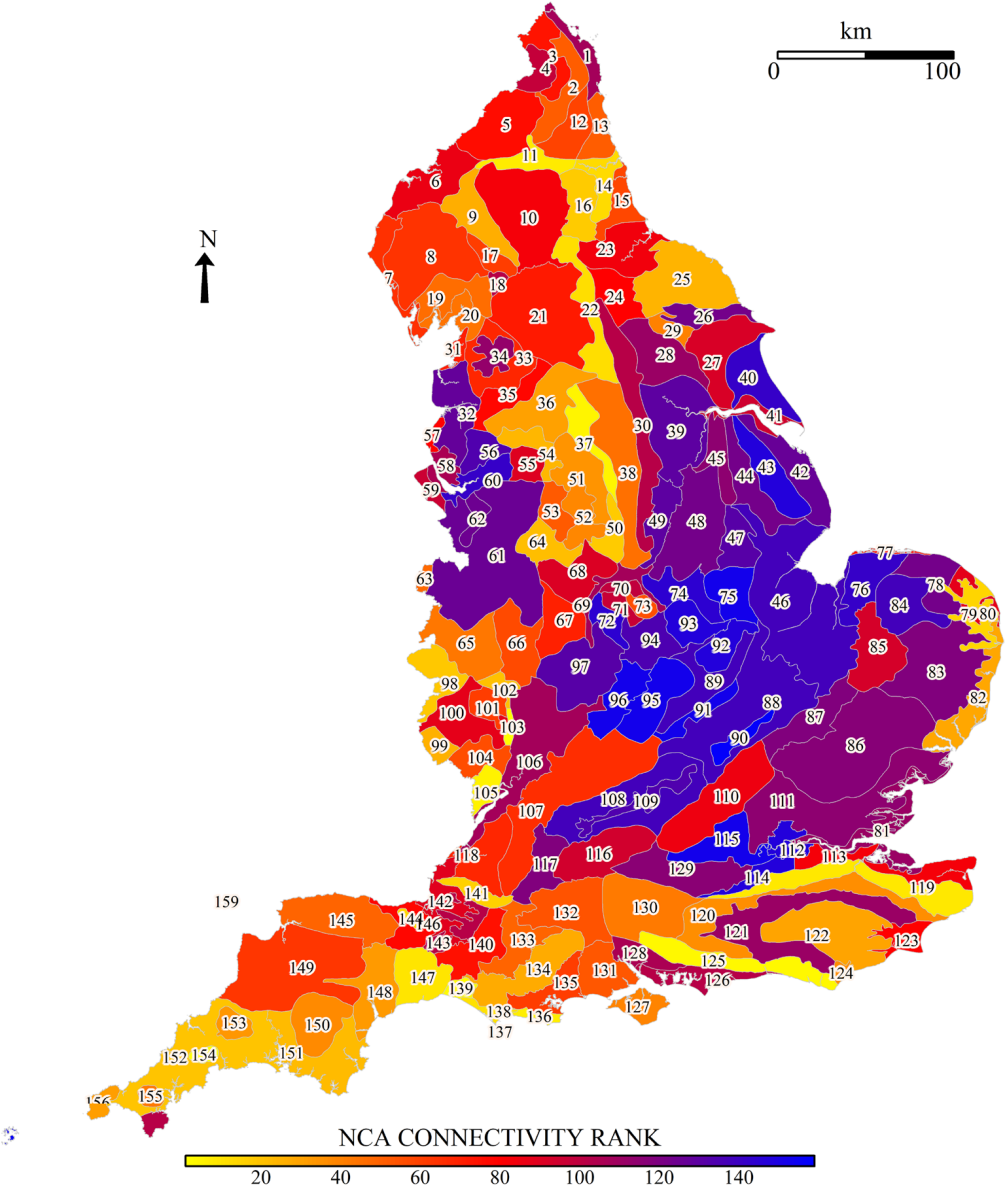
Ranking of the presence of the landscape connectivity network, proportionate to the NCA area. Groups of higher‐ranked NCAs occur in the South West, the South East, southern Pennines, and the North East. Higher connectivity‐ranked NCAs are displayed with warmer colors, whereas lower connectivity‐ranked NCAs are shown by cooler colors.

The ranked proportion of landscape connectivity network land within each NCA showed a clear north–south split (Figure 6, Table S3). Some important areas grouped together: North East: 11 Tyne Gap and Hadrian's Wall, 14 Tyne and Wear Lowlands, 16 Durham Coalfield Pennine Fringe; South East: 119 North Downs and 125 South Downs; West: 103 Malvern Hills, 105 Forest of Dean and Lower Wye; South West: 154 Hensbarrow, 147 Blackdowns, 139 Marshwood and Powerstock Vales (highest for the country); East of the White Peak: 37 Yorkshire South Pennine Fringe, 22 Pennine Dales Fringe. There was also an isolated area of connectivity along the coastal areas of East Anglia: 79 North East Norfolk and Flegg, 80 The Broads, and 82 Suffolk Coast and Heaths.

There were substantial future climate connectivity losses for 118 Bristol, 126 South Coast Plain, 42 Lincolnshire Coast and Marshes, and 1 North Northumberland Coastal Plain. There were increases throughout Devon, Cornwall, Sussex, and Cumbria.

**FIGURE 7:**
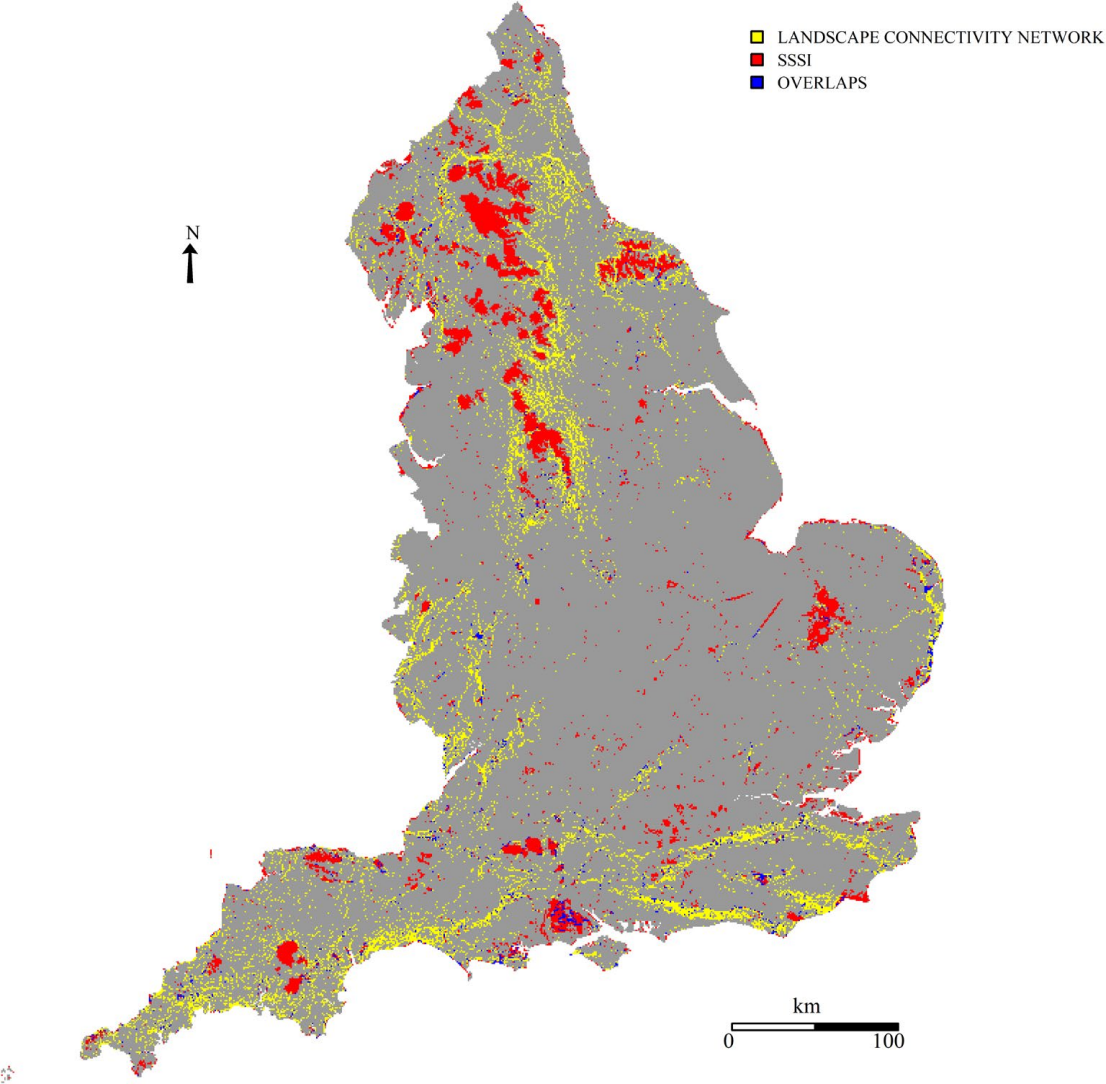
The landscape connectivity network (yellow pixels), compared to the SSSI network (red pixels), with overlapping areas (blue pixels).

The landscape connectivity network measured 1,029,000 ha, of which 13% lay within SSSIs (Table 6, Figure 7). Landcover within the network comprised 365,000 ha broadleaved woodland, with 17% within SSSIs; improved grassland comprised 305,000 ha, with 7% within SSSIs; and calcareous grassland comprised 55,000 ha, with 25% within SSSIs (Table 6). Arable land and other landcover categories each comprised < 5% total high connectivity. Some NCAs had proportionately larger amounts of landcover types; notably, 119 North Downs and 125 South Downs together possessed 36% of the calcareous grassland, and combined with 122 High Weald, 11% of the broadleaved woodland (Table 5, Table S3).

**TABLE 6:** Hectares of landscape connectivity network, by UK CEH landcover, outside and within SSSI network. Figures are shown for England and White Peak.

Category	England		White Peak	
	Outside SSSI	Within SSSI	Outside SSSI	Within SSSI
Broadleaved woodland	301,781	64,145	1245	663
Coniferous woodland	35,918	5401	124	74
Arable	48,468	2674	—	4
Improved grassland	282,409	23,393	297	1178
Neutral grassland	3487	1517	4	4
Calcareous grassland	41,746	13,914	1067	2012
Acid grassland	10,814	3578	18	73
Fen, marsh, swamp	4148	3930	—	—
Heather	1546	4979	10	7
Heather grassland	3004	2206	—	—
Bog	107	278	—	—
Inland rock	3719	390	37	250
Freshwater	14,083	5411	9	74
Urban	24,619	964	1	38
Suburban	117,053	3666	90	402
Total	892,902	136,446	2902	4779

Where the landscape connectivity network occurred in the White Peak, 38% was within SSSIs. In the White Peak, the majority of connectivity land comprised calcareous grassland (3079 ha; 35% = SSSI), broadleaved woodland (1908 ha; 65% = SSSI), and improved grassland (1475 ha; 20% = SSSI) (Table 6).

## 4 Discussion

Assessing a sample of 15 different generalist and specialist species from different taxonomic groups, attributed to occupying limestone and upland habitats, we found clear associations with the White Peak and northern uplands, and areas further afield, e.g., the south coast. This represents a credible landscape connectivity network for limestone and upland species for the entirety of England. Our models showed differing species responses to environmental characteristics and climate change, eliciting connectivity shifts through three scenarios: current, 2050, and 2090.

Testing our hypotheses, we found:

H1: Climate change results in substantial shifts in high connectivity value land for species across England. *Validated.* Current climate connectivity was concentrated on the south coast, the west, and the central and northeastern Pennines. For 2050, this pattern continued; connectivity became more diffuse and less concentrated, continuing weakly to 2090, then with central and eastern England showing low connectivity values, dividing the country into two regions: north and south. Most species showed climate change‐induced connectivity shifts. There were clear expansions for chalk carpet moth, hazel dormouse, water vole, and adder. Changes were slightly mixed for dingy skipper, white letter hairstreak, hedgehog, and Leisler's bat. There were large connectivity losses for northern brown argus, curlew, dipper, marsh tit, twite, willow tit, and otter.

H2: A large portion of high connectivity value land is designated SSSI. *Not validated.* Most (87%) high connectivity land was outside the SSSI network. This was likely a function of the species sample based on upland and limestone habitats. The SSSI network was conceived in the 1980's, whereas the landscape connectivity network reflected species responses to future climate change forecasts. This suggests there is scope to increase the amount of protected land in England that can support natural connectivity.

H3: A large portion of high connectivity value land is improved grassland. *Validated.* This proportion was 29%. Improved grassland often represents low biodiversity value, not beneficial to wildlife (Isbell et al. 2013), though potentially enabling movement. To achieve better natural connectivity, improved grassland could be transformed into other habitat types with a higher propensity for biodiversity. For example, chalk carpet moth, dingy skipper, and northern brown argus likely require calcareous grassland; marsh tit and willow tit require broadleaved woodland.

H4: The White Peak has a relatively large proportion of high connectivity value land within the SSSI network. *Validated.* This area possessed 7600 ha of the landscape connectivity network, 38% of which was within SSSIs. Considering the individual species maps we generated, specialist species associated with calcareous grassland had high connectivity in the White Peak, compared with much of England, e.g., Lepidoptera. Species associated with rivers, i.e., dipper, otter, and water vole, had high connectivity in the White Peak valleys, where there are the Rivers Wye, Dove, and Manifold. Some generalist species were also found to have high connectivity requirements in the White Peak: hedgehog and Leisler's bat. Some species had high connectivity in the White Peak today but not in 2050 and 2090, e.g., curlew, twite, and willow tit, each showing strong responses to climate variables and generally shifting connectivity to other parts of England.

H5: Most remaining White Peak high connectivity value land outside SSSIs is improved grassland. *Partly validated.* There were 4779 ha of unprotected (non‐SSSI) landscape connectivity network of which: calcareous grassland = 2012 ha, improved grassland = 1245 ha, broadleaved woodland = 663 ha. Natural connectivity could be improved by extending the SSSI extent; and encouraging alternatives to improved grassland.

### 4.1 Ecological Modelling and Technical Considerations

Connectivity assessments vary for species with different ecological characteristics, scales of movement, and dispersal. Our analysis for each species followed identical methods and may overlook nuanced species complexities. We tailored the connectivity analysis for each species only by applying different resistance values, informed by the literature. Furthermore, tailoring of resistance surfaces ideally requires empirical covariate evidence: movement telemetry, population density, genetic structure. When available, connectivity can be parameterised or scaled, matching closely with true species locations (e.g., Cushman and Lewis 2010; Proctor et al. 2015). This is important because some species have different tolerances to unsuitable habitat, which influences the amount of land considered to facilitate connectivity. e.g., Caroll et al. (2020) showed wide‐ranging wolverines across the western United States had a strong negative exponential relationship with habitat quality, i.e., once outside of home range they were only moderately sensitive to changes in habitat. However, such data is often lacking in the UK, suggesting a need for telemetry studies on important vagile species.

Our models aggregated the sum of species connectivity equally. Yet the prevalence and population trajectories of some species (e.g., number of records) were low (e.g., northern brown argus, twite), while others were relatively high (e.g., hedgehog, marsh tit). Our combined connectivity map did not weight for these differences, although weighting might present other problems by biasing results to more frequently recorded species. There may be debates regarding the species chosen, although we consider the fifteen used in this analysis to be representative of species within the focal study area of White Peak. Thus, there is a bias in this sample to species that favour uplands and calcareous grassland, and a connectivity network based on another group of species might be markedly different. Finally, some species may have particular roles (e.g., predator–prey, competitor, seed‐disperser), and our modelling could be advanced in the future by considering trophic structure and species functions (e.g., Mendoza and Araujo 2025), as well as representativeness, complementarity, and community composition (Wilson et al. 2009).

Our maps indicate connectivity flows, based on species distribution models. Yet connectivity is also sensitive to the uncertainty of parameters, including dispersal distances, species traits, landscape composition, or configuration (Prima et al. 2024). Spatial and movement requirements may vary in practice for taxonomic groups: e.g., simplistically, mammals need larger spaces, butterflies several smaller spaces. Some species are dispersal‐limited (e.g., hazel dormouse, water vole, adder), with very gradual changes in distribution. Connectivity means movement and migration, which are risky strategies and increase vulnerability. We cannot easily factor how climate range may affect metapopulation dynamics (e.g., mortality, competition, predation—see Gillies et al. 2025) nor how fast species may adapt physiologically for persistence (e.g., Crick 2004; Gunderson and Stillman 2015). Some species may actually have already started range shifting.

Whilst these SDM analyses were robust, as with so many such models, they were sensitive to choices of data and parameters. Models responded differently to choices of predictor variables: too large a proportion of climate variables provided wide, unrealistic projections of species ranges; whilst too large a proportion of land‐based variables constrained species ranges, obscuring climate change range shifts (Fournier et al. 2017), we chose to use a mix of climate variables and just majority landcover types, which appeared to achieve a balance. This work could be improved by examining factorial combinations of variables in pursuit of the best model (Zeller et al. 2017).

Circuit theory assessment resulted in quantified measures of connectivity (units of current: amperes), comprising mostly low values, with extreme tails of high values. Circuitscape literature often portrays results with histogram equalisation or decile stretch, visually accentuating differences (McRae et al. 2013). Yet histogram equalisation applies non‐linear normalisation to achieve a uniform distribution, rendering relative comparisons between time periods difficult. Instead, we found *z*‐score unit standardisation, supported by a visualisation palette of colours with diverging contrasts at the very bottom of this histogram scale, more clearly revealed absolute values and relative changes.

Our MESS analysis showed predictions for 2090 using fewer climate predictors within model calibration ranges for southern England. This is a function of underlying climate change forecast values with consequent uncertainty for ecology. For example, the temperature range for all England for BIO 1 (annual mean temperature) is current climate 5°C–11°C and 2090 climate 11°C–15°C. Most of the higher temperatures occur on the English south coast, meaning these areas are outside the current climate model calibration range, and thus their future species distributions are not predicted with this variable. Likewise, BIO 4 (temperature seasonality) and BIO 15 (precipitation seasonality) have narrow ranges which do not entirely overlap between current and future climates for England (Supporting Information). Hence, further connectivity modelling might reference SDMs for Great Britain (i.e., including Wales and Scotland), providing a wider range of climes and species distributions, so the resultant models are more accurate; or employing species data for wider geographies (e.g., Europe) which might show species more tolerant to climate change (e.g., Pearce‐Higgins et al. 2017).

Our future climate scenario models did not incorporate the possibility of climate‐induced habitat changes as predictors. This is a complex sphere of research with mixed results. For example, Trinder et al. (2020) articulated different changes in climate that influenced plant fitness, such that the composition of future habitat types is hard to predict. Yu et al. (2021) forecast substantial differences in responses by tree species to climate scenarios, varying geographically; predictions confounded by the effects of pests, pathogens, fire, and management strategies.

Creating a stacked connectivity model aggregated some responses and obscured some differences (e.g., chalk carpet moth, hazel dormouse, adder similar connectivity on south coasts; contrasting with marsh tit and Leisler's bat more widespread; curlew, dipper, twite, very northern). Nonetheless, the overall combined species models, summarised by the landscape connectivity network, used the reported connectivity values given for each species. Whilst some of the values are relatively low, e.g., hazel dormouse, white letter hairstreak, and some high, e.g., hedgehog, by treating all species the same and then combining them, we may consider this wholly representative.

Comparison of our findings with others is challenging, amidst a plethora of data sources, scales, climate change scenarios, and analytical methods. Our modelled responses of species to climate change were broadly consistent with the literature (Conroy and Chanin 2000; Dunford and Berry 2012; Morecroft and Speakman 2015; Pearce‐Higgins et al. 2017; Fox et al. 2021; Fox et al. 2022). Evidence shows that UK species are redistributing northwards and to higher elevations (Thomas et al. 2012; Mason et al. 2018); those having warm southerly distributions are expanding, with expectations that species unable to shift distributions at the pace of climate change may experience range reductions (Johnston et al. 2013; Cunningham et al. 2021).

### 4.2 Application of the Landscape Connectivity Network

We have designed this landscape connectivity network for upland and limestone species for England across three climate change scenarios: current climate, 2050, and 2090. We have ranked these by NCA, showing the amount of protected land (i.e., SSSI) and the landcover types these encompass. Our case study for the White Peak shows a national model applied to regional connectivity, highlighting to the hectare, areas which may merit conservation intervention.

Our connectivity maps represent models of ecological processes. Our landscape connectivity network selected high connectivity value spaces with climate analogues occurring across two ‘stepping stone’ periods (current/2050; 2050/2090) or three consecutive ‘permanent’ time periods (current/2050/2090). These were conservative practical choices, alterable for different management goals. Our modelled network could be used for choosing spaces for protection or habitat restoration, involving selection algorithms which can configure reserves based on spatial or temporal contiguity, to achieve minimum dispersal corridors (Williams et al. 2005). There are other alternatives to define the landscape network, e.g., to additionally include top decile areas for current climate, 2050, and 2090, or, by contrast, exclude isolated areas not spatially contiguous to current/2050/2090. We highlight these choices, enabling consideration by stakeholders and land managers whose objectives may differ.

Comparing the landscape connectivity network with the NCA framework has been informative, often with clear connectivity flows, resembling NCA shapes. The NCA mapping project used wider environmental data without climate and species information. Yet, its resemblances to our connectivity maps suggest that the underlying landscape character of these areas is widely applicable. NCAs appear as land facets—recurring landscape units with uniform topographic and soil attributes—known to have strong predictive value (Beier and Brost 2009). Their connectivity rankings will be different for other samples of species.

Our results showed just 13% of the landscape connectivity network lay within the SSSI map. This compares similarly to connectivity models of Travers (2021) who found 10% of priority habitat patches were protected. Although there are differences in land cover definitions and mapping resolutions, both studies suggest substantial areas of ecological connectivity value in England lack protection.

This research could be further developed by assessing different groups of species associated with other habitat types, or tailoring models for unusual species, or modelling climate change over more time periods. In the UK, the inception of species telemetry programs would help validate results, providing better information about the functional connectivity for species moving through suitable and unsuitable habitats, which may validate these results. Connectivity models such as these help to inform the identification of protected areas, landcover restoration, species recovery, translocations, infrastructure planning, roadkill mitigations, and other human pressures. As several of our species are considered rare, intervention to assist them may also provide support to those more common.

Overall, our approach enables the identification of connectivity areas, consistent with the ‘better, bigger, more, and joined’ vision of landscape connectivity networks to conserve biodiversity across England (Lawton et al. 2010). This research demonstrates that combining the analyses for a range of species, can help to identify areas commonly important for connectivity. By linking such areas into the protected sites network, and improving habitats, conservation efforts can enhance the persistence of wildlife in the face of climate change.

## Author Contributions

**Carlos P. E. Bedson:** conceptualization (lead), data curation (lead), formal analysis (lead), investigation (lead), methodology (lead), project administration (lead), validation (lead), visualization (lead), writing – original draft (lead), writing – review and editing (lead). **Ben L. Payne:** formal analysis (supporting), methodology (supporting), writing – review and editing (supporting). **Chris Sutherland:** formal analysis (supporting), validation (supporting), visualization (supporting), writing – review and editing (supporting). **Heather E. White:** methodology (supporting), visualization (supporting), writing – review and editing (supporting). **Danielle J. Greaves:** conceptualization (supporting), formal analysis (supporting), writing – original draft (supporting), writing – review and editing (supporting). **Fraser Buchanan:** resources (supporting), software (equal). **Humphrey Q. P. Crick:** conceptualization (supporting), methodology (supporting), supervision (supporting), validation (supporting), visualization (supporting), writing – original draft (supporting), writing – review and editing (supporting).

## Conflicts of Interest

Carlos P. E. Bedson, Ben L. Payne, Danielle J. Greaves, Heather E. White, Fraser Buchanan, and Humphrey Q. P. Crick work for UK government agencies with a remit to support increasing biodiversity in England.

## Supporting information


**Appendix S1:** ece371956‐sup‐0001‐AppendixS1.pdf.

**Data S1:** ece371956‐sup‐0002‐Supinfo.zip.

## Data Availability

The data that support the findings of this study are available from third party sources as listed in references. Certain restrictions may apply to the availability of these data, depending on the access terms and conditions of some sources. For specific advice, please consult the corresponding author.
