# Supplementary material for: Designing a Climate Change Resilient Landscape Connectivity Network From a Multi‐Species Perspective
Source: Ecol Evol. 2025 Sep 18;15(9):e71956. doi: 10.1002/ece3.71956 (PMC12446580; doi:10.1002/ece3.71956)
Supplement: Supplementary file 1 — Appendix S1: ece371956‐sup‐0001‐AppendixS1.pdf. [file ECE3-15-e71956-s002.pdf]

SUPPORTING INFORMATION DESCRIPTION

The zip file contains four pdf documents which support the analysis.
TABLE_S1_NCA_ID_LIST
FIGURE_S1_MESS
FIGURE_S2_AND_TABLE_S2_SPECIES_RESISTANCE_PARAMETERS
TABLE_S3_NCA_CONNECTIVITY_RANKING

The zip file also contains four folders with files which may be viewed to help interpreting models and data which support the summarised analysis. The files are high resolution .tif format, enabling zoom-in viewing. These are:

ENVIRONMENTAL.VARIABLE.PREDICTOR.PLOTS
Separate files showing environmental variables considered for SDMs.

SDM.VARIABLE.IMPORTANCE.PLOTS
Folder contains biomod2 generated variable importance plots for each species distribution model, as viewable .tif files, with file names for each species. Charts show how important each variable is to the total prediction, based on shuffling method in biodmod2. The x-axis shows the variable. The y-axis shows the % correlation of the variable with the projection. EMca is the committee average ensemble model and Emwmean is the weighted mean ensemble model each on a scale from 0 (not correlated) to 100 (fully correlated). Emcv is the coefficient of variation of the ensemble model i.e. the level of uncertainty on a scale from 0 (none) to 100 (high).

SDM.VARIABLE.RESPONSE.PLOTS
Contains biomod2 generated variable response plots for each species distribution model, as viewable .tif files referencing file names for each species. Each plot shows the x-axis with predictor variable and values; the y-axis has the response from 0 (not present) to 1.00 (present). X-axis values vary according to the type of predictor. Climate values are WorldClim temperature, precipitation or variation as listed in the main paper table 2. Landcover, rivers and roads are distance to feature expressed in metres. TPI is an abstraction of elevation relative to neighbouring locations, identifying high mountain tops or plateaus, valley sides and bottoms. EMca is the committee average ensemble model and EMwmean is the weighted mean ensemble model.

CONNECTIVITY.PLOTS.SPECIES
Contains biomod2 ensemble species distribution models and Circuitscape generated connectivity analyses for each species, as viewable .tif files referencing file names for each species. For each plot: Top row 1 = species distribution model. Legend = probability of occurrence on scale 0 (low) to 100 (high). Second row = Connectivity analysis normalised. Legend scale varies 0=low (light blue) with max (yellow) differing per species. Third row = White Peak zoom in of species distribution model. Bottom row = White Peak zoom in of connectivity analysis. Columns represent models which reference current climate variables, year climate 2050

variables and year 2090 climate variables, with all landcover, rivers, roads and TPI held constant in each scenario. Current and WP SDMs show species records as black dots. White Peak outline shown in each plot in black.
