# Supplementary material for: Designing a Climate Change Resilient Landscape Connectivity Network From a Multi‐Species Perspective
Source: Ecol Evol. 2025 Sep 18;15(9):e71956. doi: 10.1002/ece3.71956 (PMC12446580; doi:10.1002/ece3.71956)

Figure S1 Multi Environment Singularity Surface (MESS) for SDMs, England, by climate scenario (current climate, 2050 and 2090). Colours and values in the legend show the number of climate variables invoked per pixel for the prediction. These are in addition to landcover, rivers, roads and tpi.

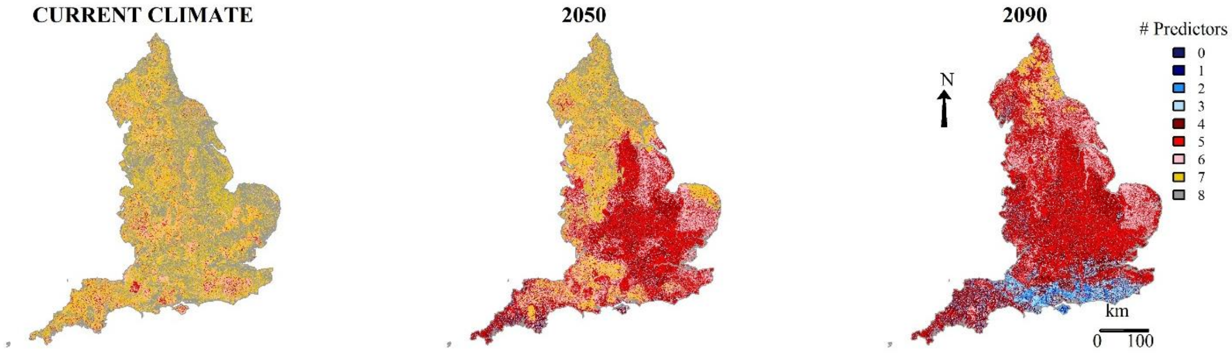

Supplement: Supplementary file 2 — Data S1: ece371956‐sup‐0002‐Supinfo.zip. [file ECE3-15-e71956-s001.zip › SUPPORTING.INFORMATION/FIGURE_S1_MESS.pdf]
