# Supplementary material for: Designing a Climate Change Resilient Landscape Connectivity Network From a Multi‐Species Perspective
Source: Ecol Evol. 2025 Sep 18;15(9):e71956. doi: 10.1002/ece3.71956 (PMC12446580; doi:10.1002/ece3.71956)
Supplement: Supplementary file 2 — Data S1: ece371956‐sup‐0002‐Supinfo.zip. [file ECE3-15-e71956-s001.zip › SUPPORTING.INFORMATION/FIGURE_S2_AND_TABLE_S2_SPECIES_RESISTANCE_PARAMETERS.pdf]

Species Resistance Parameters

Resistance surfaces values were considered following Keeley et al. 2017, replicated here.

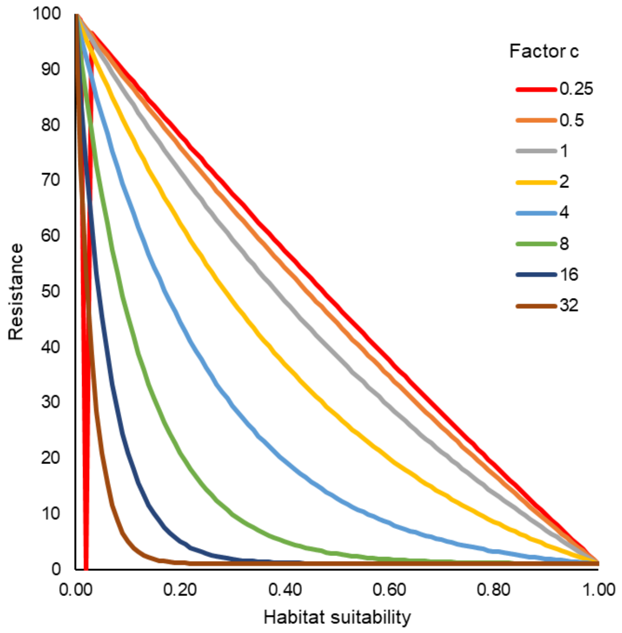

Figure S2 Graph showing transformation of SDM values to resistance surface. x-axis shows SDM values, i.e. habitat suitability on a scale 0 to 1. y-axis shows resistance values. Legend shows exponential "factor c" value.

Calculations made with formula:

$$R = 100-99*((1- \exp(-c*H))/1-\exp(-c)))$$

Where R is resistance, H is suitability and the factor c determines the shape of the curves.

We consulted the following summary and literature, to consider appropriate exponential values for transforming SDMs to resistance surfaces. For species with medium (-2) and high (-4) resistance values, we also tested higher and lower values above these points, to gauge sensitivity of connectivity outputs, to informed the eventual choice of resistance value.

Table S2 Literature summary of species habitat preferences and dispersal distances.

Species	Habitat preference	Dispersal distance	Summary	Resistance value
**Butterflies and moths**				
Chalk Carpet	Specialist	Several km	Localised to calcareous sites (Randle et al. 2019)	High -0.25
Dingy Skipper	Specialist	Several km	Associated with open sunny habitats, chalk downland, woodland rides and clearings, coastal habitats, dunes, undercliffs, heathalnd, old quarries, railway lines and waste ground; occurs in discrete colonies, <50 individuals; sedentary; unlikely to colonise new areas of habitat unless close to existing populations, though a few individuals may move several km; vulnerable to changes in habitat management; conservation depends on maintaining habitat patches over large areas (Asher et al. 2001).	High -0.25
Northern Brown Argus	Specialist	<1km	Small discrete colonies (<200 adults) breed on patches <1ha; highly sedentary moving 20-30m over several days or between patches up to 150m; very limited colonising ability; limited by decreasing availability of unimproved grssland habitats; associated with common rock rose *Helianthemum nummularium* and taller vegetation (Asher et al. 2001).	High -0.25
White Letter Hairstreak	Specialist	Several km	Occurs in sheltered hedgerows, mixed scrub, woodland rides and associated with elm trees; adults regularly move between trees 300m apart, individuals may move several km (Asher et al. 2001).	High -0.25
**Birds**				
Curlew	Specialist	~600km	Dipersal <600km (Schwemmer et al. 2021); wintering period ~8 months; exploiting large geographical area; high intra-annual site fidelity; small, variable home ranges ~500 ha; high diversity of coastal and inland habitats (Donnez et al. 2023)	Medium -2
Dipper	Specialist	~4km	Highly specific habitat requirements, conditioned and concentrated on rivers with high degree of flow variability where riffles and pools of deep water are found (Royan et al. 2010). Dispersal upstream/downstream = 75% / 58%; new tributary = 16% / 17%; across watersheds = 19% / 21%. Dispersal range mean=3km/4km, max=32km/ 17km. (Tyler et al. 2012 / Garlaza et al. 2023).	Medium -2
Marsh tit	Specialist	~ 1 km	Natal dispersal distance ~ 1km; most occurring in June; sensitive to habitat fragmentation, resulting in poor settling success outside of natal wood (Broughton et al. 2010)	High -0.25
Twite	Generalist		Breed in areas of low-intensity upland agriculture, managed moorland edge and rocky coasts, winter in seed-rich farmland and salt marsh (Raine 2006). Dispersal distance, mean=7km, max = 46km (Raine et al. 2006a) Populations at South Pennines and Lancs/Cumbria hve entirely different wintering and breeding grounds (Raine et al. 2006b). Recruitment between Great Britain and continental europe very low; within British Isles separate discrete breeding regions; British maritime climate allowing partial migration moving from inland to coast (Dunning et al. 2020)	Medium -2
Willow Tit	Specialist	~ 2 km	Natal dispersal distance ~2km; relies on decaying snags in mature deciduous forests; sensitive to forest management methods (Kumpula et al. 2023)	High -0.25
**Mammals**				
Hazel Dormouse	Specialist	~2km	Dispersal distance <2km; limited by habitat fragmentation; associated with diverse deciduous and mixed woodland, with a well-developed understorey; very specific requirements for habitat and food availability (Cartledge et al. 2024)	High -0.25
Hedgehog	Generalist	~3km	Dispersal distance mean = 3.5km, max = 8.46km; edge habitats represent movement corridors; follow road netowrks; tendency to disperse to urban areas (Doncaster et al. 2001). Home ranges ~ 1.6km^2 (Harris and Yalden 2008); Fragmented and genetically distinct populations in rural agricultural landscapes, poor dispersers; reliant on hedgerows or field margins; preference for urban areas; avoid agriculturally dominated areas (Yarnell and Pettett 2020)	Low -4

Leislers Bat	Generalist	~800km	Flight distances from roosts 3-7km, max=13km over pasture or canals (Shiel et al. 1999). Forage woodland and scrub-lined roads; mean forage distance ~4km; max daily travel 13km; migratory movements >800km (Harris and Yalden 2008).	Low -4
Otter	Specialist	~13km	Capable of long distance ~ 13km overland dispersal between watersheds; mean linear range ~ 48km (Harris and Yalden 2008); Dispersal distance, mean = 21km, max = 25km (Quaglietta et al. 2013)	Low -4
Water Vole	Specialist	~2km	Usually found within 2m of water's edge; home ranges ~114m; dispersal distance up to 2km including overland (Harris and Yalden 2008).	High -0.25
**Reptiles**				
Adder	Specialist		Populations are fragmented; found in grassland, heathland, fen, blanket, raised bog, maritime cliffs, sand dunes (Dunford and Berry 2012). Mean daily movement = 21m; max home range = 6.3 ha (Nash et al. 2018). Confined to specific habitats, woodland, heathland, moorland…sedentary, small home ranges, isolated... complex habitat requirements.. affected by human disturbance (Gardner et al. 2019). Max natal dispersal distance = 1300m (Francois et al. 2021)	High -0.25

References

Asher, J., Warren, M., Fox, R., Harding, P., Jeffcoate, G. & Jeffcoate, S. 2001. The Millenum Atlas of Butterflies in Britain and Ireland. Oxford University Press.

Broughton, R.K., Hill, R.A., Bellamy, P.E. & Hinsley, S.A. 2010. Dispersal, ranging and settling behaviour of Marsh Tits Poecile palustris in a fragmented landscape in lowland England. Bird Study (57) 458-472. https://doi.org/10.1080/00063657.2010.489316

Cartledge, E.L., Bellis, J., White, I., Hurst, J.L., Stockley, P. & Dalrymple, S. 2023. Current and future climate suitability for the hazel dormouse in the UK and the impact on reintroduced populations. *Conservation Science and Practice*, 6(12), e13254. https://doi.org/10.1111/csp2.13254

Doncaster, C. P, Rondinini, C.& Johnson , P.C.D. 2001. Field test for environmental correlates of dispersal in hedgehogs Erinaceus europaeus. *Journal of Animal Ecology*, *70*(1), pp.33-46. https://doi.org/10.1111/j.1365-2656.2001.00471.x

Donnez, M., Schwemmer, P., Fort, J. *et al.* Small Space but High Diversity: Spatial and Temporal Habitat Use by Endangered Eurasian Curlew at Wintering Sites Throughout Europe. *Wetlands* **43**, 80 (2023). https://doi.org/10.1007/s13157-023-01728-w

Dunford, R.W. & Berry, P.M. 2012. Climate change modelling of English amphibians and reptiles: Report to Amphibian and Reptile Conservation Trust. Environmental Change Institute, University of Oxford, Oxford UK.

Dunning, J., Finch, T., Davison, A. and Durrant, K.L., 2020. Population‐specific migratory strategies of Twite Linaria flavirostris in Western Europe. *Ibis*, *162*(2), pp.273-278. https://doi.org/10.1111/ibi.12791

François, D., Ursenbacher, S., Boissinot, A., Ysnel, F. & Lourdais. O. 2021 Isolation-by-distance and male-biased dispersal at a fine spatial scale: a study of the common Eu ropean adder (Vipera berus) in a rural landscape. Conservation Genetics, 22 (5), pp.823-837. https://hal.science/hal-03231063v1

Gardner, E., Julian, A., Monk, C. & Baker, J. 2019. Make the Adder Count: population trends from a citizen science survey of UK adders. Herpetological Journal 29: 57-70. https://doi.org/10.33256/hj29.1.5770

Garlaza, A., Betanzos-Lerraga, L. & Rodriguez, P. 2023. Natal and breeding dispersal of the White-throated Dipper *Cinclus cinclus* in coastal rivers of the northern Iberian Peninsula. Ringing and Migration. https://doi.org/10.1080/03078698.2023.2287559

Harris, S. & Yalden, D.W. 2008. Mammals of the British Isles. The Mammal Society, Southampton UK

Kumpula, S., Vatka, E., Orell, M. & Rytkönen, S. 2023. Effects of forest management on the spatial distribution of the willow tit (Poecile montanus).Forest Ecology and Management 529: 120694 https://doi.org/10.1016/j.foreco.2022.120694

Nash, D.J. and Griffiths, R.A., 2018. Ranging behaviour of adders (Vipera berus) translocated from a development site. *Herpetological Journal*, *28*(4), pp.155-159.

Quaglietta,L., Fonseca, V.C., Hájková, P., Mira,A., & Boitani, L. 2013. Fine-scale population genetic structure and short-range sex-biased dispersal in a solitary carnivore, Lutra lutra, Journal of Mammalogy, Volume 94, Issue 3, Pages 561–571, https://doi.org/10.1644/12-MAMM-A-171.1

Raine, A.F. 2006. The breeding ecology of Twite Carduelis flavirostris and the effects of upland agricultural intensification. PhD thesis, University of East Anglia, UK.

Raine, A. F., Sowter, D. J., Brown, A. F., & Sutherland, W. J. (2006a). Natal philopatry and local movement patterns of Twite *Carduelis flavirostris* . *Ringing & Migration*, *23*(2), 89–94. https://doi.org/10.1080/03078698.2006.9674350

Raine, A. F., Sowter, D. J., Brown, A. F., & Sutherland, W. J. (2006b). Migration patterns of two populations of twite carduelis flavirostris in Britain. *Ringing & Migration*, *23*(1), 45–52. https://doi.org/10.1080/03078698.2006.9674343

Randle Z, Evans-Hill LJ, Parsons MS, Tyner, A., Bourn, N.A.D., Davis, T., Dennis, E.B., O'Donnell, MI., Prescott, T., Tordofff, G.M. 7 Fox, R. 2019. Atlas of britain & Ireland's larger moths. Nature Bureau, Newbury UK.

Royan, A., Prudhomme, C., Hannah, D.M., Reynolds, S.J., Noble, D.G. & Sadler, J.P. 2015 Climate-induced changes in river flow regimes will alter future bird distributions. Ecosphere 6(4) Article 50 https://doi.org/10.1890/ES14-00245.1

Schwemmer, P., Mercker, M., Vanselow, K.H. *et al.* Migrating curlews on schedule: departure and arrival patterns of a long-distance migrant depend on time and breeding location rather than on wind conditions. *Mov Ecol* **9**, 9 (2021). https://doi.org/10.1186/s40462-021-00252-y

Shiel, C.B., Shiel, R.E. and Fairley, J.S. (1999), Seasonal changes in the foraging behaviour of Leisler's bats (*Nyctalus leisleri*) in Ireland as revealed by radio-telemetry. Journal of Zoology, 249: 347-358. https://doi.org/10.1111/j.1469-7998.1999.tb00770.x

Tyler, S. J., Ormerod, S. J., & Lewis, J. M. S. (1990). The post-natal and breeding dispersal of Welsh Dippers *Cinclus cinclus*. *Bird Study*, *37*(1), 18–22. https://doi.org/10.1080/00063659009477032

Yarnell, R. W., & Pettett, C. E. (2020). Beneficial Land Management for Hedgehogs (*Erinaceus europaeus*) in the United Kingdom. *Animals*, *10*(9), 1566. https://doi.org/10.3390/ani10091566
