# Supplementary material for: Designing a Climate Change Resilient Landscape Connectivity Network From a Multi‐Species Perspective
Source: Ecol Evol. 2025 Sep 18;15(9):e71956. doi: 10.1002/ece3.71956 (PMC12446580; doi:10.1002/ece3.71956)
Supplement: Supplementary file 2 — Data S1: ece371956‐sup‐0002‐Supinfo.zip. [file ECE3-15-e71956-s001.zip › SUPPORTING.INFORMATION/TABLE_S1_NCA_ID_LIST.pdf]

Table S1 National Character Area identity table. The reader may refer to this table when consulting manuscript figures e.g. Figure 1.

ID	NATIONAL CHARACTER AREA
1	North Northumberland Coastal Plain
2	Northumberland Sandstone Hills
3	Cheviot Fringe
4	Cheviots
5	Border Moors and Forests
6	Solway Basin
7	West Cumbria Coastal Plain
8	Cumbria High Fells
9	Eden Valley
10	North Pennines
11	Tyne Gap and Hadrian's Wall
12	Mid Northumberland
13	South East Northumberland Coastal Plain
14	Tyne and Wear Lowlands
15	Durham Magnesian Limestone Plateau
16	Durham Coalfield Pennine Fringe
17	Orton Fells
18	Howgill Fells
19	South Cumbria Low Fells
20	Morecambe Bay Limestones
21	Yorkshire Dales
22	Pennine Dales Fringe
23	Tees Lowlands
24	Vale of Mowbray
25	North York Moors and Cleveland Hills
26	Vale of Pickering
27	Yorkshire Wolds
28	Vale of York
29	Howardian Hills
30	Southern Magnesian Limestone
31	Morecambe Coast and Lune Estuary
32	Lancashire and Amounderness Plain
33	Bowland Fringe and Pendle Hill
34	Bowland Fells
35	Lancashire Valleys
36	Southern Pennines

37 Yorkshire Southern Pennine Fringe

38 Nottinghams, Derbys & Yorks Coalfield

39 Humberhead Levels

40 Holderness

41 Humber Estuary

42 Lincolnshire Coast and Marshes

43 Lincolnshire Wolds

44 Central Lincolnshire Vale

45 Northern Lincolnshire Edge with Coversands

46 The Fens

47 Southern Lincolnshire Edge

48 Trent and Belvoir Vales

49 Sherwood

50 Derbyshire Peak Fringe and Lower Derwent

51 Dark Peak

52 White Peak

53 South West Peak

54 Manchester Pennine Fringe

55 Manchester Conurbation

56 Lancashire Coal Measures

57 Sefton Coast

58 Merseyside Conurbation

59 Wirral

60 Mersey Valley

61 Shropshire, Cheshire and Staffordshire Plain

62 Cheshire Sandstone Ridge

63 Oswestry Uplands

64 Potteries and Churnet Valley

65 Shropshire Hills

66 Mid Severn Sandstone Plateau

67 Cannock Chase and Cank Wood

68 Needwood and South Derbyshire Claylands

69 Trent Valley Washlands

70 Melbourne Parklands

71 Leicestershire and South Derbyshire Coalfield

72 Mease/Sence Lowlands

73 Charnwood

74 Leicestershire and Nottinghamshire Wolds

75 Kesteven Uplands

76 North West Norfolk

77	North Norfolk Coast
78	Central North Norfolk
79	North East Norfolk and Flegg
80	The Broads
81	Greater Thames Estuary
82	Suffolk Coast and Heaths
83	South Norfolk & High Suffolk Claylands
84	Mid Norfolk
85	The Brecks
86	South Suffolk and North Essex Clayland
87	East Anglian Chalk
88	Bedfordshire and Cambridgeshire Claylands
89	Northamptonshire Vales
90	Bedfordshire Greensand Ridge
91	Yardley-Whittlewood Ridge
92	Rockingham Forest
93	High Leicestershire
94	Leicestershire Vales
95	Northamptonshire Uplands
96	Dunsmore and Feldon
97	Arden
98	Clun and North West Herefordshire Hills
99	Black Mountains and Golden Valley
100	Herefordshire Lowlands
101	Herefordshire Plateau
102	Teme Valley
103	Malvern Hills
104	South Herefordshire and Over Severn
105	Forest of Dean and Lower Wye
106	Severn and Avon Vales
107	Cotswolds
108	Upper Thames Clay Vales
109	Midvale Ridge
110	Chilterns
111	Northern Thames Basin
112	Inner London
113	North Kent Plain
114	Thames Basin Lowlands
115	Thames Valley
116	Berkshire and Marlborough Downs

117	Avon Vales
118	Bristol, Avon Valleys and Ridges
119	North Downs
120	Wealden Greensand
121	Low Weald
122	High Weald
123	Romney Marshes
124	Pevensey Levels
125	South Downs
126	South Coast Plain
127	Isle of Wight
128	South Hampshire Lowlands
129	Thames Basin Heaths
130	Hampshire Downs
131	New Forest
132	Salisbury Plain and West Wiltshire Downs
133	Blackmore Vale and Vale of Wardour
134	Dorset Downs and Cranborne Chase
135	Dorset Heaths
136	South Purbeck
137	Isle of Porland
138	Weymouth Lowlands
139	Marshwood and Powerstock Vales
140	Yeovil Scarplands
141	Mendip Hills
142	Somerset Levels and Moors
143	Mid Somerset Hills
144	Quantock Hills
145	Exmoor
146	Vale of Taunton and Quantock Fringes
147	Blackdowns
148	Devon Redlands
149	The Culm
150	Dartmoor
151	South Devon
152	Cornish Killas
153	Bodmin Moor
154	Hensbarrow
155	Carnmenellis
156	West Penwith

157 The Lizard
158 Isles of Scilly
159 Lundy
