# Supplementary material for: Designing a Climate Change Resilient Landscape Connectivity Network From a Multi‐Species Perspective
Source: Ecol Evol. 2025 Sep 18;15(9):e71956. doi: 10.1002/ece3.71956 (PMC12446580; doi:10.1002/ece3.71956)
Supplement: Supplementary file 2 — Data S1: ece371956‐sup‐0002‐Supinfo.zip. [file ECE3-15-e71956-s001.zip › SUPPORTING.INFORMATION/TABLE_S3_NCA_CONNECTIVITY_RANKING.pdf]

Table S3 NCAs ranked by amount of landscape connectivity network as proportionate to area. NCA identity number and total size in hectares. The six landcover types presented in this table do not sum to the total area, as 15 other landcover types are not included.

		In Nature Network Design								
		Rank	Percent of area	Area, hectares						
NCA Identity Number and Name	Total area, hectares			Total	Broadleaved woodland	Coniferous woodland	Arable	Improved grassland	Calcareous grassland	Acid grassland
103 Malvern Hills	8,331	1	56.50	4,705	2,042	60	77	1,871	41	136
154 Hensbarrow	11,946	2	53.70	6,414	2,099	10	38	1,760	-	4
139 Marshwood and Powerstock Vales	15,956	3	48.30	7,702	1,474	67	493	4,554	174	1
125 South Downs	101,904	4	41.20	41,967	10,287	1,023	6,557	5,864	14,627	-
37 Yorkshire Southern Pennine Fringe	58,553	5	37.40	21,921	5,991	215	176	4,780	265	275
105 Forest of Dean and Lower Wye	31,413	6	37.30	11,715	7,169	1,314	141	1,710	30	36
136 South Purbeck	11,860	7	32.90	3,905	681	40	222	1,391	1,275	-
11 Tyne Gap and Hadrian's Wall	43,455	8	31.80	13,810	3,602	201	635	7,358	107	109
119 North Downs	137,437	9	28.90	39,703	15,317	1,266	3,261	8,330	5,183	-
147 Blackdowns	80,859	10	28.70	23,194	7,208	983	643	11,443	285	183
138 Weymouth Lowlands	13,261	11	27.70	3,667	388	3	199	1,823	222	-
22 Pennine Dales Fringe	87,365	12	25.60	22,327	5,672	580	775	12,088	126	136
14 Tyne and Wear Lowlands	46,451	13	25.50	11,841	3,239	-	1,163	1,902	-	-
144 Quantock Hills	7,621	14	23.90	1,823	963	215	6	346	2	139
80 The Broads	56,253	15	23.10	12,990	1,312	44	1,305	7,161	-	-
141 Mendip Hills	30,322	16	22.50	6,811	2,927	132	39	2,002	301	390
16 Durham Coalfield Pennine Fringe	66,170	17.5	22.40	14,811	5,575	626	464	5,365	7	32
50 Derbyshire Peak Fringe and Lower Derwent	37,796	17.5	22.40	8,453	2,654	167	87	3,470	267	81
98 Clun and North West Herefordshire Hills	62,592	19	22.10	13,844	4,583	1,957	1,043	5,312	103	297
152 Cornish Killas	222,048	20	21.10	46,778	17,468	1,469	2,118	13,065	2,130	14
102 Teme Valley	19,312	21	20.50	3,960	2,114	80	216	1,335	2	-
64 Potteries and Churnet Valley	53,176	22	19.30	10,253	3,532	187	31	3,542	161	5
151 South Devon	121,122	23	19.20	23,313	8,681	713	760	5,808	1,232	37
54 Manchester Pennine Fringe	39,324	24	19.10	7,506	2,503	1	10	1,673	45	4
25 North York Moors and Cleveland Hills	165,979	25	18.70	31,033	12,096	4,586	461	8,924	1,003	6
99 Black Mountains and Golden Valley	25,992	26	18.10	4,714	1,861	120	193	2,133	24	212
9 Eden Valley	81,011	27	18.00	14,614	5,307	350	605	6,750	22	125
134 Dorset Downs and Cranborne Chase	116,942	28	17.80	20,817	5,960	644	3,790	6,961	2,259	148
82 Suffolk Coast and Heaths	82,129	29	16.90	13,893	1,757	598	2,741	3,750	-	-
122 High Weald	174,907	30	16.80	29,349	15,529	2,020	266	5,887	1,051	-
36 Southern Pennines	119,802	31	16.50	19,713	4,359	207	54	6,508	810	882
156 West Penwith	20,184	32	16.30	3,280	714	-	43	1,184	169	-
148 Devon Redlands	97,453	33	16.10	15,668	4,394	1,143	571	5,374	219	-
153 Bodmin Moor	28,578	34	15.70	4,484	1,288	135	7	1,538	-	840
51 Dark Peak	86,667	35	15.30	13,292	4,179	1,097	23	3,293	629	1,151
124 Pevensey Levels	9,639	36.5	14.60	1,404	24	-	15	596	-	-
120 Wealden Greensand	145,830	36.5	14.60	21,240	11,088	2,208	395	3,183	604	-
150 Dartmoor	87,437	38	14.40	12,562	6,483	880	13	2,357	1	2,055
52 White Peak	52,898	39	14.20	7,523	1,870	195	4	1,442	3,046	86
127 Isle of Wight	38,043	40	13.80	5,243	1,527	107	399	1,453	660	-
29 Howardian Hills	24,026	41	13.50	3,235	1,809	204	166	724	69	-
155 Carnmenellis	14,321	42.5	13.40	1,925	838	4	24	527	-	-
130 Hampshire Downs	149,017	42.5	13.40	19,999	8,425	679	3,142	2,913	2,871	-
65 Shropshire Hills	108,061	44	13.10	14,203	5,584	540	700	5,713	145	648
20 Morecambe Bay Limestones	39,992	45	13.00	5,199	2,313	6	46	1,973	15	122
38 Nottinghams, Derbys & Yorks Coalfield	169,872	46	12.90	21,841	6,545	120	697	4,765	1	-
19 South Cumbria Low Fells	69,184	47	12.80	8,865	3,980	27	30	3,160	-	274
63 Oswestry Uplands	9,987	48	12.70	1,265	468	60	5	517	-	116
145 Exmoor	130,426	49.5	12.50	16,264	8,560	1,167	59	3,754	1	767
133 Blackmore Vale and Vale of Wardour	78,473	49.5	12.50	9,782	3,554	1,185	525	3,384	457	26
2 Northumberland Sandstone Hills	72,746	51.5	12.30	8,927	4,091	408	115	3,496	-	183
13 South East Northumberland Coastal Plain	43,740	51.5	12.30	5,389	1,621	-	882	980	-	-

53 South West Peak	42,600	53	12.20	5,208	1,656	164	8	1,934	172	363
131 New Forest	73,821	54	11.90	8,777	6,231	1,074	-	221	-	-
132 Salisbury Plain and West Wiltshire Downs	122,427	55	11.70	14,311	4,478	527	1,003	1,562	5,587	-
73 Charnwood	17,475	56	10.90	1,908	874	49	10	450	2	1
104 South Herefordshire and Over Severn	51,185	57	10.50	5,394	2,676	293	261	1,713	59	50
66 Mid Severn Sandstone Plateau	88,869	58	10.30	9,189	5,623	496	159	1,140	1	-
15 Durham Magnesian Limestone Plateau	45,292	59	10.10	4,552	1,311	-	498	1,077	19	-
12 Mid Northumberland	63,772	60	9.90	6,286	2,375	19	361	2,922	-	2
17 Orton Fells	29,301	62	9.60	2,815	706	92	8	1,190	341	194
101 Herefordshire Plateau	34,660	62	9.60	3,338	1,463	47	237	1,451	3	2
135 Dorset Heaths	61,709	62	9.60	5,909	1,718	727	35	1,383	29	-
149 The Culm	283,137	64	9.40	26,567	13,511	1,410	276	9,143	49	117
8 Cumbria High Fells	199,127	65	8.60	17,068	6,108	454	75	5,467	22	2,136
7 West Cumbria Coastal Plain	49,318	66	8.30	4,080	1,154	1	91	1,525	-	52
107 Cotswolds	288,389	67	8.20	23,625	11,270	334	626	6,660	2,144	-
33 Bowland Fringe and Pendle Hill	74,141	69	8.00	5,918	2,149	6	33	3,207	-	89
118 Bristol, Avon Valleys and Ridges	84,317	69	8.00	6,742	2,526	59	29	1,446	43	-
137 Isle of Porland	1,125	69	8.00	90	5	-	-	-	9	-
67 Cannock Chase and Cank Wood	72,845	71	7.60	5,552	2,346	738	44	674	-	-
31 Morecambe Coast and Lune Estuary	13,220	72	7.40	978	96	-	19	393	-	-
21 Yorkshire Dales	240,157	73	7.30	17,464	3,790	664	45	8,427	2,214	416
3 Cheviot Fringe	51,628	74.5	7.00	3,627	1,122	5	274	2,006	-	7
77 North Norfolk Coast	6,243	74.5	7.00	437	4	-	7	112	-	-
140 Yeovil Scarplands	78,636	76	6.80	5,355	1,574	198	180	2,595	10	14
5 Border Moors and Forests	127,245	77	6.60	8,343	2,403	1,583	21	3,013	24	527
35 Lancashire Valleys	55,463	78.5	6.50	3,580	1,207	5	6	1,386	30	3
146 Vale of Taunton and Quantock Fringes	48,432	78.5	6.50	3,148	996	77	124	1,149	10	-
24 Vale of Mowbray	60,676	81	6.40	3,872	715	17	455	1,982	33	-
57 Sefton Coast	8,995	81	6.40	574	88	-	27	50	-	-
123 Romney Marshes	36,674	81	6.40	2,360	76	-	310	1,145	143	-
10 North Pennines	214,717	83.5	5.50	11,858	3,945	468	61	4,523	1,324	506
113 North Kent Plain	84,815	83.5	5.50	4,655	1,565	49	386	638	252	-
23 Tees Lowlands	102,264	85	5.30	5,458	1,256	7	774	1,432	1	-
110 Chilterns	164,181	86	5.10	8,319	5,684	187	169	1,496	131	-
6 Solway Basin	98,410	87	4.90	4,807	1,654	1	186	1,799	-	-
79 North East Norfolk and Flegg	24,636	88.5	4.60	1,121	77	-	354	165	-	-
100 Herefordshire Lowlands	88,742	88.5	4.60	4,089	1,954	120	224	1,414	22	3
142 Somerset Levels and Moors	65,841	90	4.50	2,967	279	-	42	916	11	-
55 Manchester Conurbation	34,248	91	4.40	1,501	458	-	3	105	-	-
68 Needwood and South Derbyshire Claylands	81,600	92	4.20	3,427	903	20	36	1,753	-	-
27 Yorkshire Wolds	111,479	93	4.10	4,566	1,843	60	859	424	1,080	-
85 The Brecks	101,920	94	3.90	3,934	1,504	299	68	1,290	31	47
116 Berkshire and Marlborough Downs	111,069	95	3.70	4,147	2,118	91	262	730	523	-
41 Humber Estuary	27,961	96	3.50	988	40	-	140	114	-	-
59 Wirral	16,526	97	3.40	564	11	-	-	86	-	-
71 Leicestershire and South Derbyshire Coalfield	20,486	98	3.00	623	367	8	2	110	-	-
4 Cheviots	36,514	99	2.90	1,043	482	31	17	353	-	127
70 Melbourne Parklands	15,056	100	2.80	425	182	-	6	65	-	-
18 Howgill Fells	10,367	103	2.70	277	47	4	-	152	-	47
30 Southern Magnesian Limestone	136,856	103	2.70	3,739	984	24	227	1,006	1	-
157 The Lizard	14,741	103	2.70	405	243	-	3	20	29	-
126 South Coast Plain	52,277	103	2.70	1,408	198	12	64	244	-	-
143 Mid Somerset Hills	42,121	103	2.70	1,127	529	9	63	381	4	-
58 Merseyside Conurbation	28,697	107	2.50	722	7	-	6	15	-	-
128 South Hampshire Lowlands	38,662	107	2.50	976	639	67	7	83	3	-
1 North Northumberland Coastal Plain	37,696	109	2.40	886	346	-	86	290	-	-
69 Trent Valley Washlands	39,404	109	2.40	958	155	-	16	242	-	-
106 Severn and Avon Vales	210,486	109	2.40	5,031	1,007	29	169	1,743	89	-
28 Vale of York	102,147	112	2.20	2,273	285	11	157	366	7	-
81 Greater Thames Estuary	83,665	112	2.20	1,812	90	1	272	519	8	-
121 Low Weald	182,470	112	2.20	4,076	2,214	290	69	751	107	-
34 Bowland Fells	37,420	114	2.00	746	351	32	-	227	-	107

45 Northern Lincolnshire Edge with Coversands	50,084	116	1.90	970	309	13	178	63	1	-
111 Northern Thames Basin	251,031	116	1.90	4,826	1,230	9	489	718	3	4
129 Thames Basin Heaths	118,600	117	1.80	2,109	1,345	141	53	236	143	-
86 South Suffolk and North Essex Clayland	328,998	118	1.70	5,630	1,480	4	643	1,830	1	7
83 South Norfolk & High Suffolk Claylands	214,428	120	1.60	3,350	781	3	309	1,588	-	20
87 East Anglian Chalk	83,890	120	1.60	1,310	214	-	133	446	8	-
117 Avon Vales	64,334	120	1.60	1,061	434	5	24	187	245	-
26 Vale of Pickering	43,108	124	1.40	589	105	-	72	240	-	-
44 Central Lincolnshire Vale	81,932	124	1.40	1,164	94	-	461	206	-	-
48 Trent and Belvoir Vales	177,708	124	1.40	2,400	296	-	872	268	-	-
78 Central North Norfolk	72,008	124	1.40	995	556	98	64	161	-	-
62 Cheshire Sandstone Ridge	22,057	126	1.30	286	205	3	6	32	-	-
61 Shropshire, Cheshire and Staffordshire Plain	366,505	127	1.20	4,221	1,087	7	39	1,545	-	-
42 Lincolnshire Coast and Marshes	88,224	128	1.00	873	6	-	505	60	-	-
32 Lancashire and Amounderness Plain	98,658	129	0.90	879	118	-	66	165	-	-
39 Humberhead Levels	171,910	131	0.80	1,415	189	49	199	37	-	-
49 Sherwood	53,492	131	0.80	426	215	15	7	42	-	-
47 Southern Lincolnshire Edge	57,068	133	0.70	428	52	-	15	83	-	-
97 Arden	143,532	133	0.70	989	376	3	1	131	-	-
56 Lancashire Coal Measures	40,613	135	0.60	247	117	-	2	43	-	-
109 Midvale Ridge	44,532	135	0.60	264	58	-	-	31	-	-
94 Leicestershire Vales	71,842	136	0.50	376	64	-	3	74	-	-
46 The Fens	382,711	139	0.40	1,511	78	5	438	344	-	-
84 Mid Norfolk	90,862	139	0.40	389	139	-	21	116	-	-
88 Bedfordshire and Cambridgeshire Claylands	260,682	139	0.40	1,087	157	2	8	224	-	-
89 Northamptonshire Vales	90,440	139	0.40	369	40	-	1	75	3	-
108 Upper Thames Clay Vales	189,134	139	0.40	815	124	2	5	99	-	-
40 Holderness	87,318	143	0.30	305	25	-	110	54	4	-
60 Mersey Valley	44,750	143	0.30	133	9	-	2	6	-	-
76 North West Norfolk	80,140	143	0.30	249	58	-	2	141	-	-
72 Mease/Sence Lowlands	32,377	146	0.20	68	15	-	-	30	-	-
74 Leicestershire and Nottinghamshire Wolds	64,110	146	0.20	144	32	-	-	59	-	-
43 Lincolnshire Wolds	84,517	149	0.10	50	24	-	-	5	5	-
92 Rockingham Forest	51,029	149	0.10	43	8	-	-	15	1	-
93 High Leicestershire	56,910	149	0.10	31	12	-	-	12	-	-
112 Inner London	33,023	149	0.10	31	2	-	-	8	-	-
114 Thames Basin Lowlands	32,797	149	0.10	32	22	1	-	1	-	-
75 Kesteven Uplands	69,038	154	0.00	30	14	-	-	-	-	-
91 Yardley-Whittlewood Ridge	33,797	154	0.00	2	1	-	-	-	-	-
95 Northamptonshire Uplands	101,210	154	0.00	1	-	-	-	-	-	-
96 Dunsmore and Feldon	70,648	154	0.00	32	5	-	-	-	-	-
115 Thames Valley	86,107	154	0.00	14	-	-	-	2	-	-
158 Isles of Scilly	1,636	157	0.00	-	-	-	-	-	-	-
159 Lundy	451	158	0.00	-	-	-	-	-	-	-
90 Bedfordshire Greensand Ridge	27,350	159	0.00	-	-	-	-	-	-	-
