# Supplementary figures and images for: Designing a Climate Change Resilient Landscape Connectivity Network From a Multi‐Species Perspective
Source: Ecol Evol. 2025 Sep 18;15(9):e71956. doi: 10.1002/ece3.71956 (PMC12446580; doi:10.1002/ece3.71956)

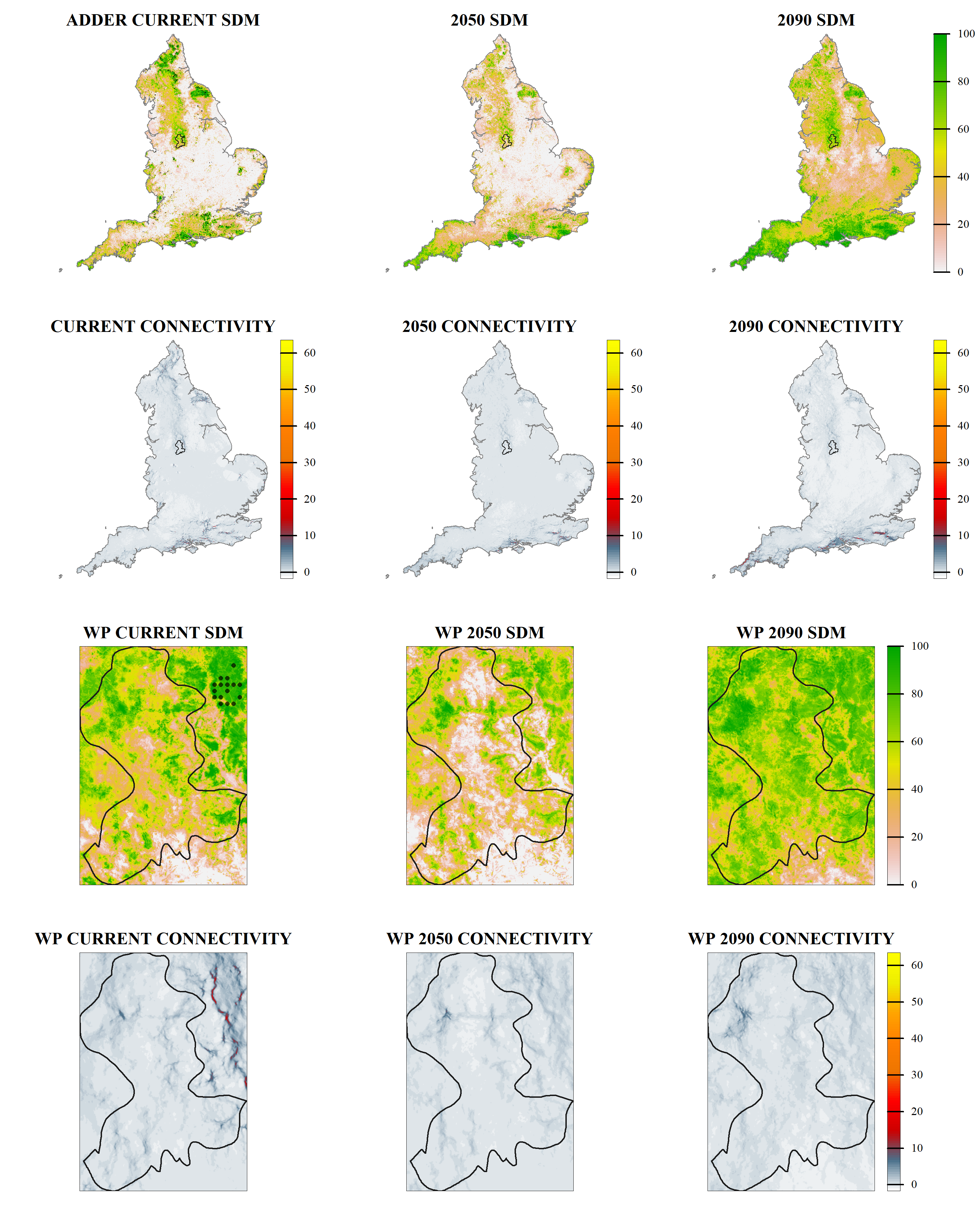

Supplement: Supplementary file 2 — Data S1: ece371956‐sup‐0002‐Supinfo.zip. [file ECE3-15-e71956-s001.zip › SUPPORTING.INFORMATION/CONNECTIVITY.PLOTS.SPECIES/ADDER.tif]

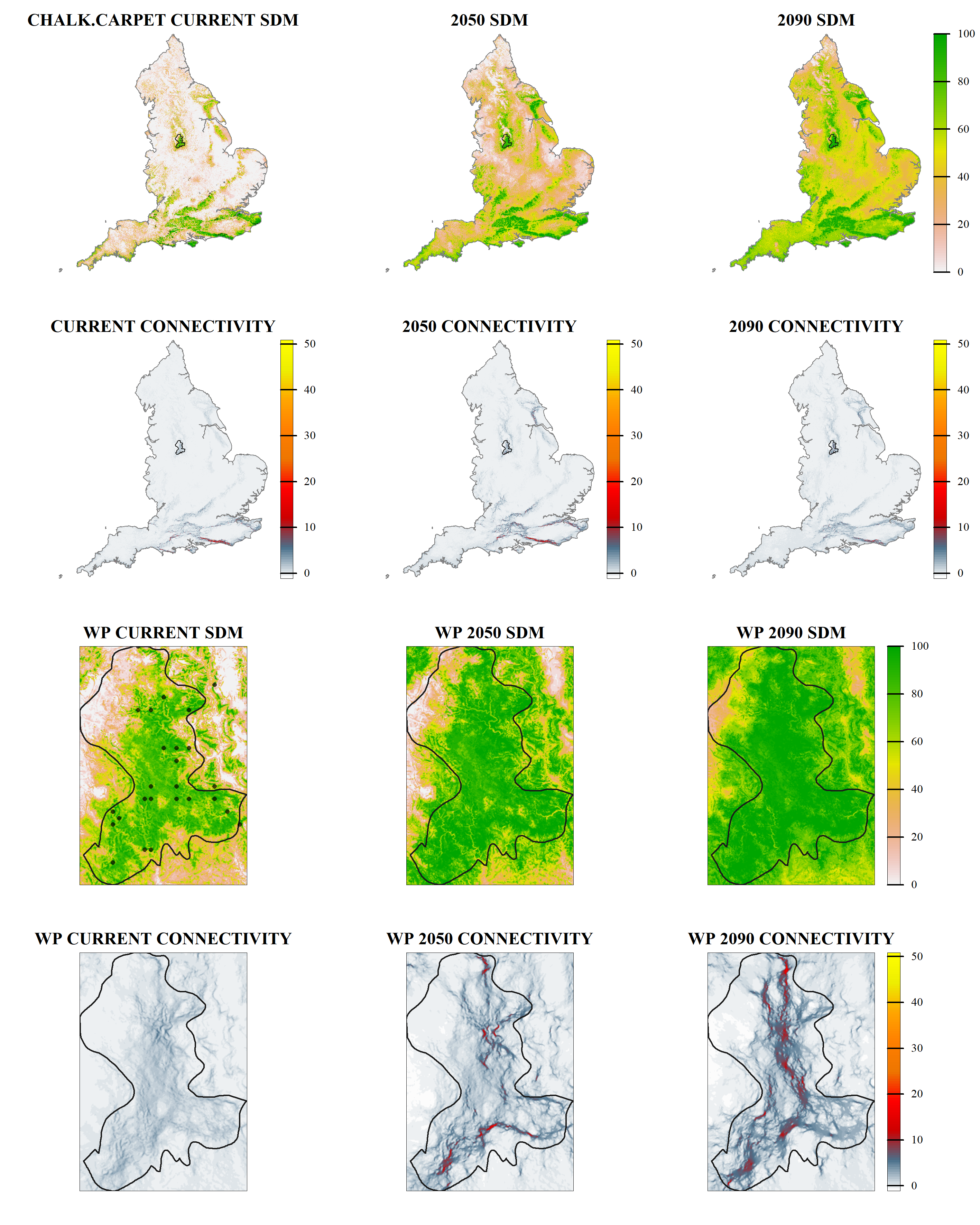

Supplement: Supplementary file 2 — Data S1: ece371956‐sup‐0002‐Supinfo.zip. [file ECE3-15-e71956-s001.zip › SUPPORTING.INFORMATION/CONNECTIVITY.PLOTS.SPECIES/CHALK.CARPET.tif]

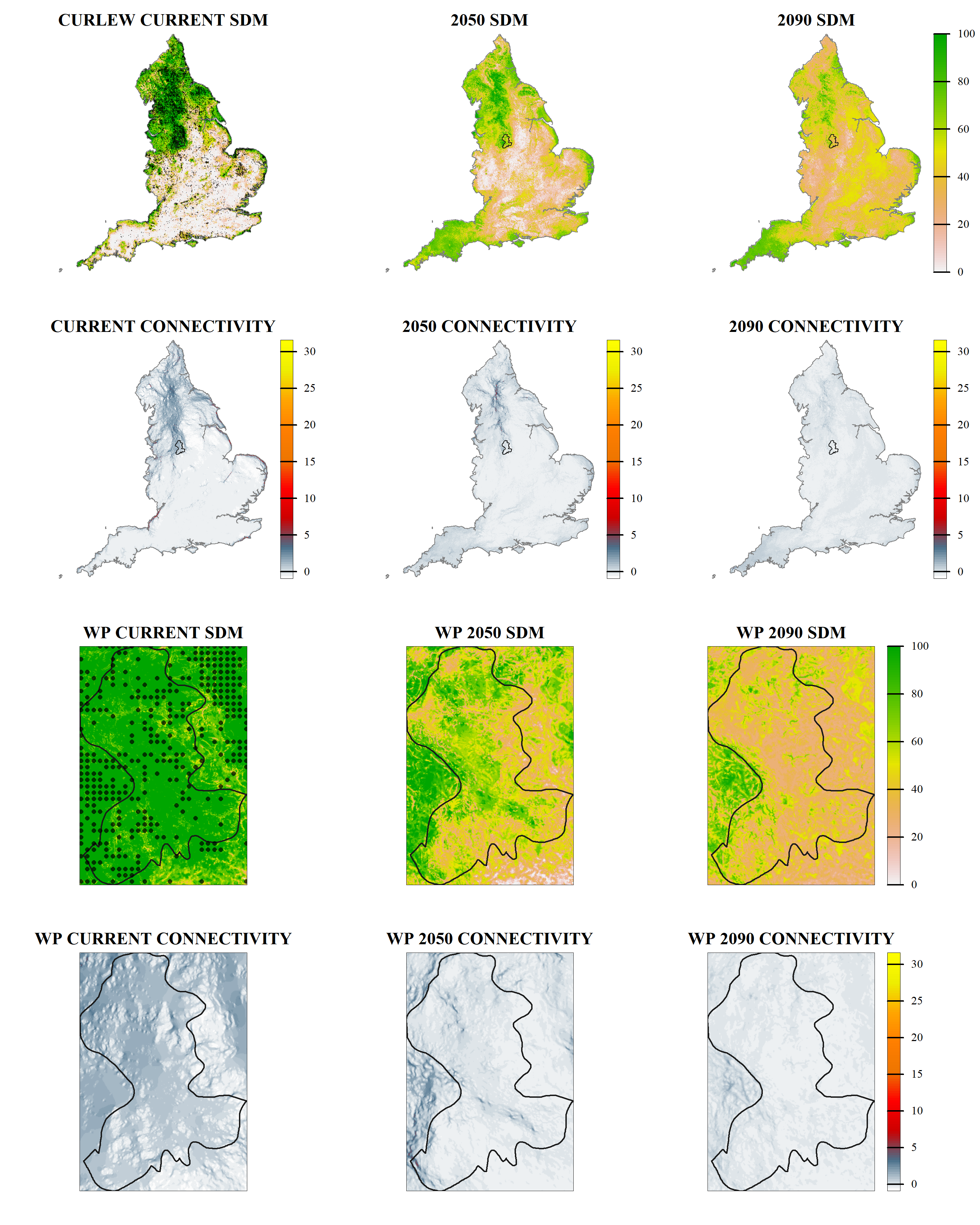

Supplement: Supplementary file 2 — Data S1: ece371956‐sup‐0002‐Supinfo.zip. [file ECE3-15-e71956-s001.zip › SUPPORTING.INFORMATION/CONNECTIVITY.PLOTS.SPECIES/CURLEW.tif]

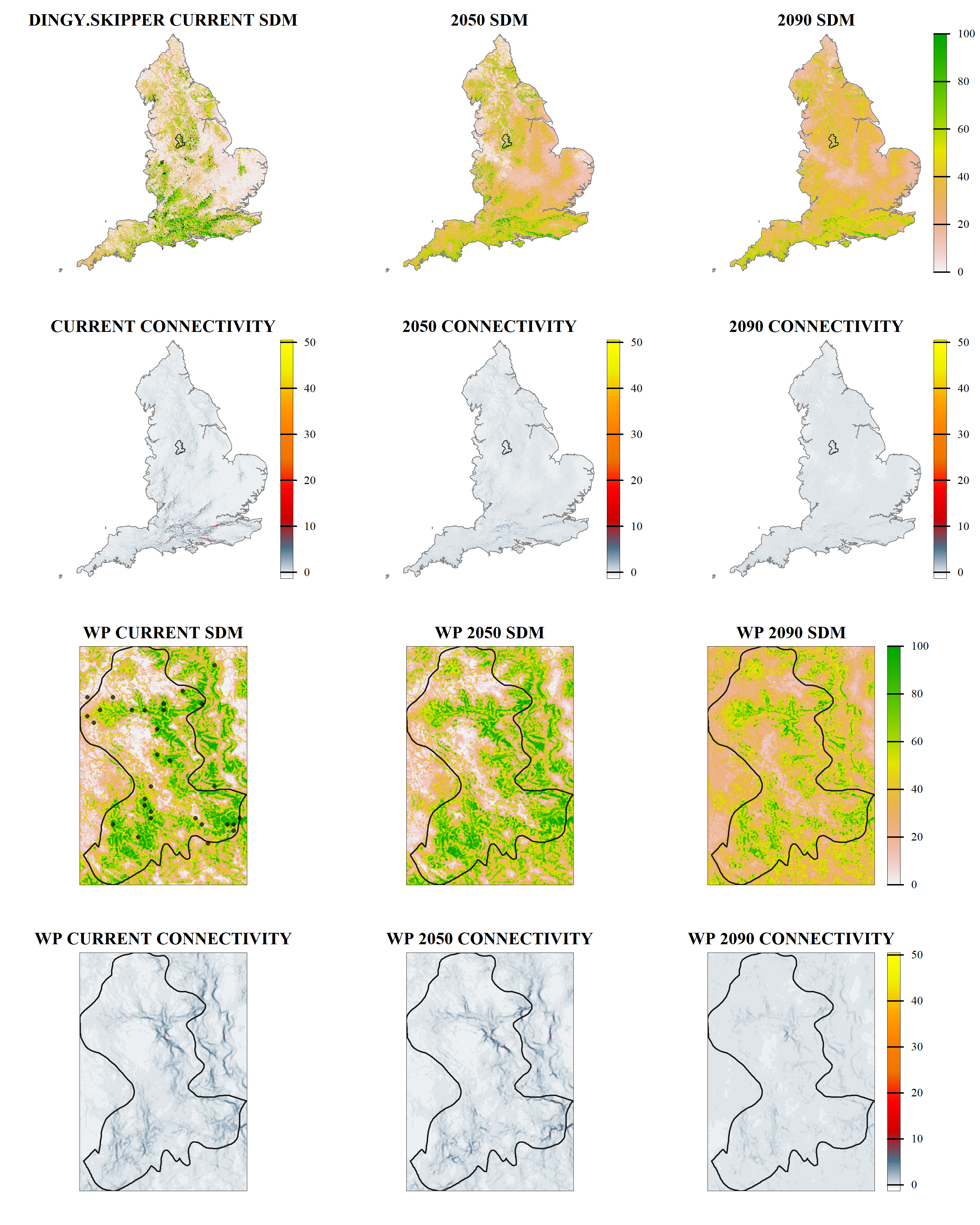

Supplement: Supplementary file 2 — Data S1: ece371956‐sup‐0002‐Supinfo.zip. [file ECE3-15-e71956-s001.zip › SUPPORTING.INFORMATION/CONNECTIVITY.PLOTS.SPECIES/DINGY.SKIPPER.tif]

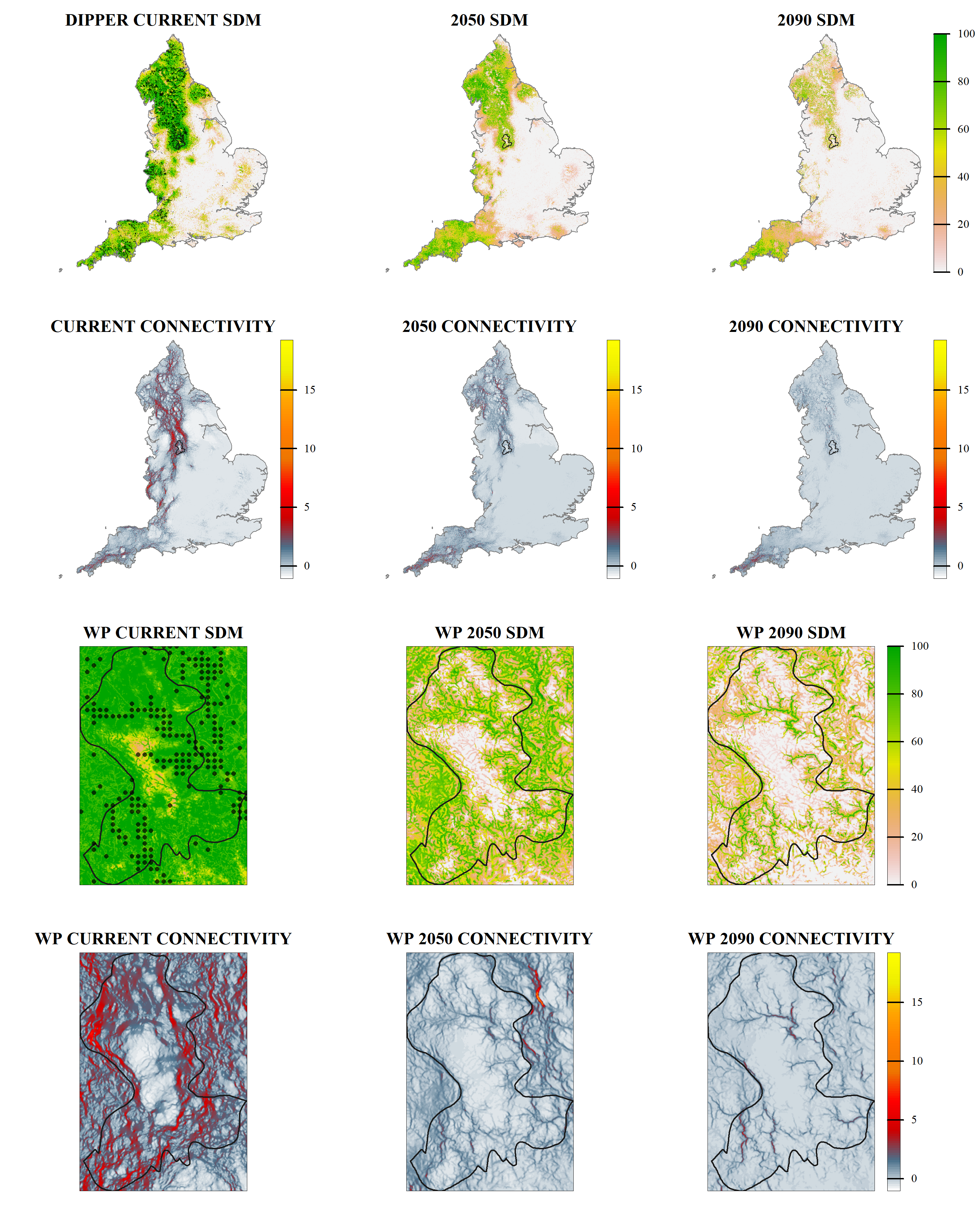

Supplement: Supplementary file 2 — Data S1: ece371956‐sup‐0002‐Supinfo.zip. [file ECE3-15-e71956-s001.zip › SUPPORTING.INFORMATION/CONNECTIVITY.PLOTS.SPECIES/DIPPER.tif]

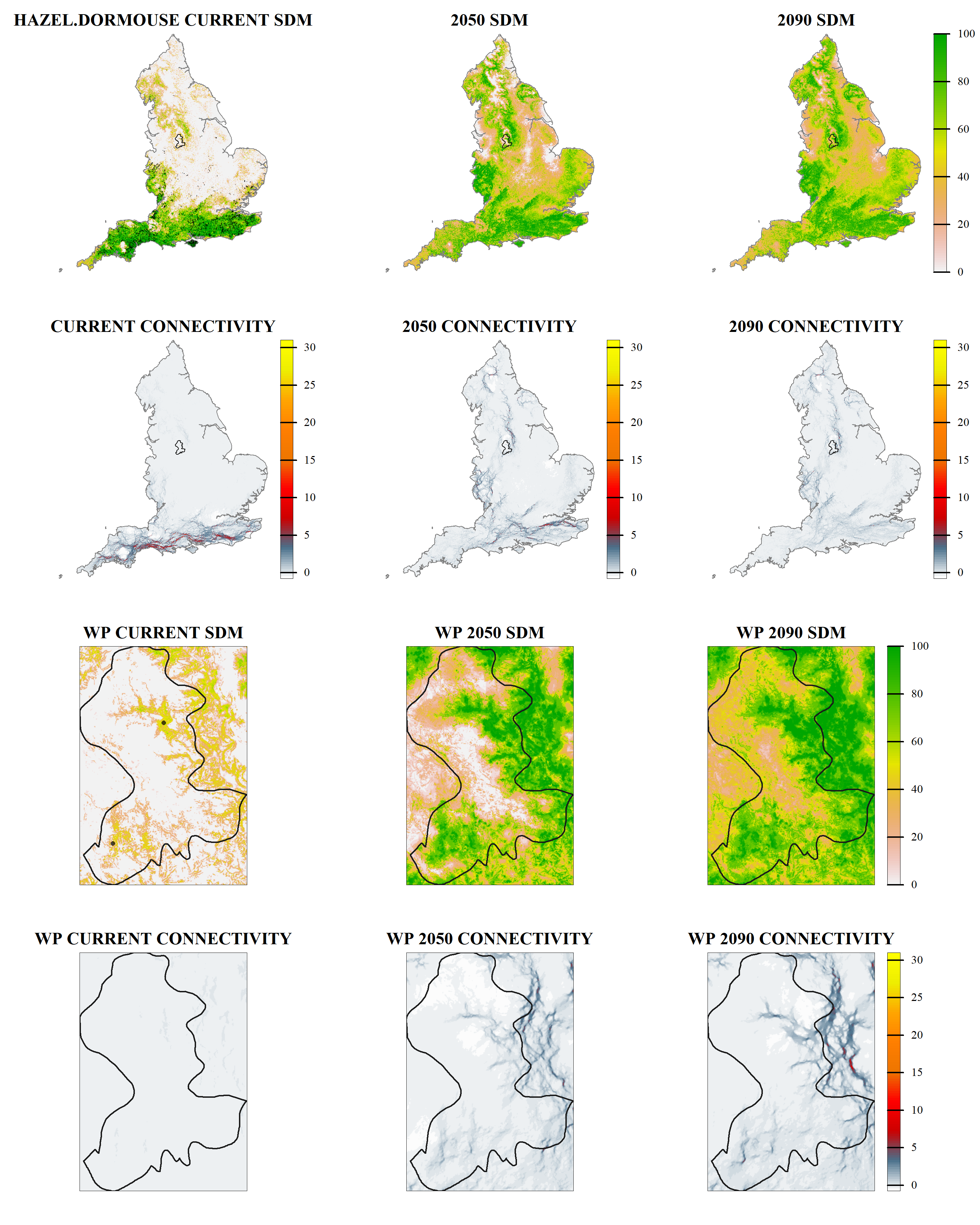

Supplement: Supplementary file 2 — Data S1: ece371956‐sup‐0002‐Supinfo.zip. [file ECE3-15-e71956-s001.zip › SUPPORTING.INFORMATION/CONNECTIVITY.PLOTS.SPECIES/HAZEL.DORMOUSE.tif]

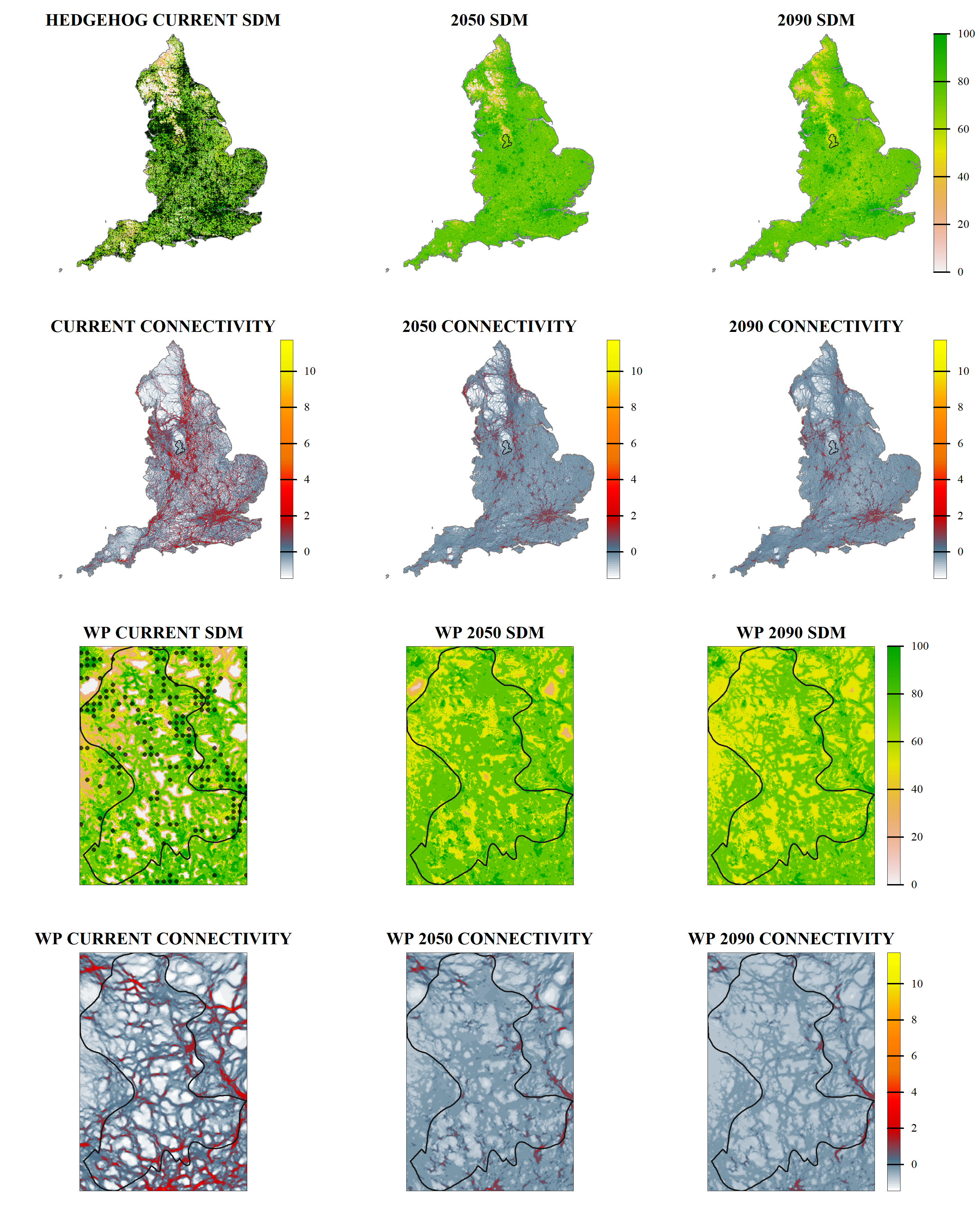

Supplement: Supplementary file 2 — Data S1: ece371956‐sup‐0002‐Supinfo.zip. [file ECE3-15-e71956-s001.zip › SUPPORTING.INFORMATION/CONNECTIVITY.PLOTS.SPECIES/HEDGEHOG.tif]

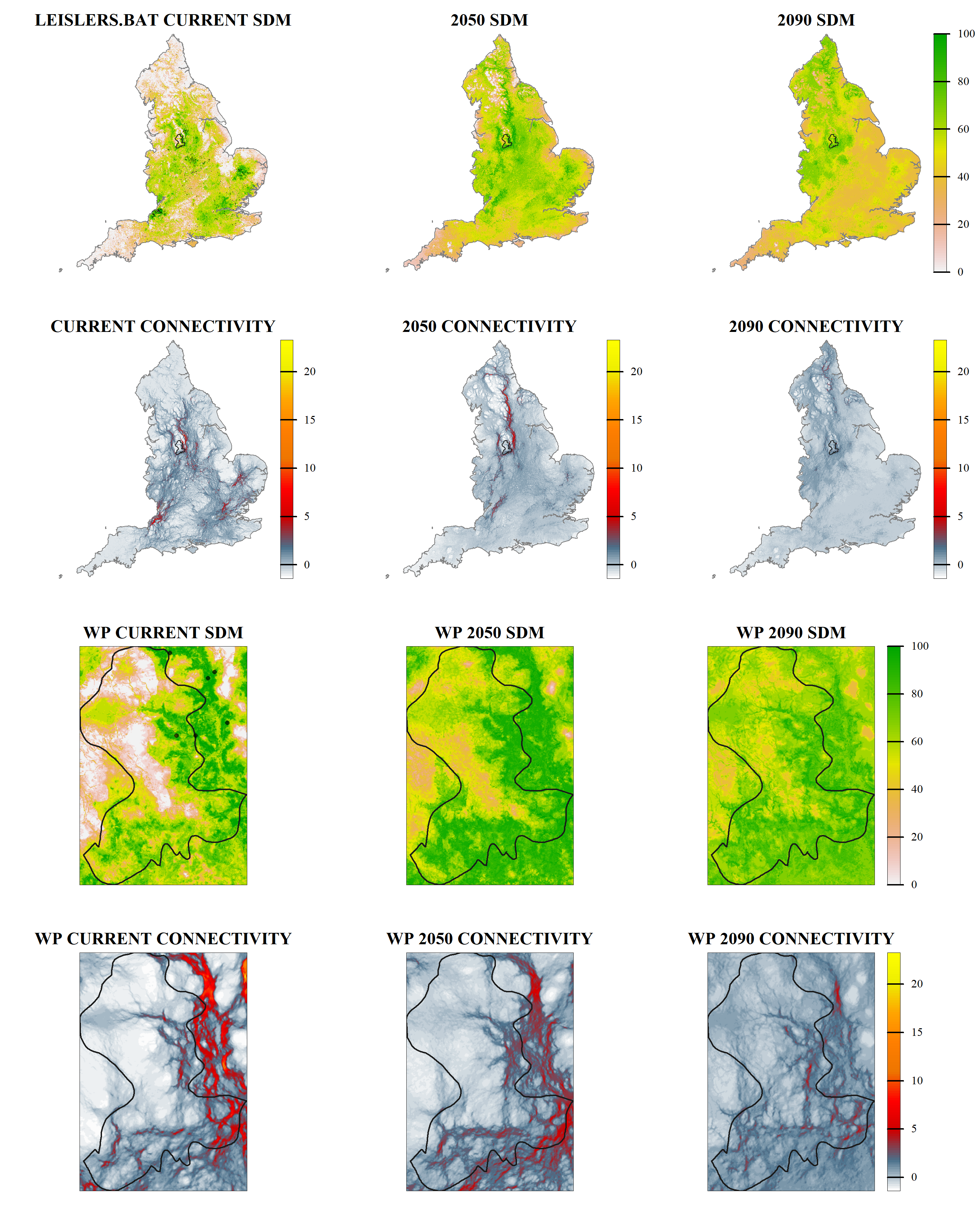

Supplement: Supplementary file 2 — Data S1: ece371956‐sup‐0002‐Supinfo.zip. [file ECE3-15-e71956-s001.zip › SUPPORTING.INFORMATION/CONNECTIVITY.PLOTS.SPECIES/LEISLERS.BAT.tif]

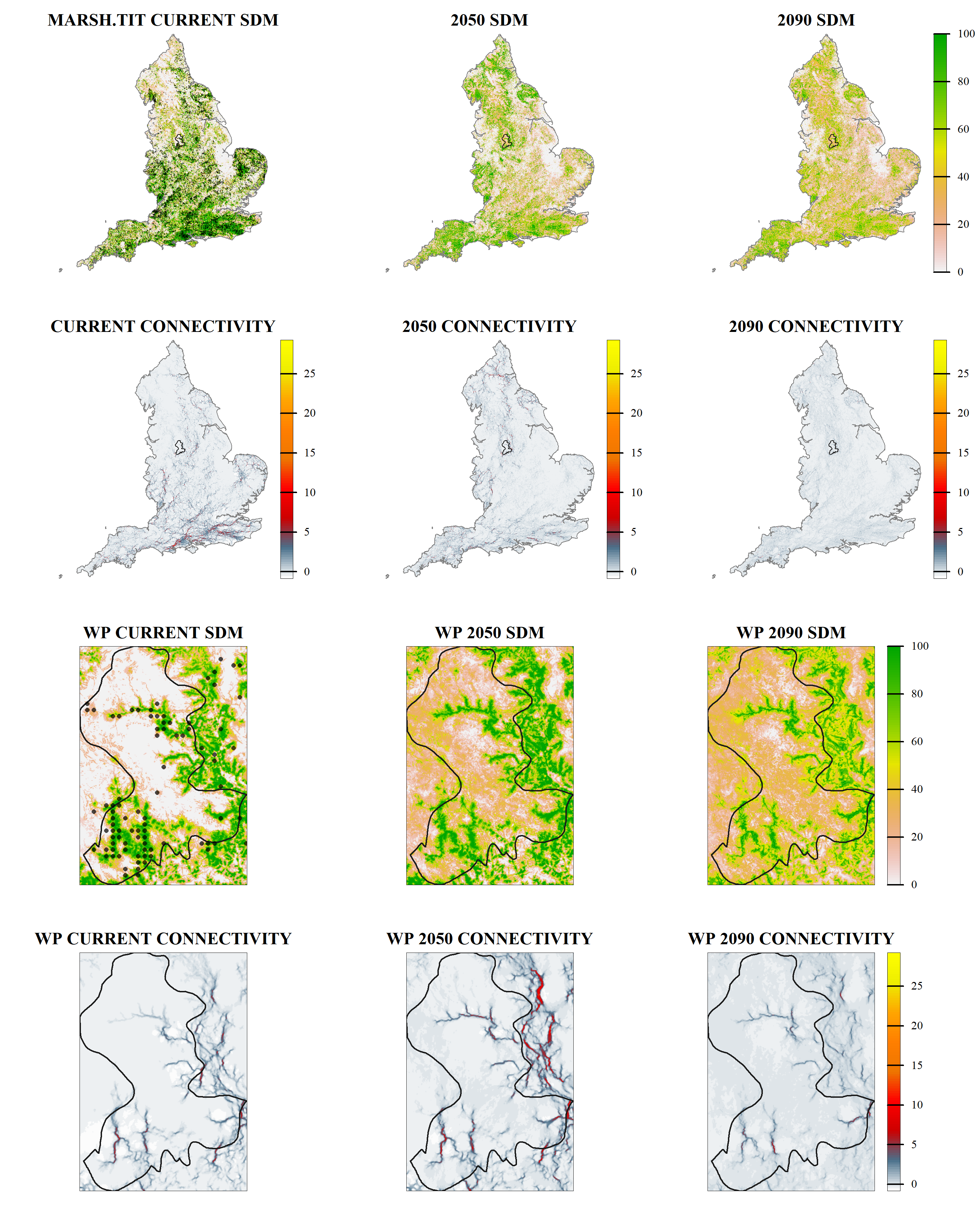

Supplement: Supplementary file 2 — Data S1: ece371956‐sup‐0002‐Supinfo.zip. [file ECE3-15-e71956-s001.zip › SUPPORTING.INFORMATION/CONNECTIVITY.PLOTS.SPECIES/MARSH.TIT.tif]

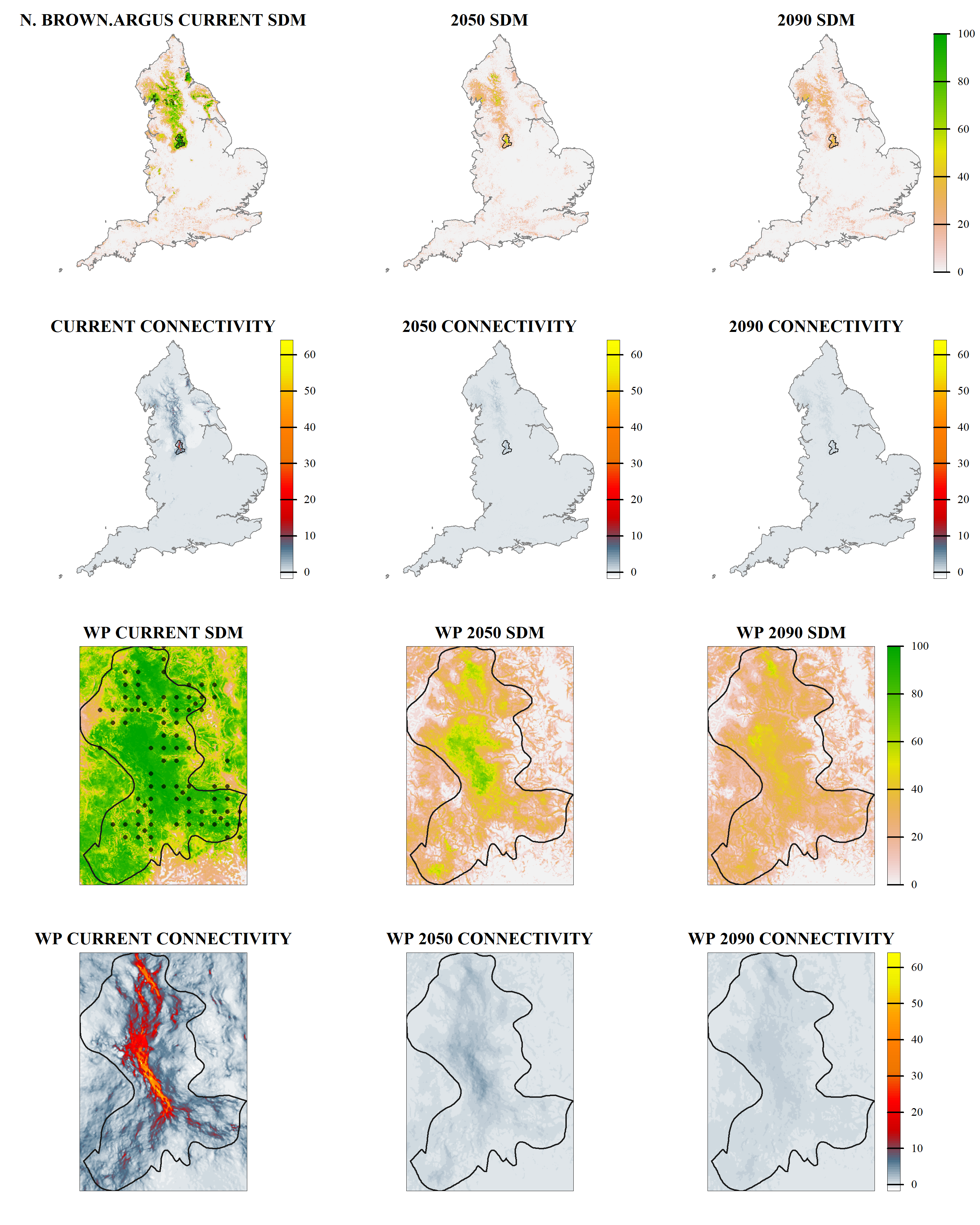

Supplement: Supplementary file 2 — Data S1: ece371956‐sup‐0002‐Supinfo.zip. [file ECE3-15-e71956-s001.zip › SUPPORTING.INFORMATION/CONNECTIVITY.PLOTS.SPECIES/N. BROWN.ARGUS.tif]

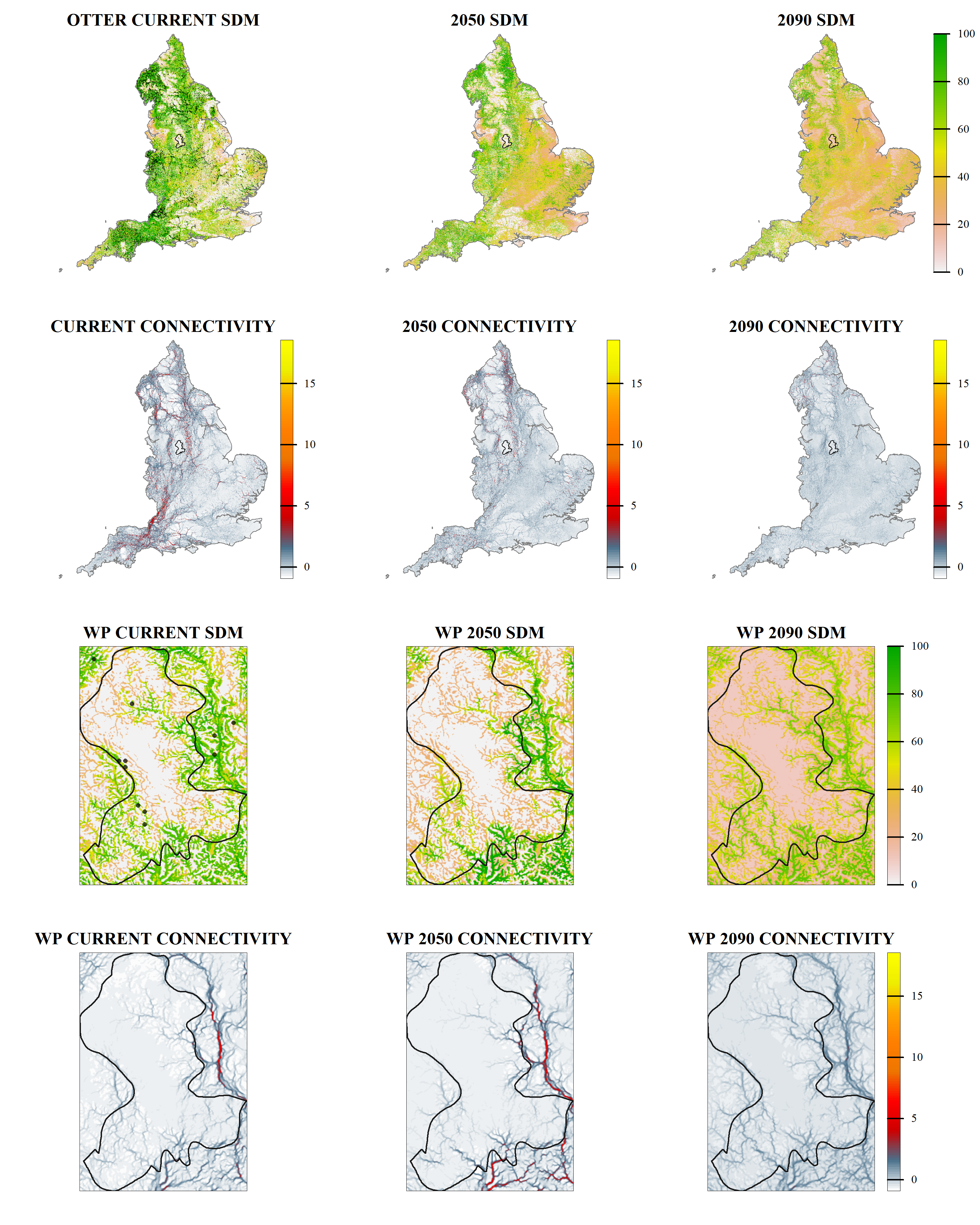

Supplement: Supplementary file 2 — Data S1: ece371956‐sup‐0002‐Supinfo.zip. [file ECE3-15-e71956-s001.zip › SUPPORTING.INFORMATION/CONNECTIVITY.PLOTS.SPECIES/OTTER.tif]

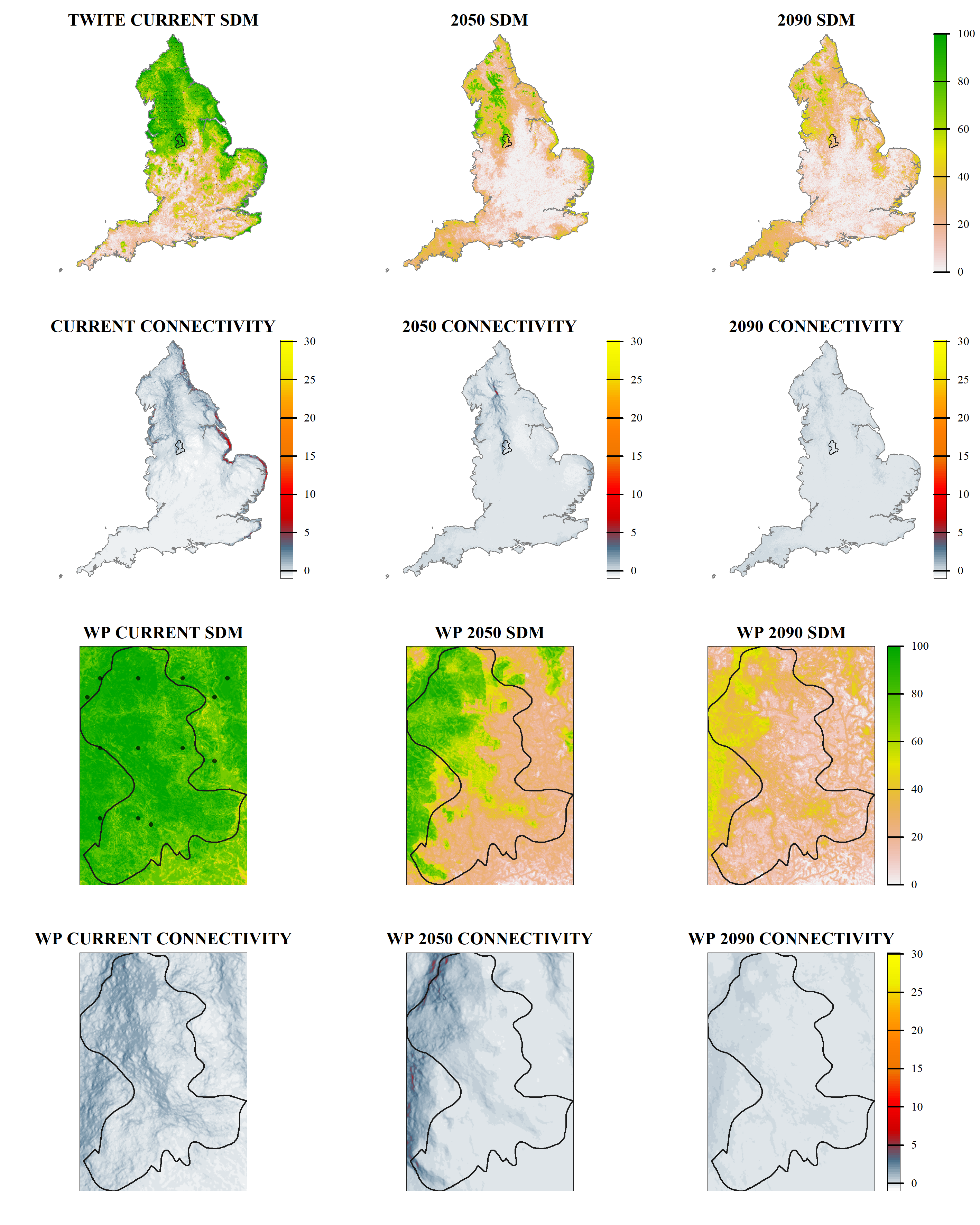

Supplement: Supplementary file 2 — Data S1: ece371956‐sup‐0002‐Supinfo.zip. [file ECE3-15-e71956-s001.zip › SUPPORTING.INFORMATION/CONNECTIVITY.PLOTS.SPECIES/TWITE.tif]

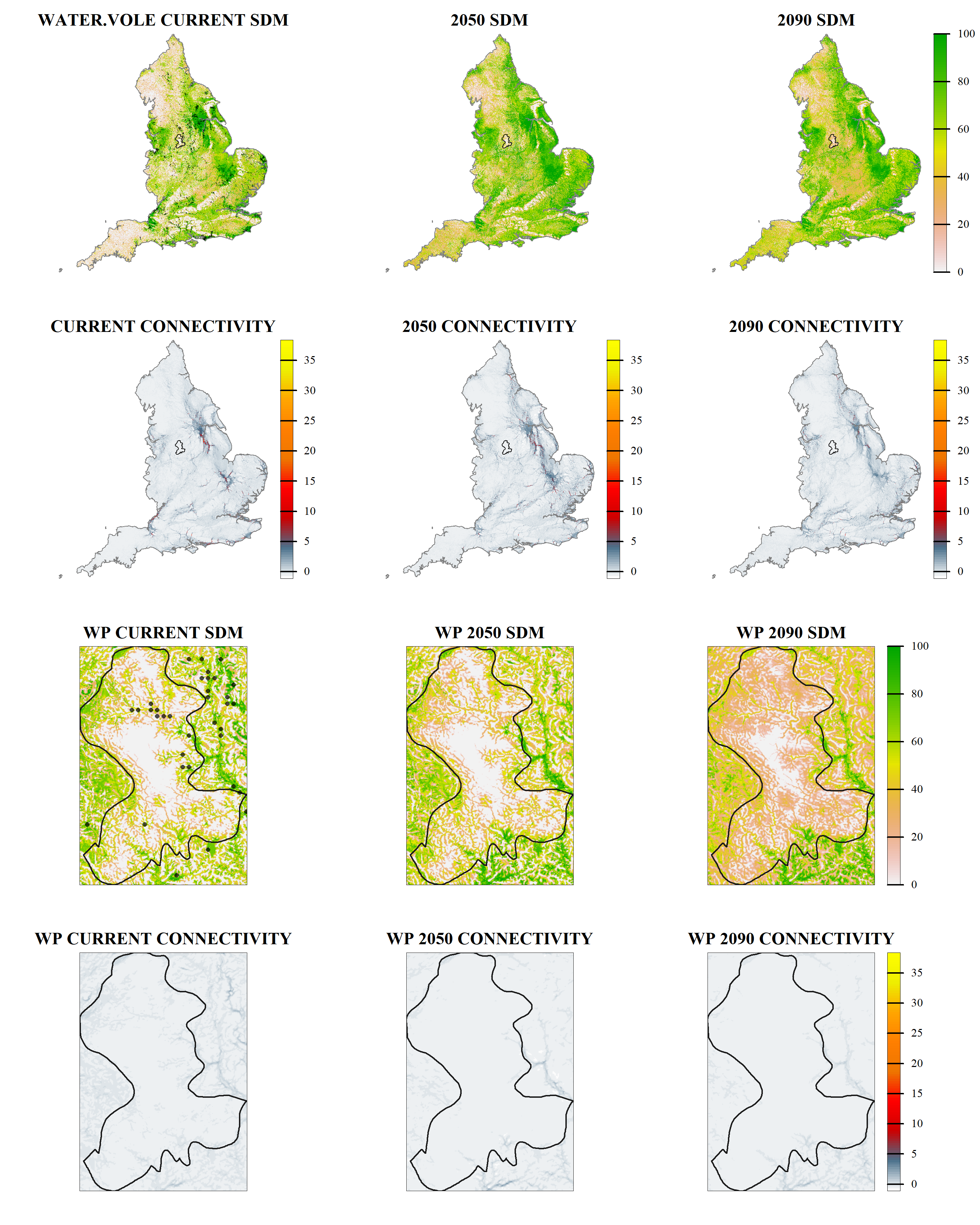

Supplement: Supplementary file 2 — Data S1: ece371956‐sup‐0002‐Supinfo.zip. [file ECE3-15-e71956-s001.zip › SUPPORTING.INFORMATION/CONNECTIVITY.PLOTS.SPECIES/WATER.VOLE.tif]

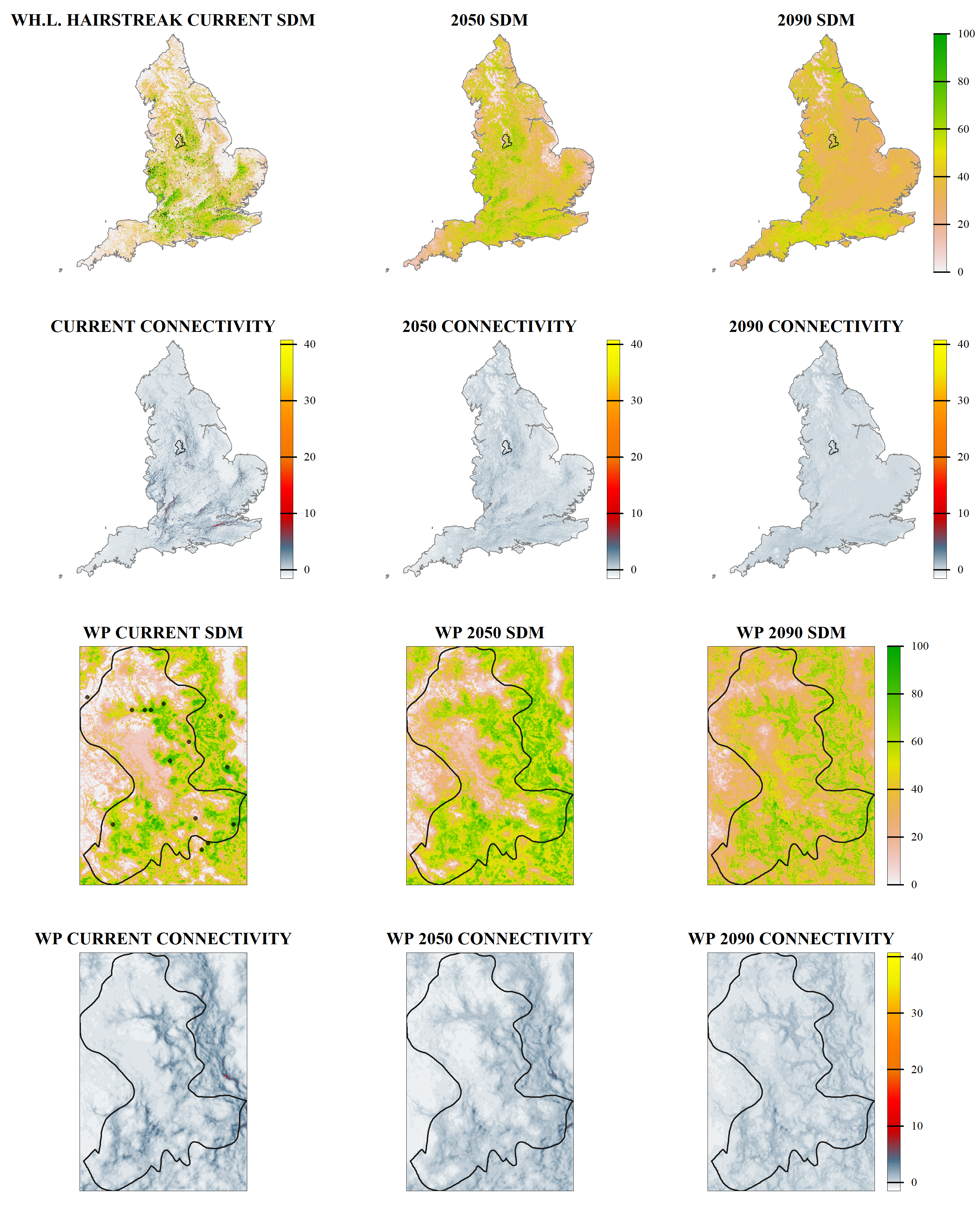

Supplement: Supplementary file 2 — Data S1: ece371956‐sup‐0002‐Supinfo.zip. [file ECE3-15-e71956-s001.zip › SUPPORTING.INFORMATION/CONNECTIVITY.PLOTS.SPECIES/WH.L. HAIRSTREAK.tif]

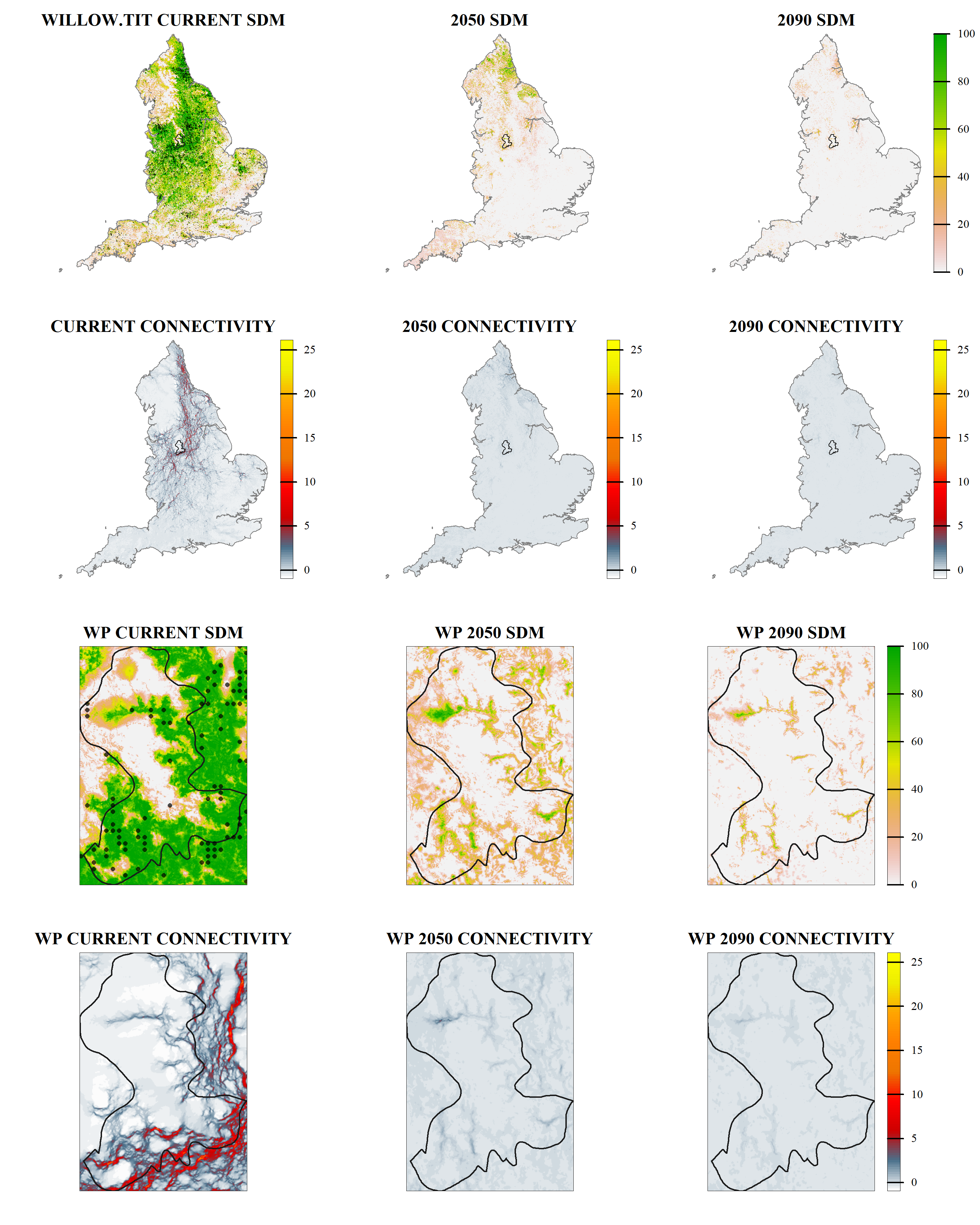

Supplement: Supplementary file 2 — Data S1: ece371956‐sup‐0002‐Supinfo.zip. [file ECE3-15-e71956-s001.zip › SUPPORTING.INFORMATION/CONNECTIVITY.PLOTS.SPECIES/WILLOW.TIT.tif]

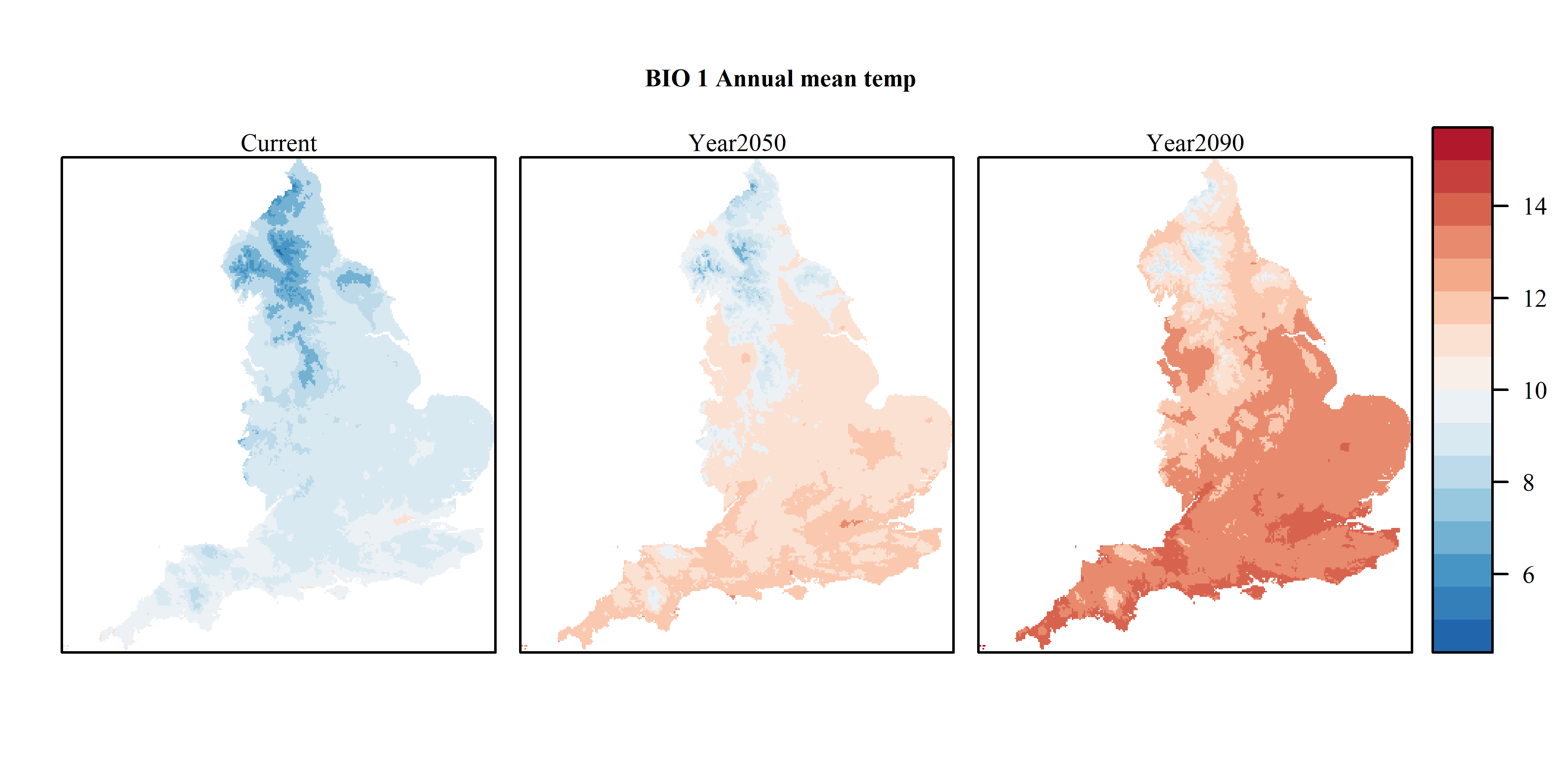

Supplement: Supplementary file 2 — Data S1: ece371956‐sup‐0002‐Supinfo.zip. [file ECE3-15-e71956-s001.zip › SUPPORTING.INFORMATION/ENVIRONMENTAL.VARIABLE.PREDICTOR.PLOTS/BIO.1.tif]

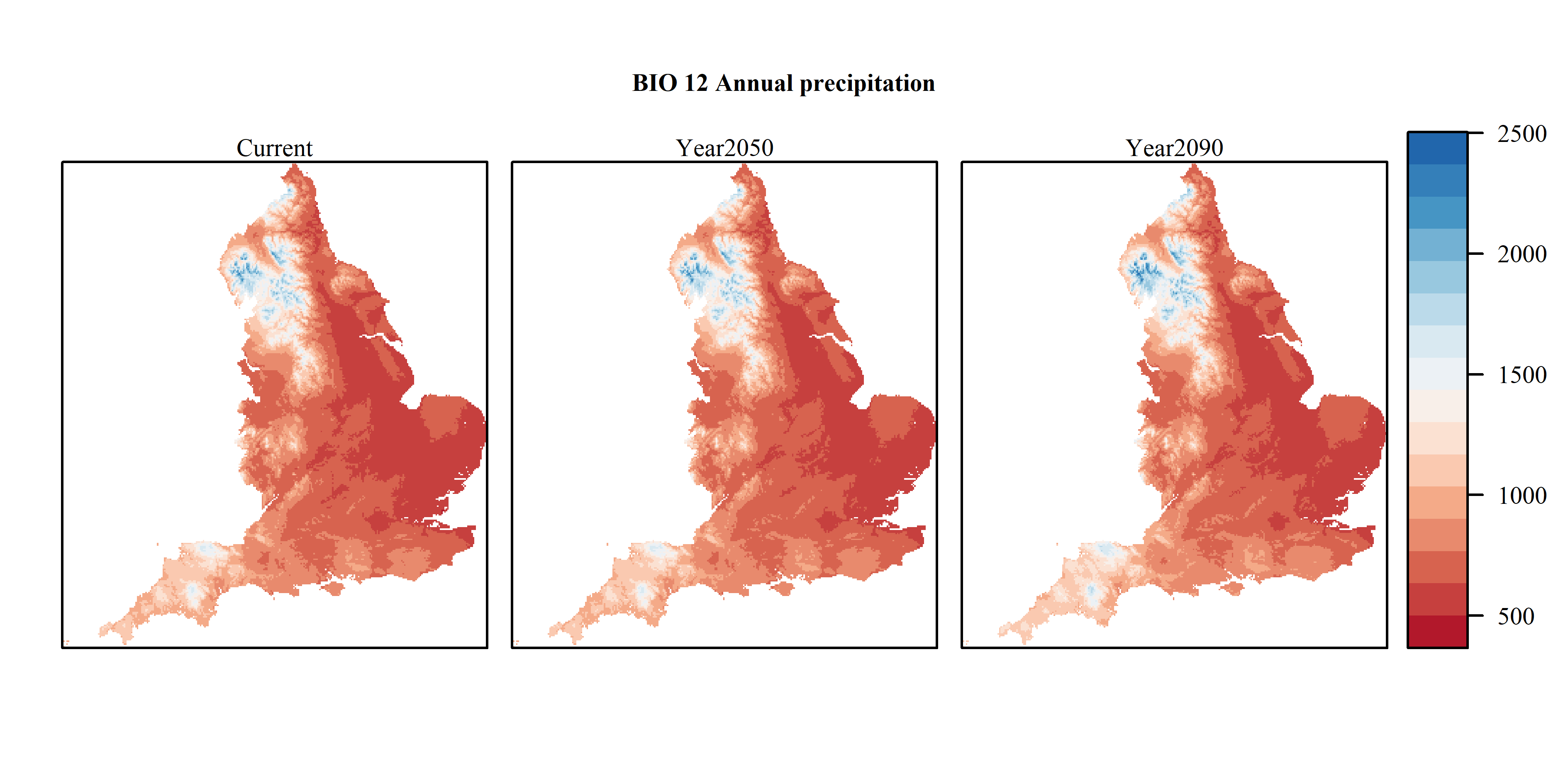

Supplement: Supplementary file 2 — Data S1: ece371956‐sup‐0002‐Supinfo.zip. [file ECE3-15-e71956-s001.zip › SUPPORTING.INFORMATION/ENVIRONMENTAL.VARIABLE.PREDICTOR.PLOTS/BIO.12.tif]

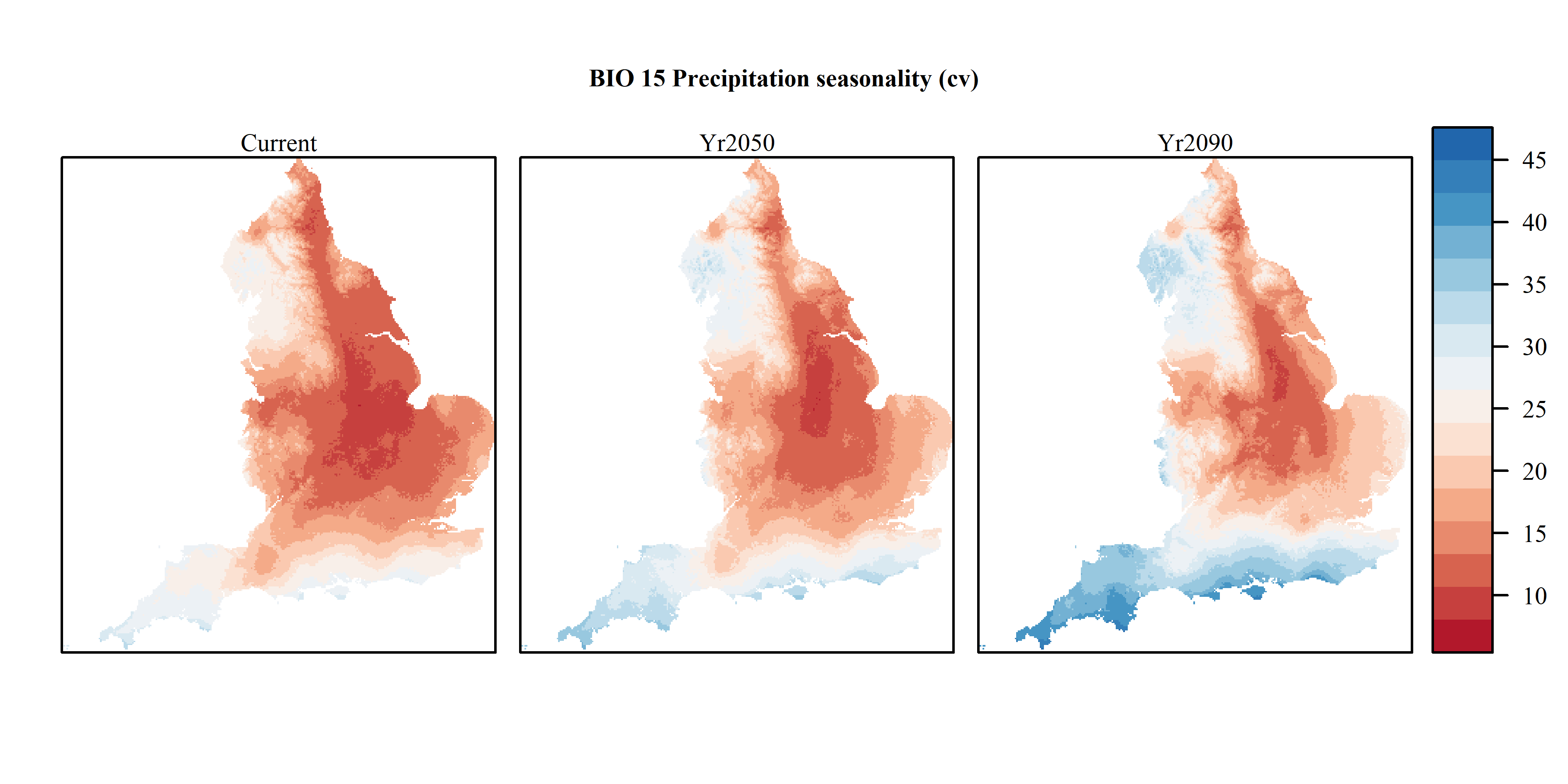

Supplement: Supplementary file 2 — Data S1: ece371956‐sup‐0002‐Supinfo.zip. [file ECE3-15-e71956-s001.zip › SUPPORTING.INFORMATION/ENVIRONMENTAL.VARIABLE.PREDICTOR.PLOTS/BIO.15.tif]

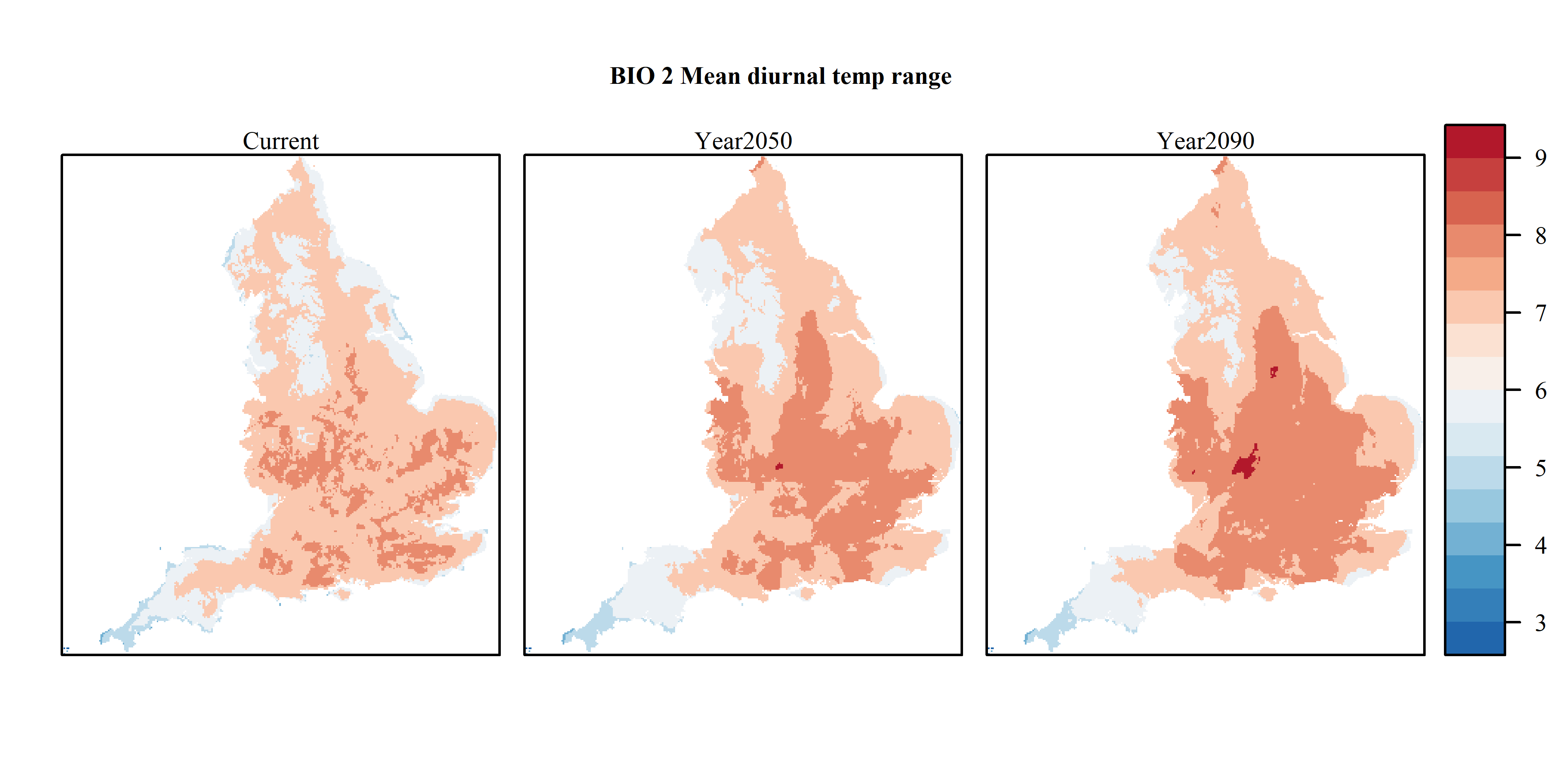

Supplement: Supplementary file 2 — Data S1: ece371956‐sup‐0002‐Supinfo.zip. [file ECE3-15-e71956-s001.zip › SUPPORTING.INFORMATION/ENVIRONMENTAL.VARIABLE.PREDICTOR.PLOTS/BIO.2.tif]

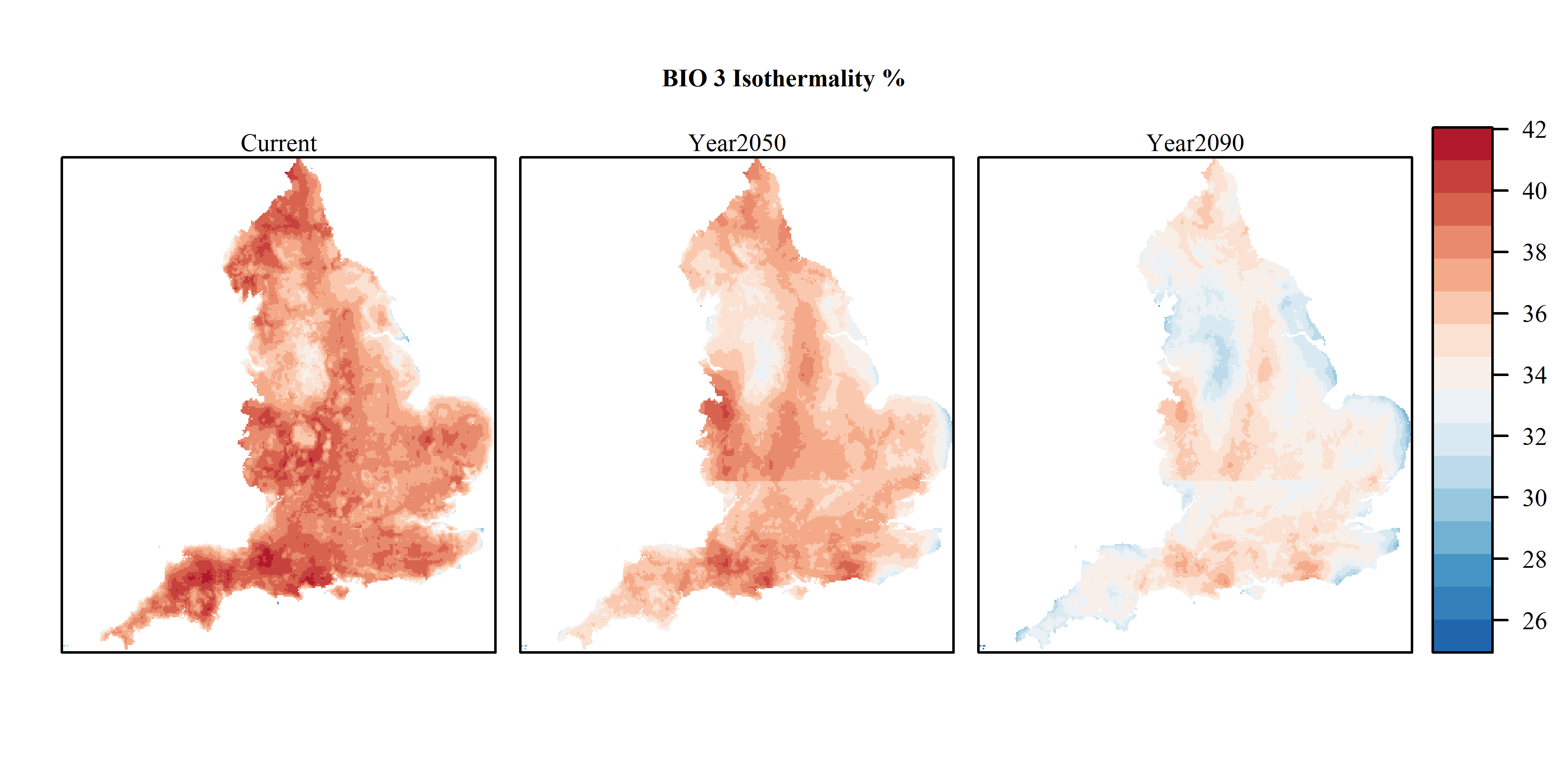

Supplement: Supplementary file 2 — Data S1: ece371956‐sup‐0002‐Supinfo.zip. [file ECE3-15-e71956-s001.zip › SUPPORTING.INFORMATION/ENVIRONMENTAL.VARIABLE.PREDICTOR.PLOTS/BIO.3.tif]

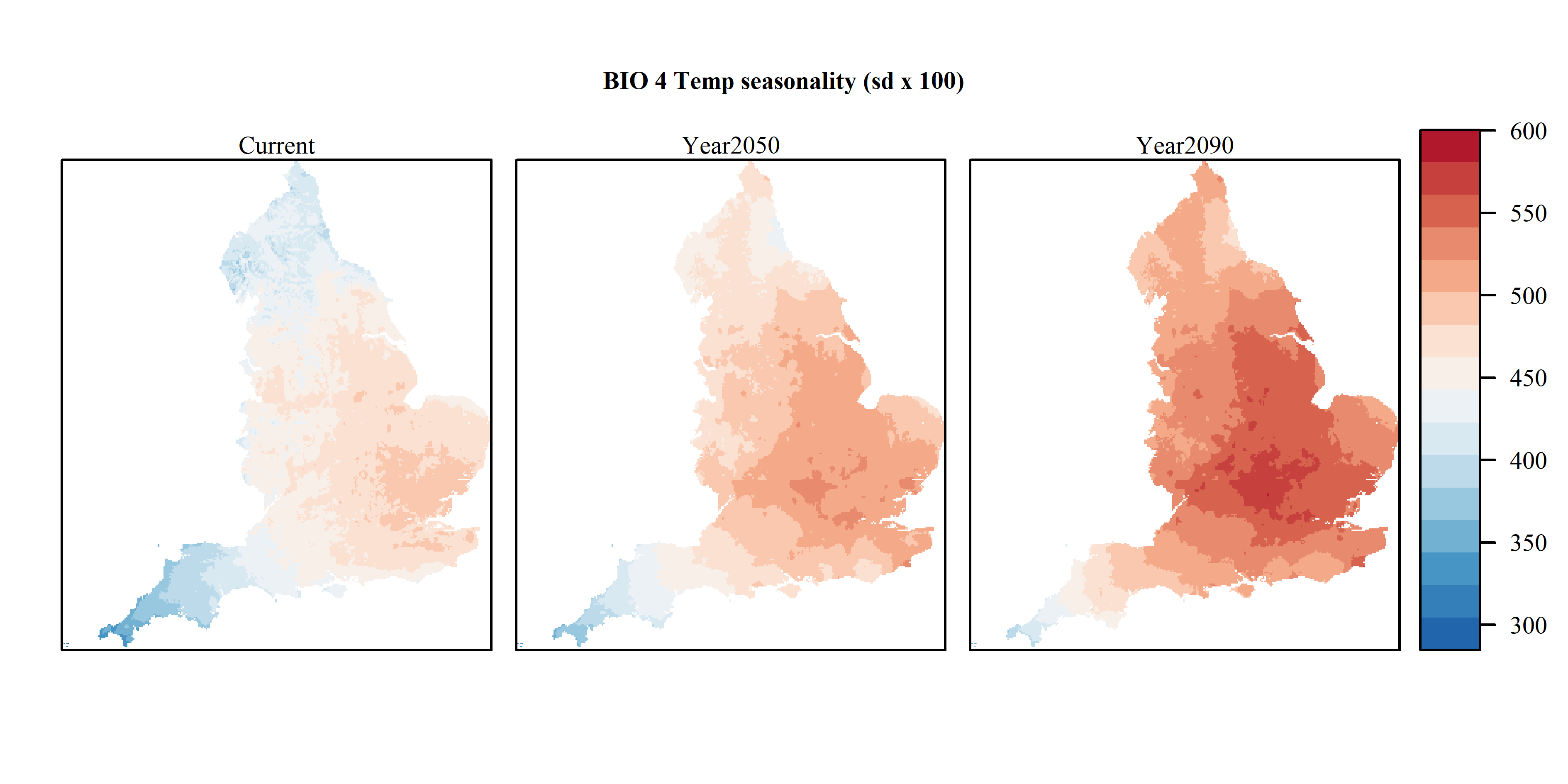

Supplement: Supplementary file 2 — Data S1: ece371956‐sup‐0002‐Supinfo.zip. [file ECE3-15-e71956-s001.zip › SUPPORTING.INFORMATION/ENVIRONMENTAL.VARIABLE.PREDICTOR.PLOTS/BIO.4.tif]

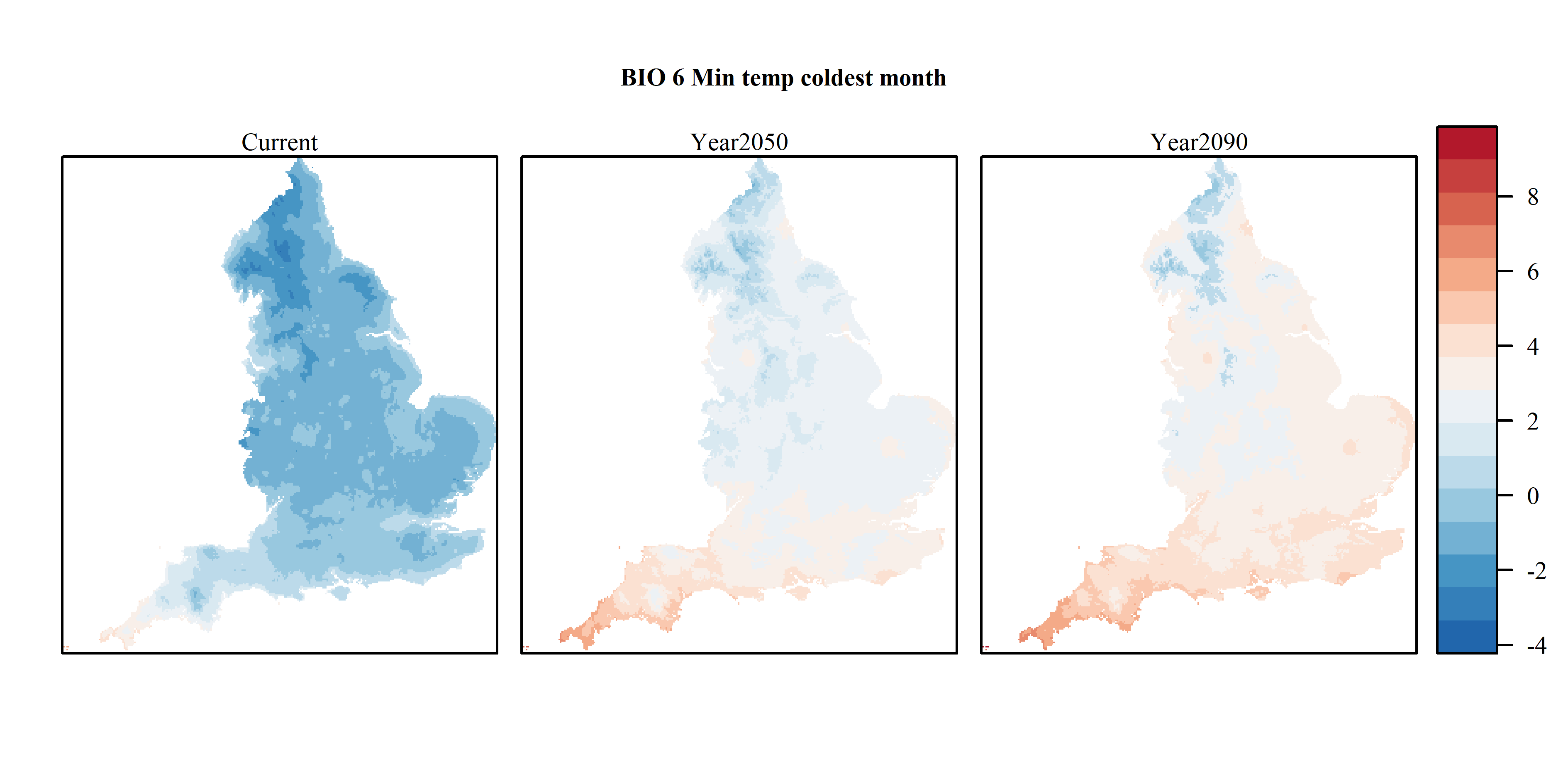

Supplement: Supplementary file 2 — Data S1: ece371956‐sup‐0002‐Supinfo.zip. [file ECE3-15-e71956-s001.zip › SUPPORTING.INFORMATION/ENVIRONMENTAL.VARIABLE.PREDICTOR.PLOTS/BIO.6.tif]

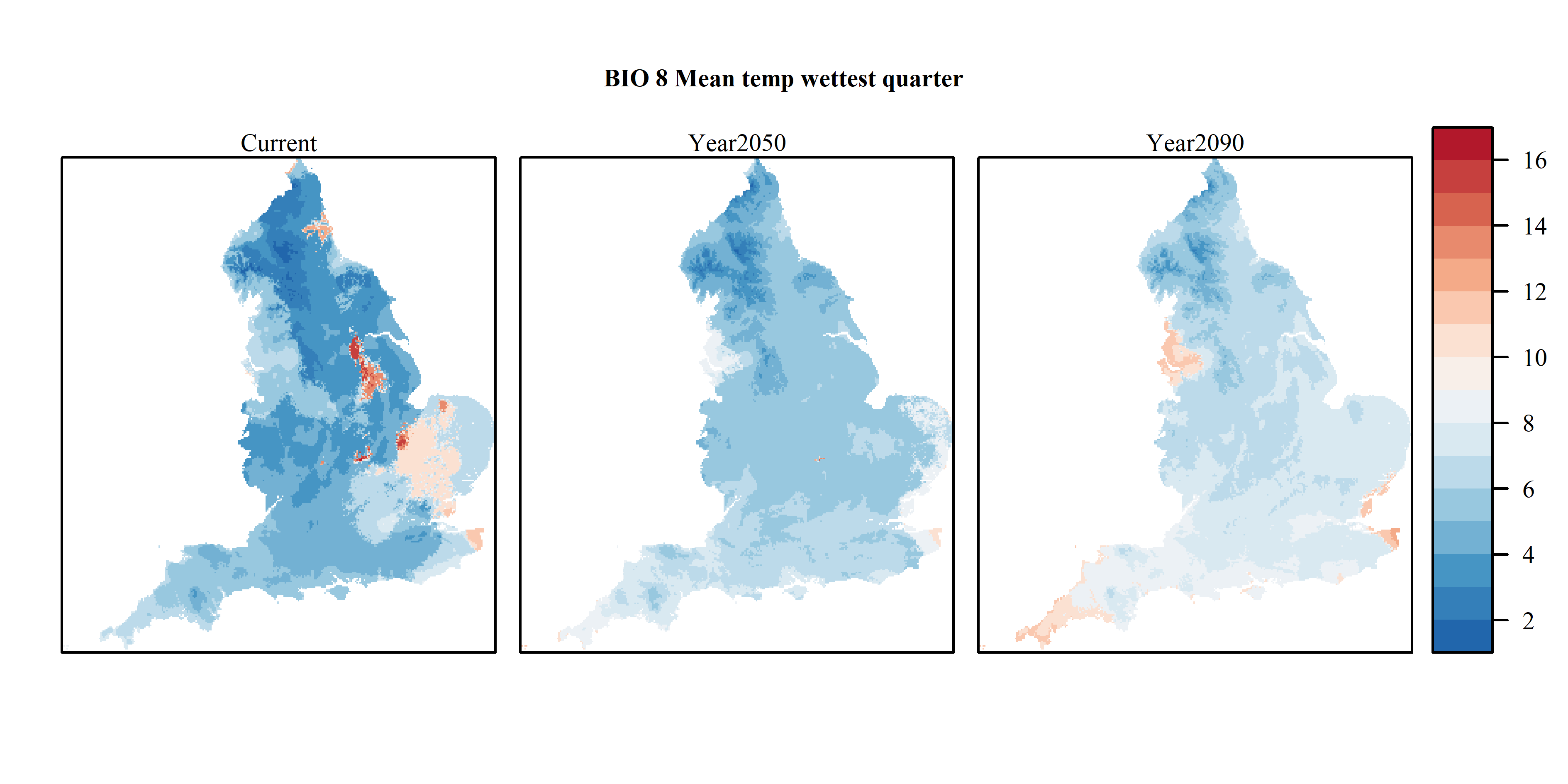

Supplement: Supplementary file 2 — Data S1: ece371956‐sup‐0002‐Supinfo.zip. [file ECE3-15-e71956-s001.zip › SUPPORTING.INFORMATION/ENVIRONMENTAL.VARIABLE.PREDICTOR.PLOTS/BIO.8.tif]

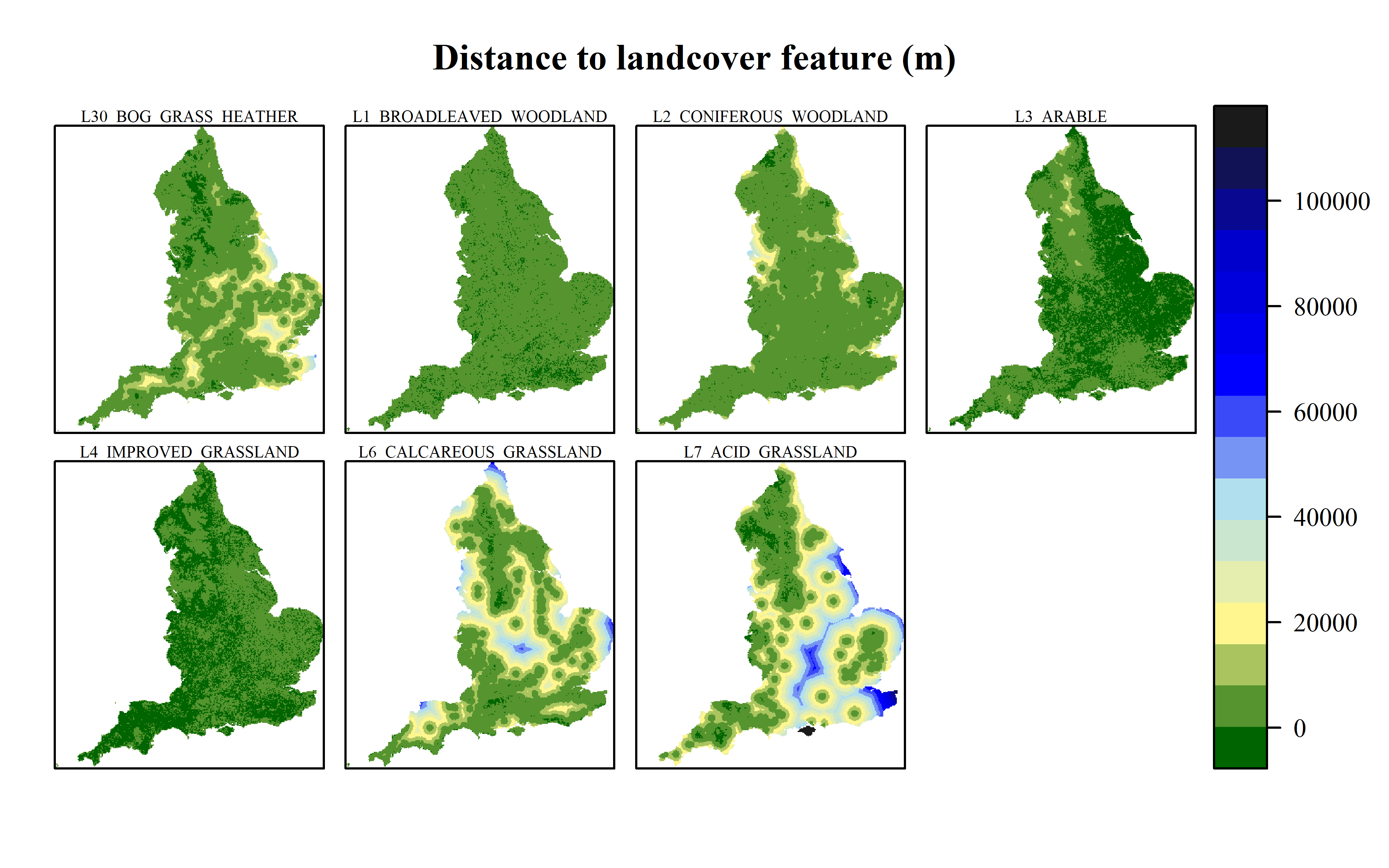

Supplement: Supplementary file 2 — Data S1: ece371956‐sup‐0002‐Supinfo.zip. [file ECE3-15-e71956-s001.zip › SUPPORTING.INFORMATION/ENVIRONMENTAL.VARIABLE.PREDICTOR.PLOTS/LANDCOVER.tif]

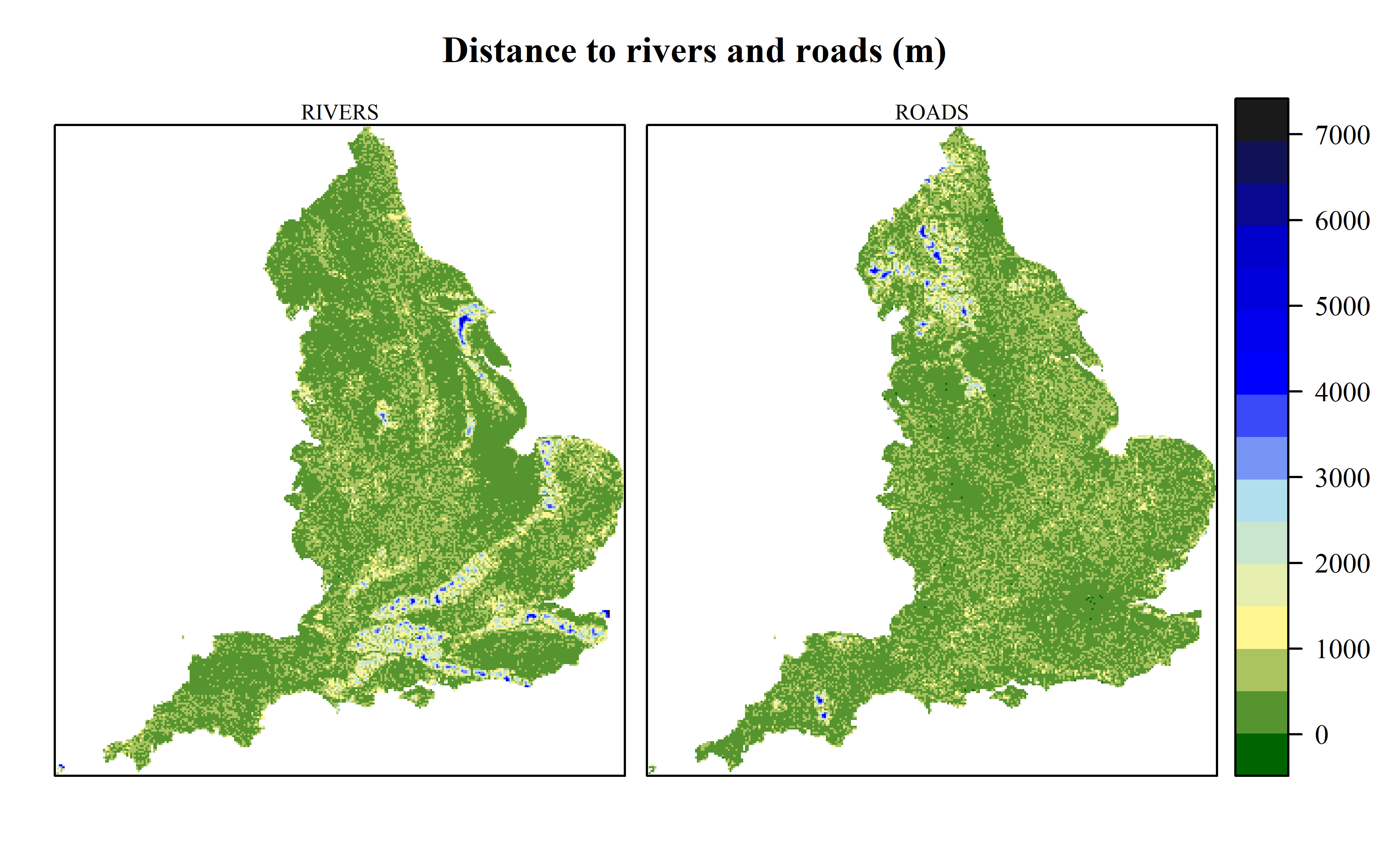

Supplement: Supplementary file 2 — Data S1: ece371956‐sup‐0002‐Supinfo.zip. [file ECE3-15-e71956-s001.zip › SUPPORTING.INFORMATION/ENVIRONMENTAL.VARIABLE.PREDICTOR.PLOTS/RIVERS.AND.ROADS.tif]

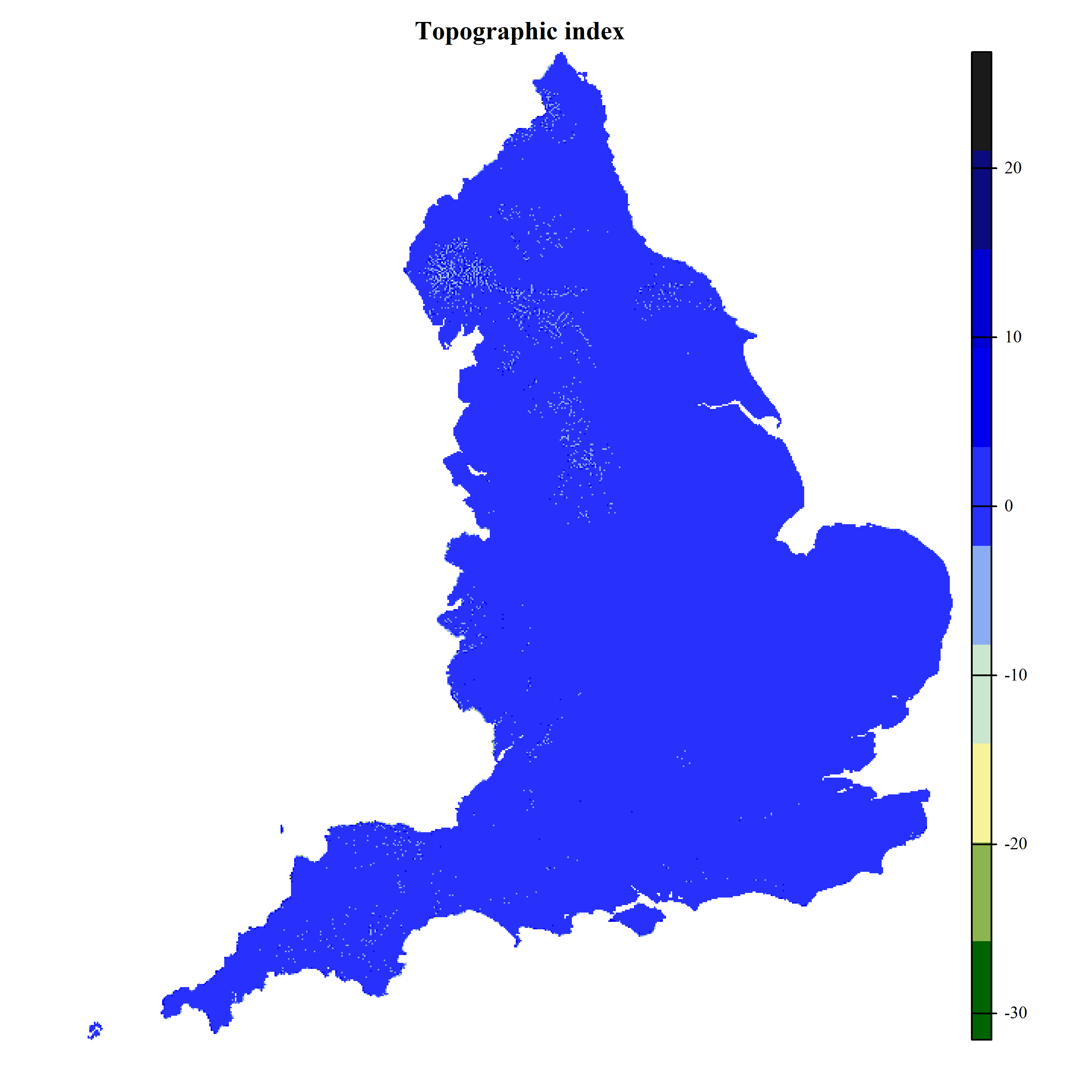

Supplement: Supplementary file 2 — Data S1: ece371956‐sup‐0002‐Supinfo.zip. [file ECE3-15-e71956-s001.zip › SUPPORTING.INFORMATION/ENVIRONMENTAL.VARIABLE.PREDICTOR.PLOTS/TOPO.INDEX.tif]

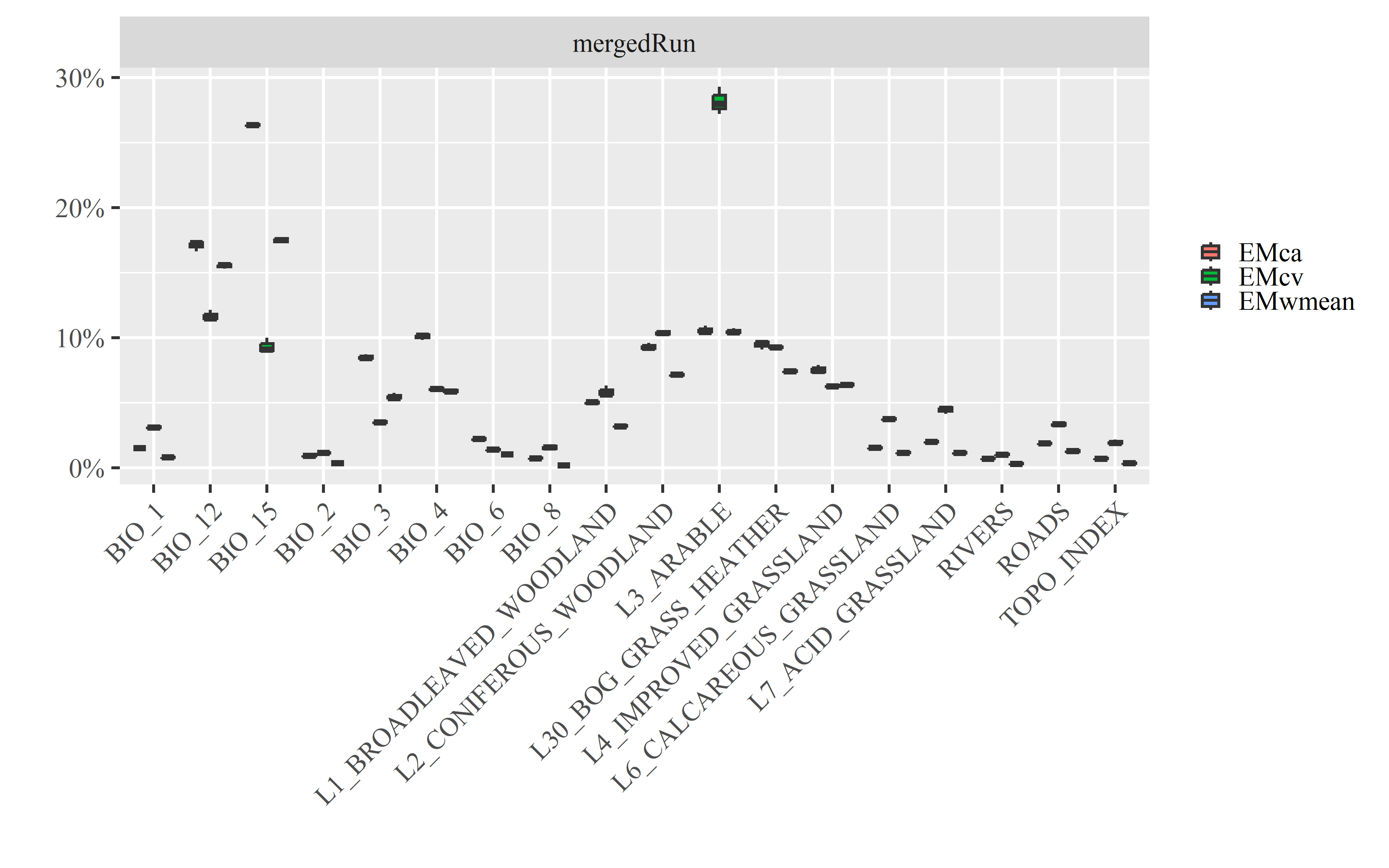

Supplement: Supplementary file 2 — Data S1: ece371956‐sup‐0002‐Supinfo.zip. [file ECE3-15-e71956-s001.zip › SUPPORTING.INFORMATION/SDM.VARIABLE.IMPORTANCE.PLOTS/ADDER.tif]

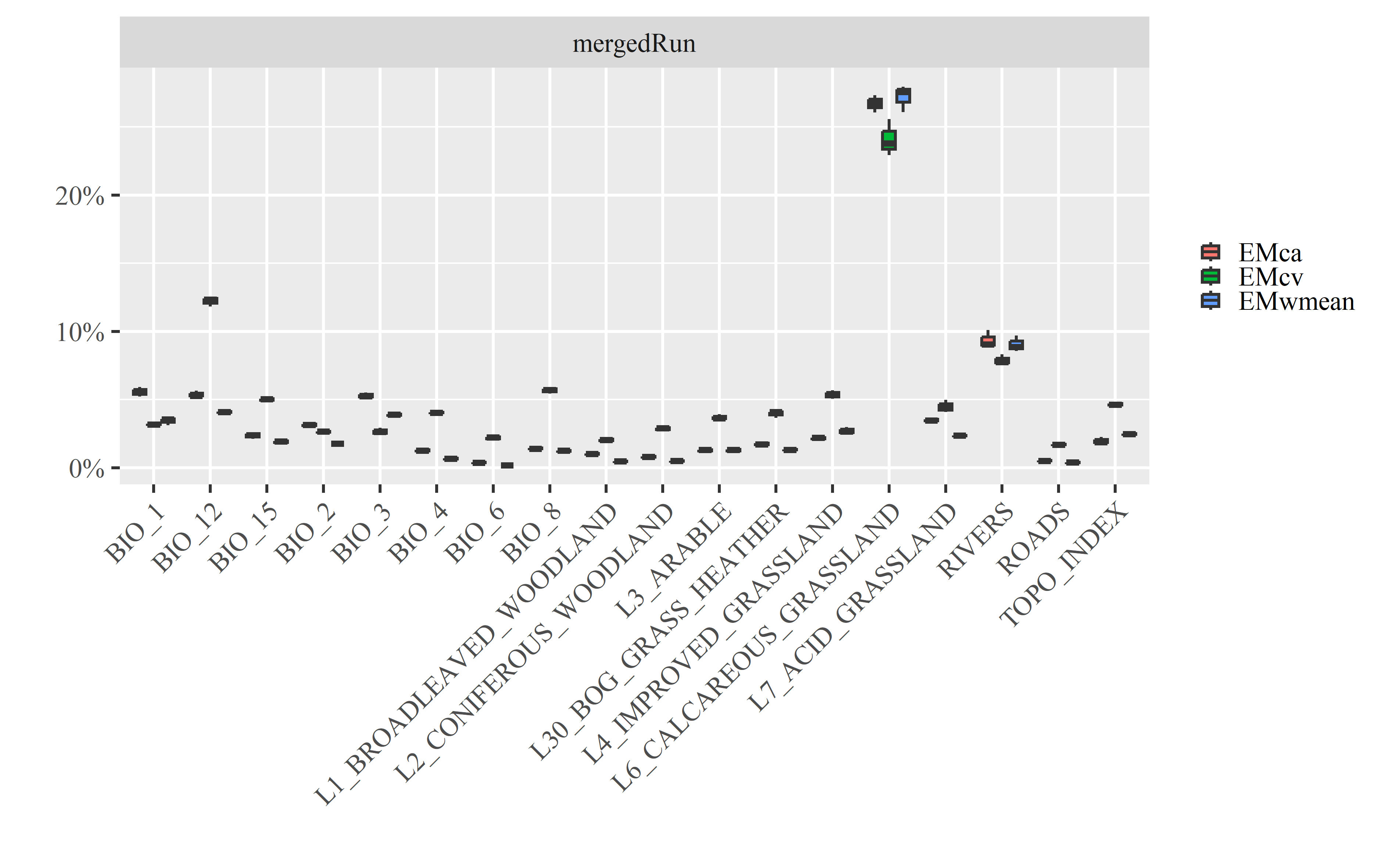

Supplement: Supplementary file 2 — Data S1: ece371956‐sup‐0002‐Supinfo.zip. [file ECE3-15-e71956-s001.zip › SUPPORTING.INFORMATION/SDM.VARIABLE.IMPORTANCE.PLOTS/CHALK.CARPET.tif]

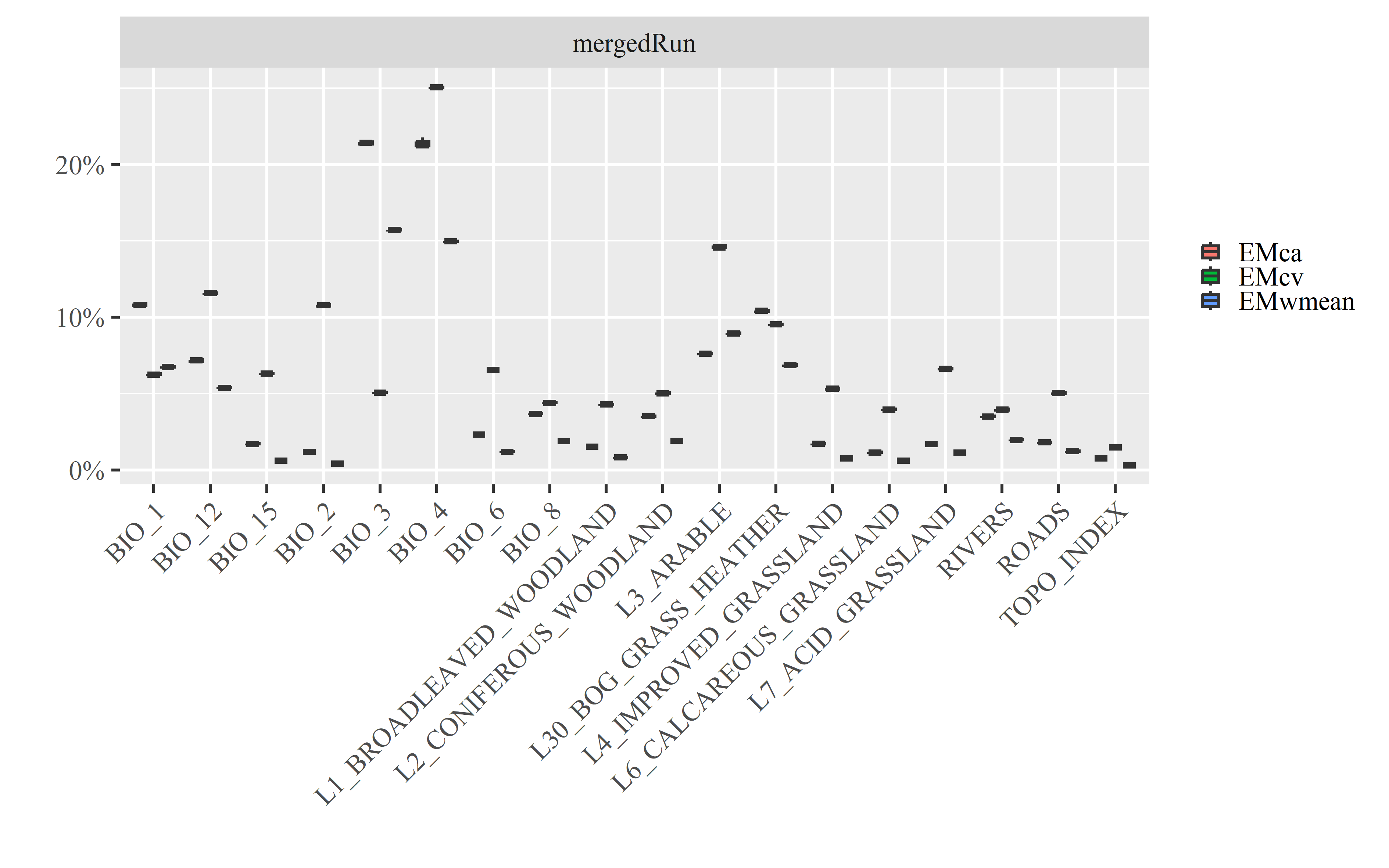

Supplement: Supplementary file 2 — Data S1: ece371956‐sup‐0002‐Supinfo.zip. [file ECE3-15-e71956-s001.zip › SUPPORTING.INFORMATION/SDM.VARIABLE.IMPORTANCE.PLOTS/CURLEW.tif]

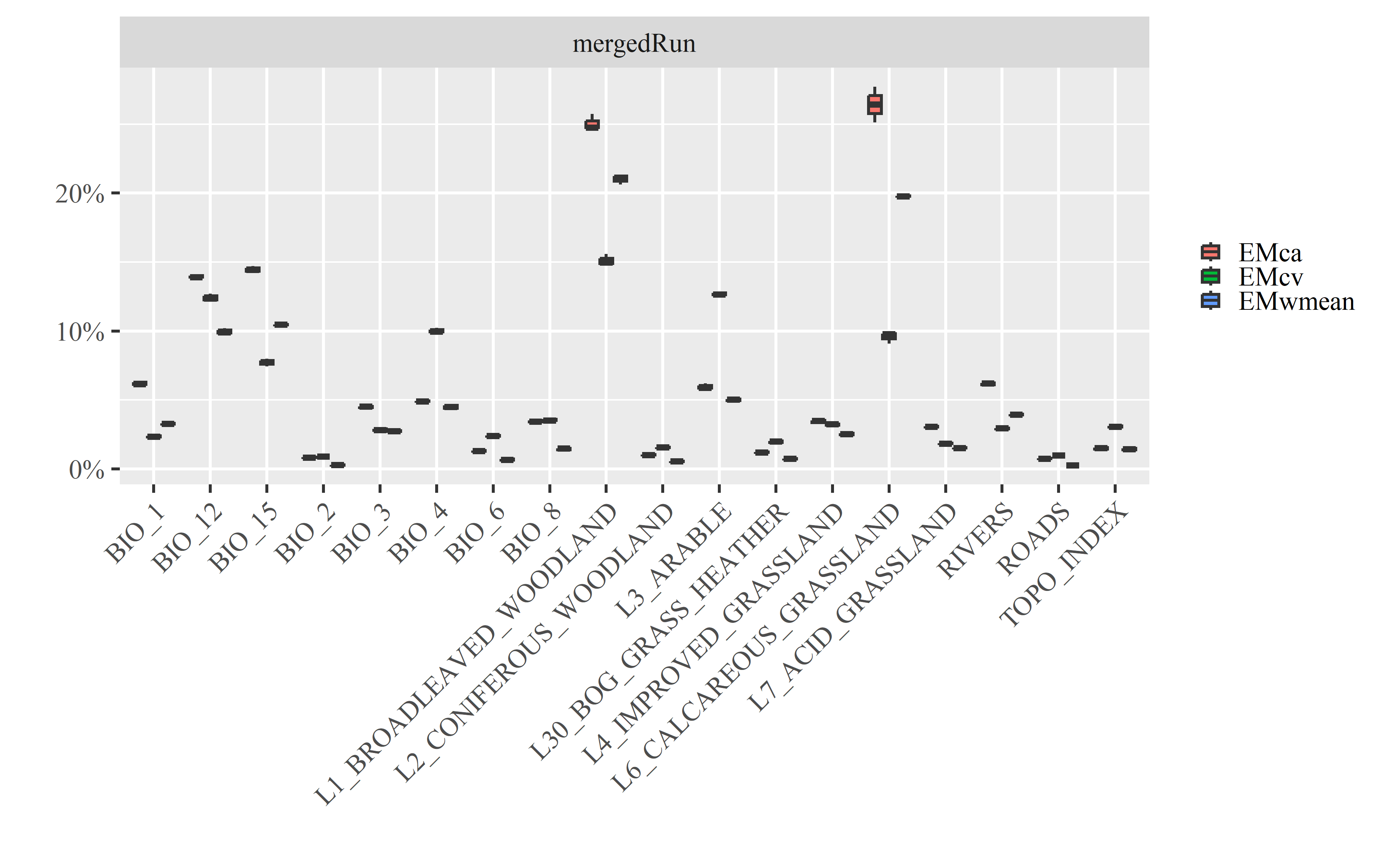

Supplement: Supplementary file 2 — Data S1: ece371956‐sup‐0002‐Supinfo.zip. [file ECE3-15-e71956-s001.zip › SUPPORTING.INFORMATION/SDM.VARIABLE.IMPORTANCE.PLOTS/DINGY.SKIPPER.tif]

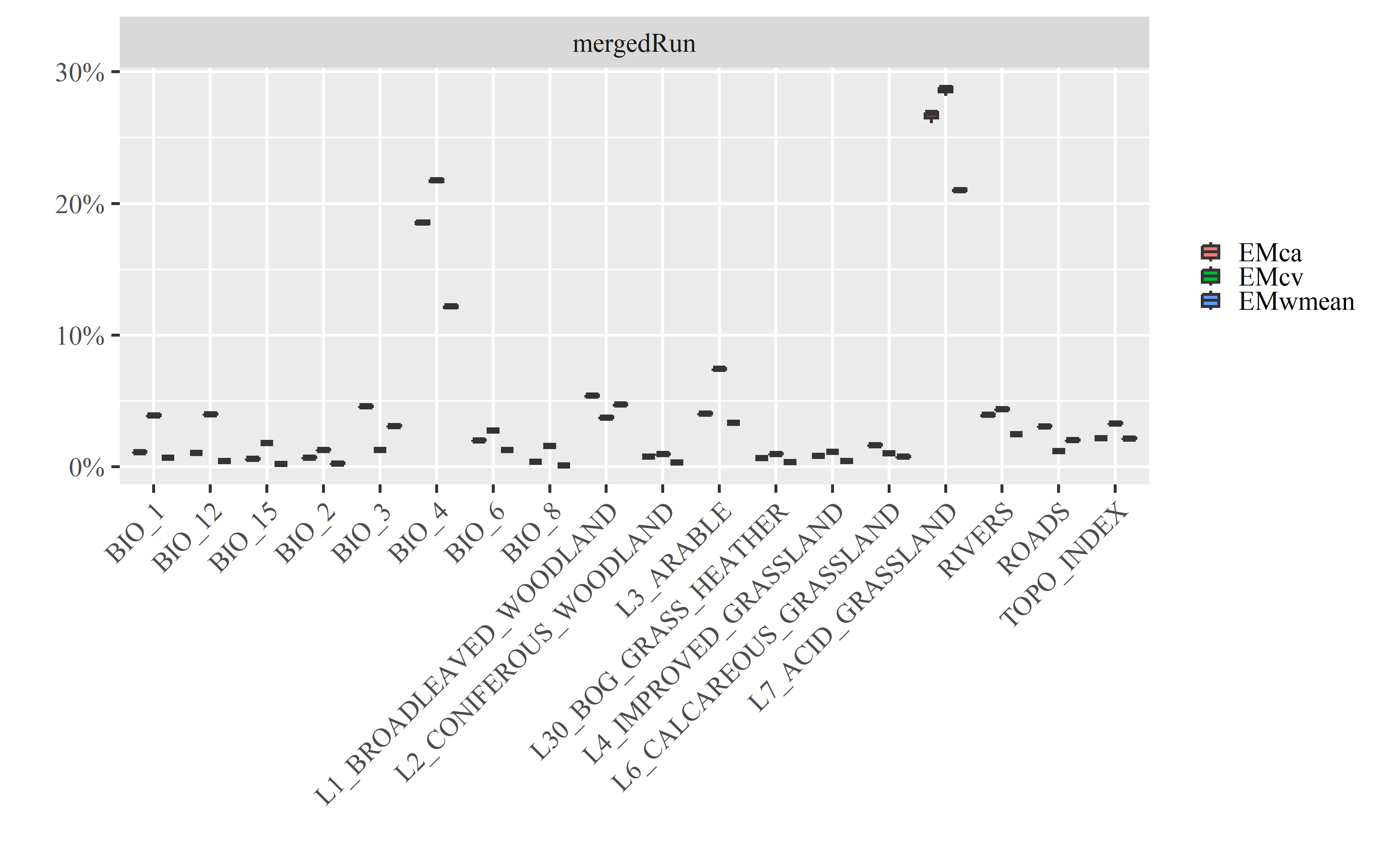

Supplement: Supplementary file 2 — Data S1: ece371956‐sup‐0002‐Supinfo.zip. [file ECE3-15-e71956-s001.zip › SUPPORTING.INFORMATION/SDM.VARIABLE.IMPORTANCE.PLOTS/DIPPER.tif]

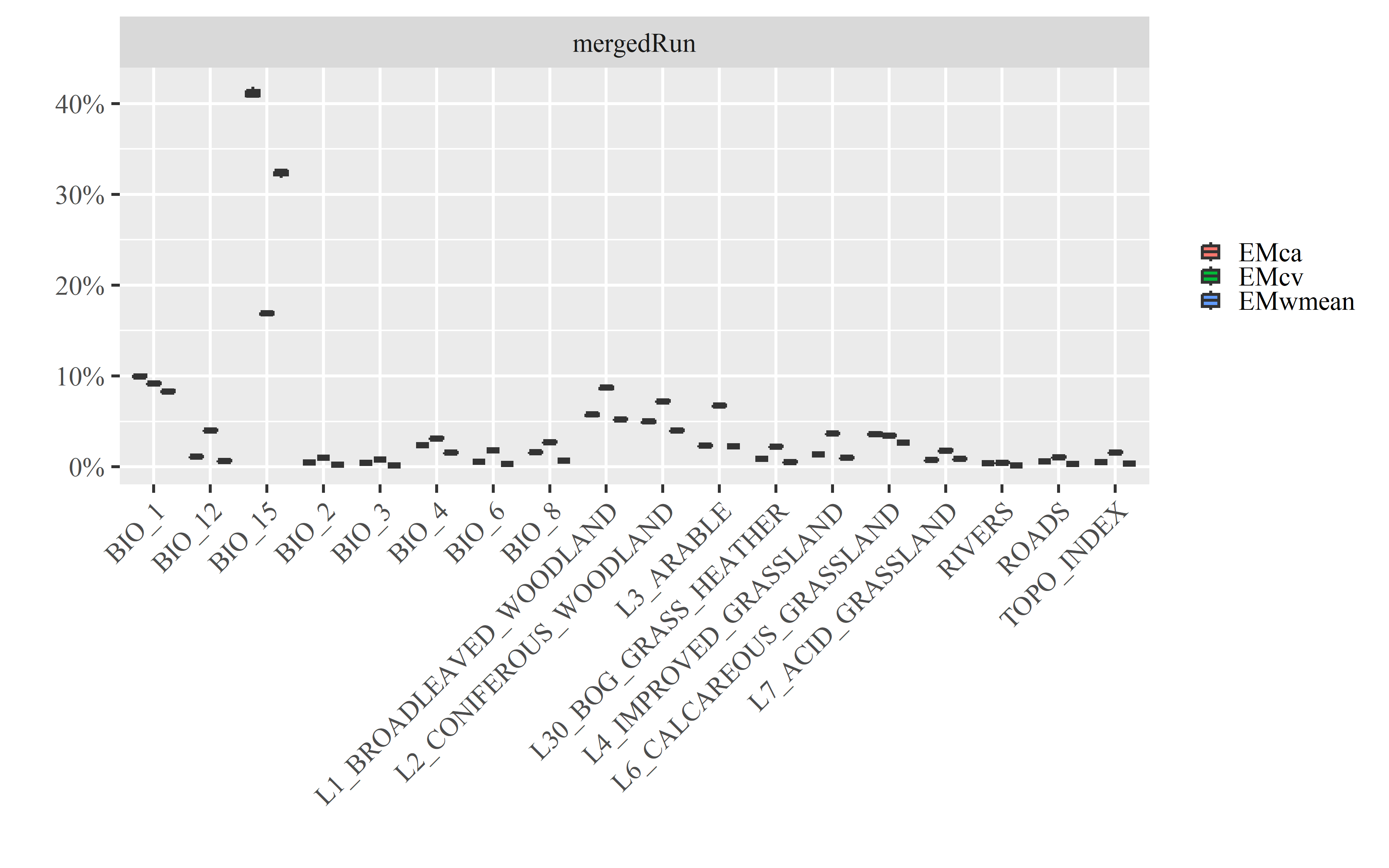

Supplement: Supplementary file 2 — Data S1: ece371956‐sup‐0002‐Supinfo.zip. [file ECE3-15-e71956-s001.zip › SUPPORTING.INFORMATION/SDM.VARIABLE.IMPORTANCE.PLOTS/HAZEL.DORMOUSE.tif]

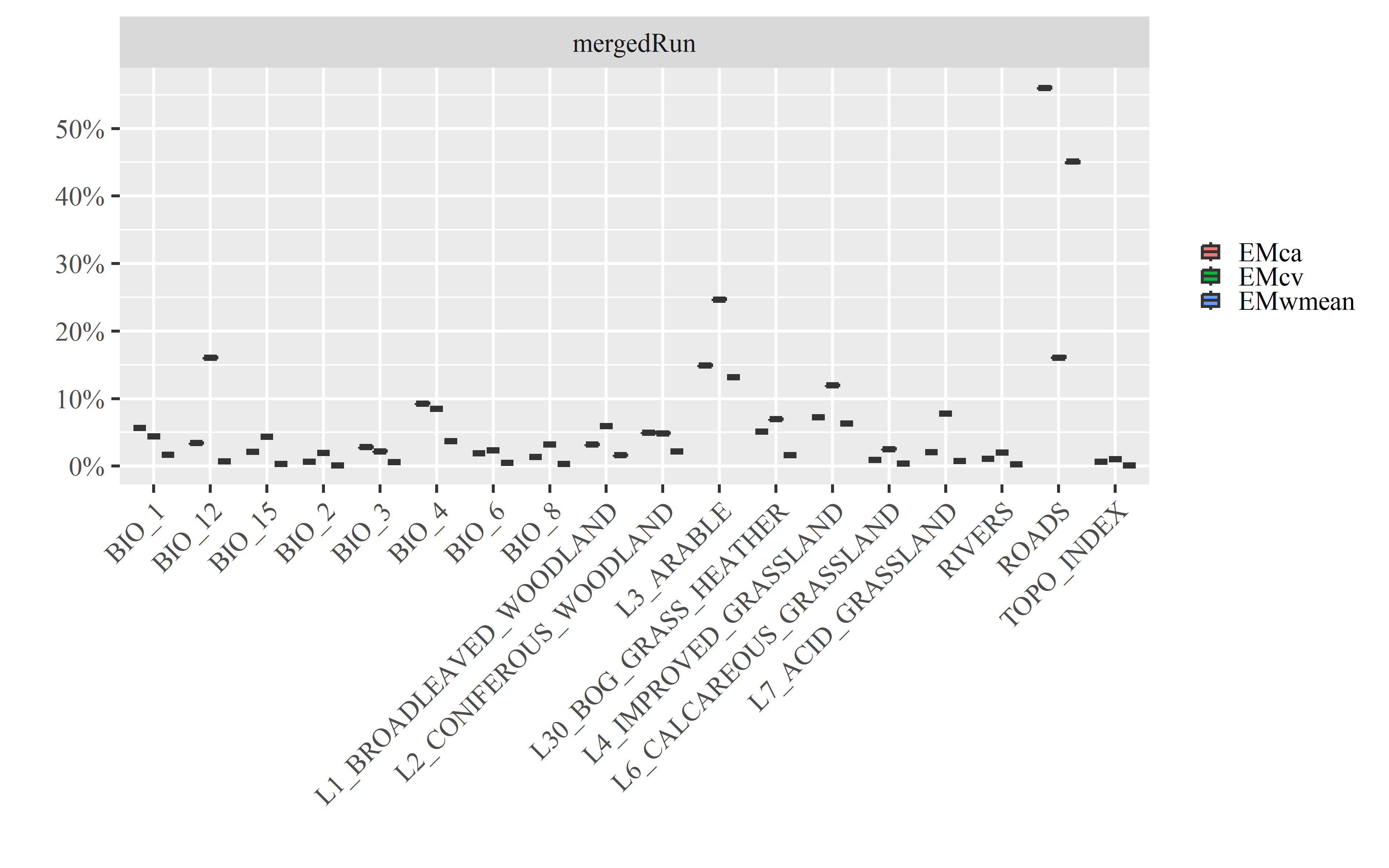

Supplement: Supplementary file 2 — Data S1: ece371956‐sup‐0002‐Supinfo.zip. [file ECE3-15-e71956-s001.zip › SUPPORTING.INFORMATION/SDM.VARIABLE.IMPORTANCE.PLOTS/HEDGEHOG.tif]

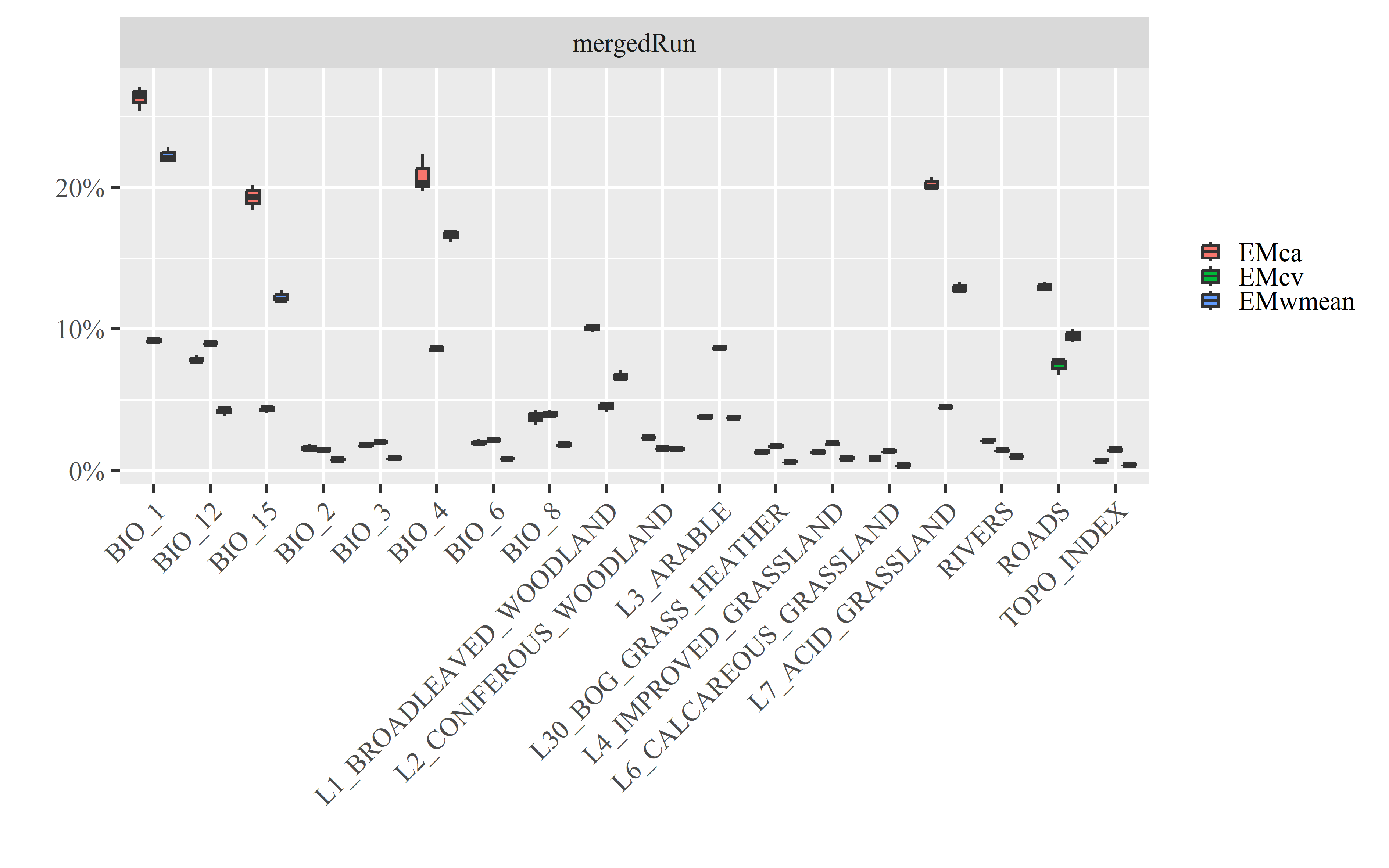

Supplement: Supplementary file 2 — Data S1: ece371956‐sup‐0002‐Supinfo.zip. [file ECE3-15-e71956-s001.zip › SUPPORTING.INFORMATION/SDM.VARIABLE.IMPORTANCE.PLOTS/LEISLERS.BAT.tif]

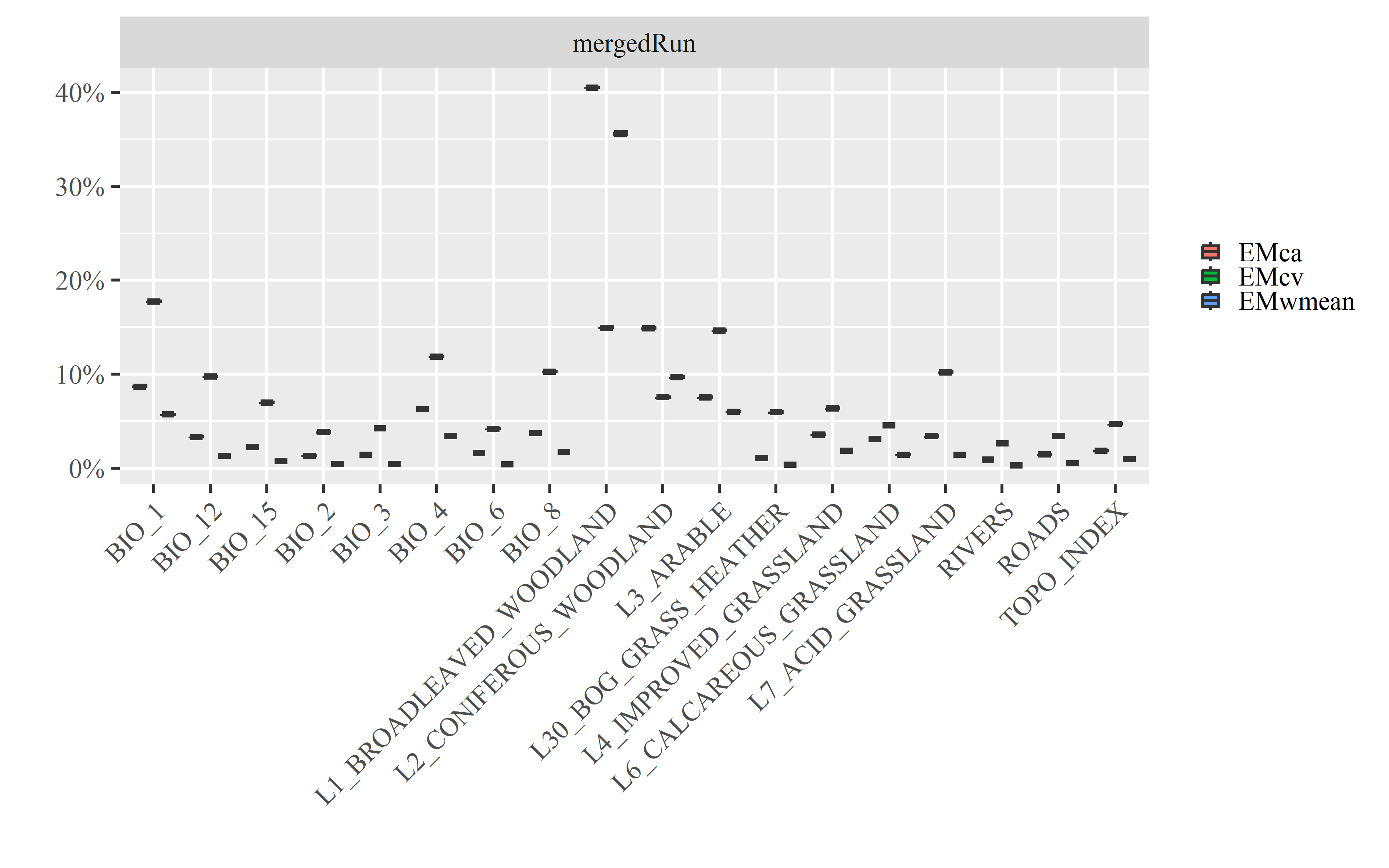

Supplement: Supplementary file 2 — Data S1: ece371956‐sup‐0002‐Supinfo.zip. [file ECE3-15-e71956-s001.zip › SUPPORTING.INFORMATION/SDM.VARIABLE.IMPORTANCE.PLOTS/MARSH.TIT.tif]

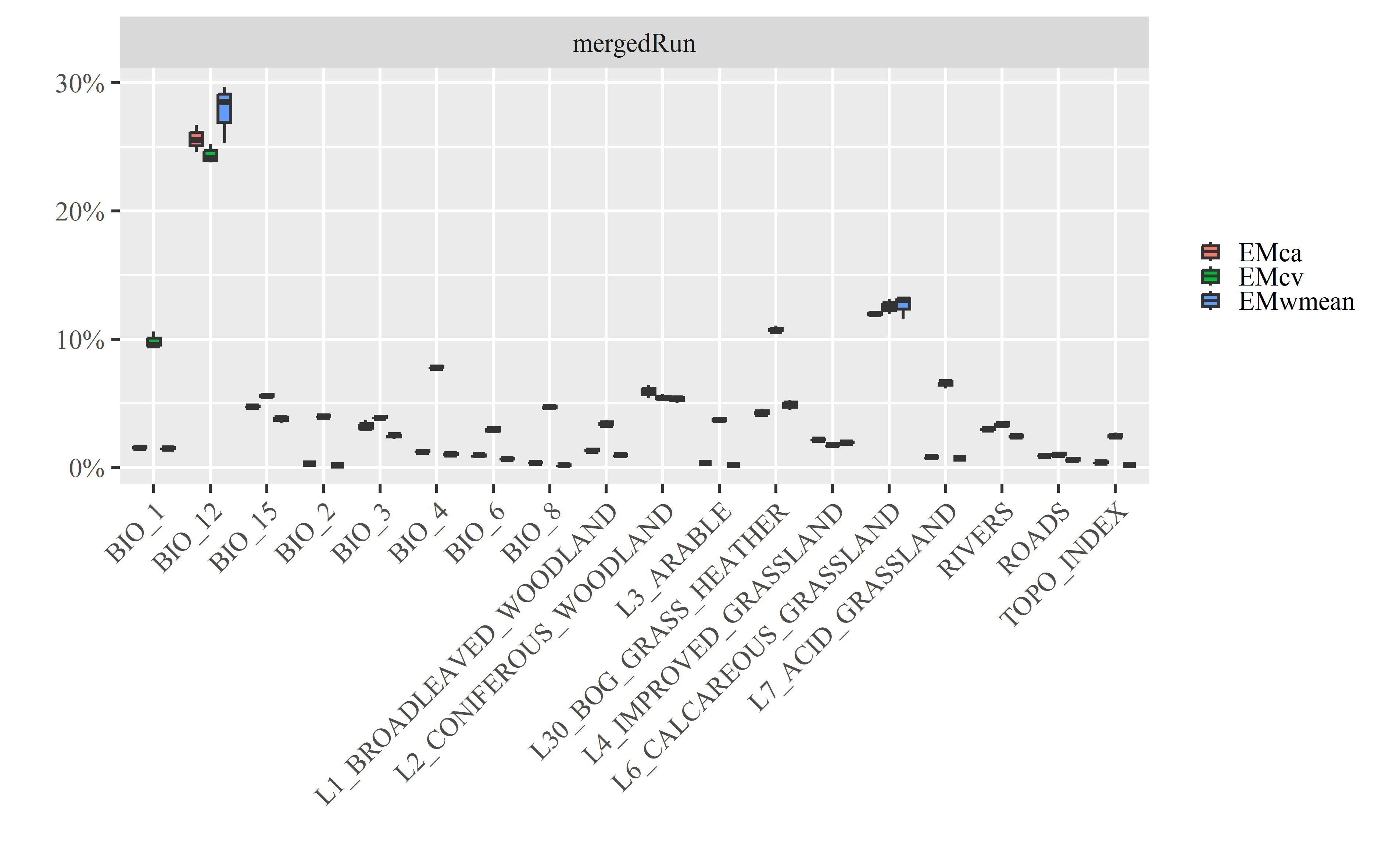

Supplement: Supplementary file 2 — Data S1: ece371956‐sup‐0002‐Supinfo.zip. [file ECE3-15-e71956-s001.zip › SUPPORTING.INFORMATION/SDM.VARIABLE.IMPORTANCE.PLOTS/N. BROWN.ARGUS.tif]

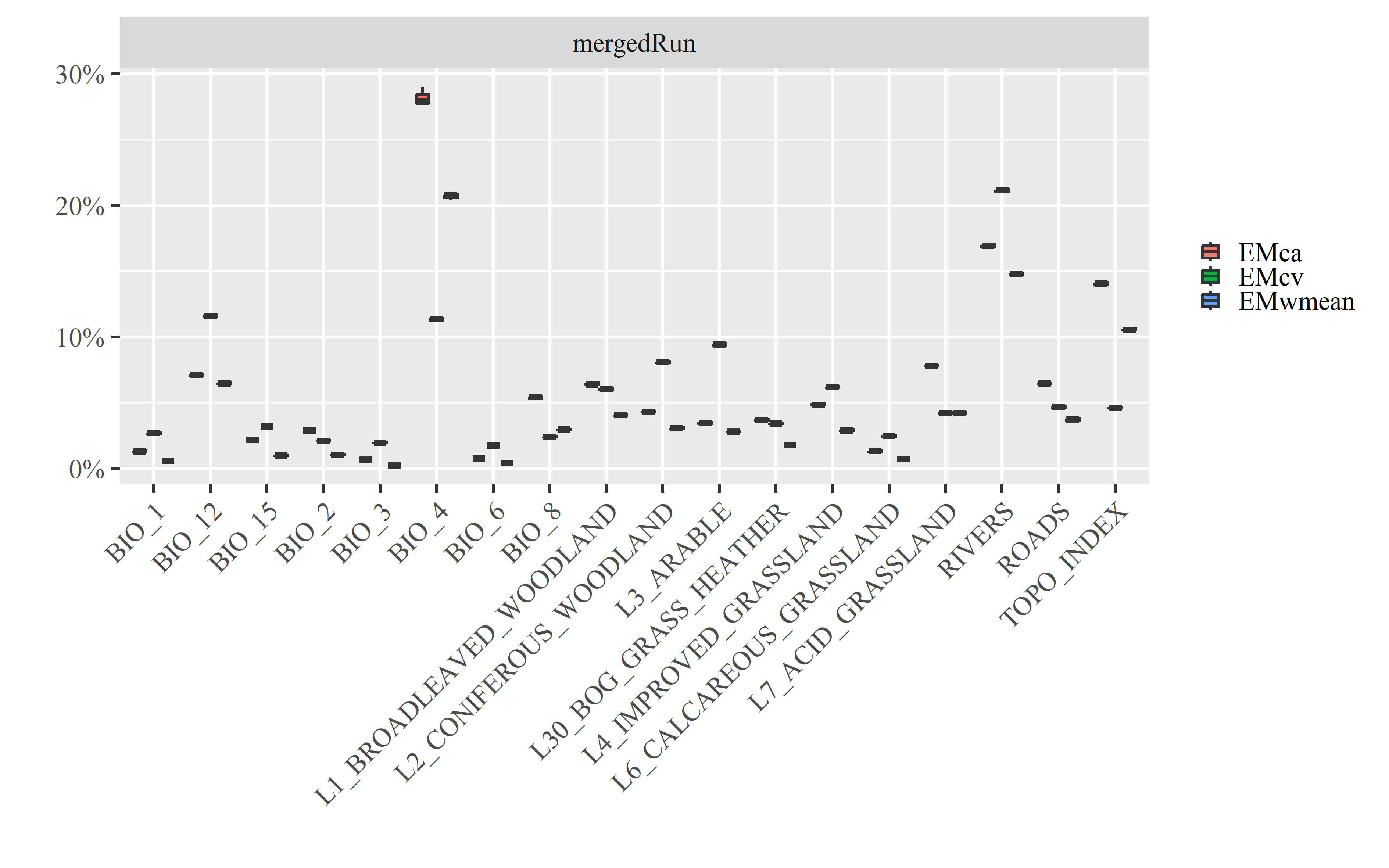

Supplement: Supplementary file 2 — Data S1: ece371956‐sup‐0002‐Supinfo.zip. [file ECE3-15-e71956-s001.zip › SUPPORTING.INFORMATION/SDM.VARIABLE.IMPORTANCE.PLOTS/OTTER.tif]

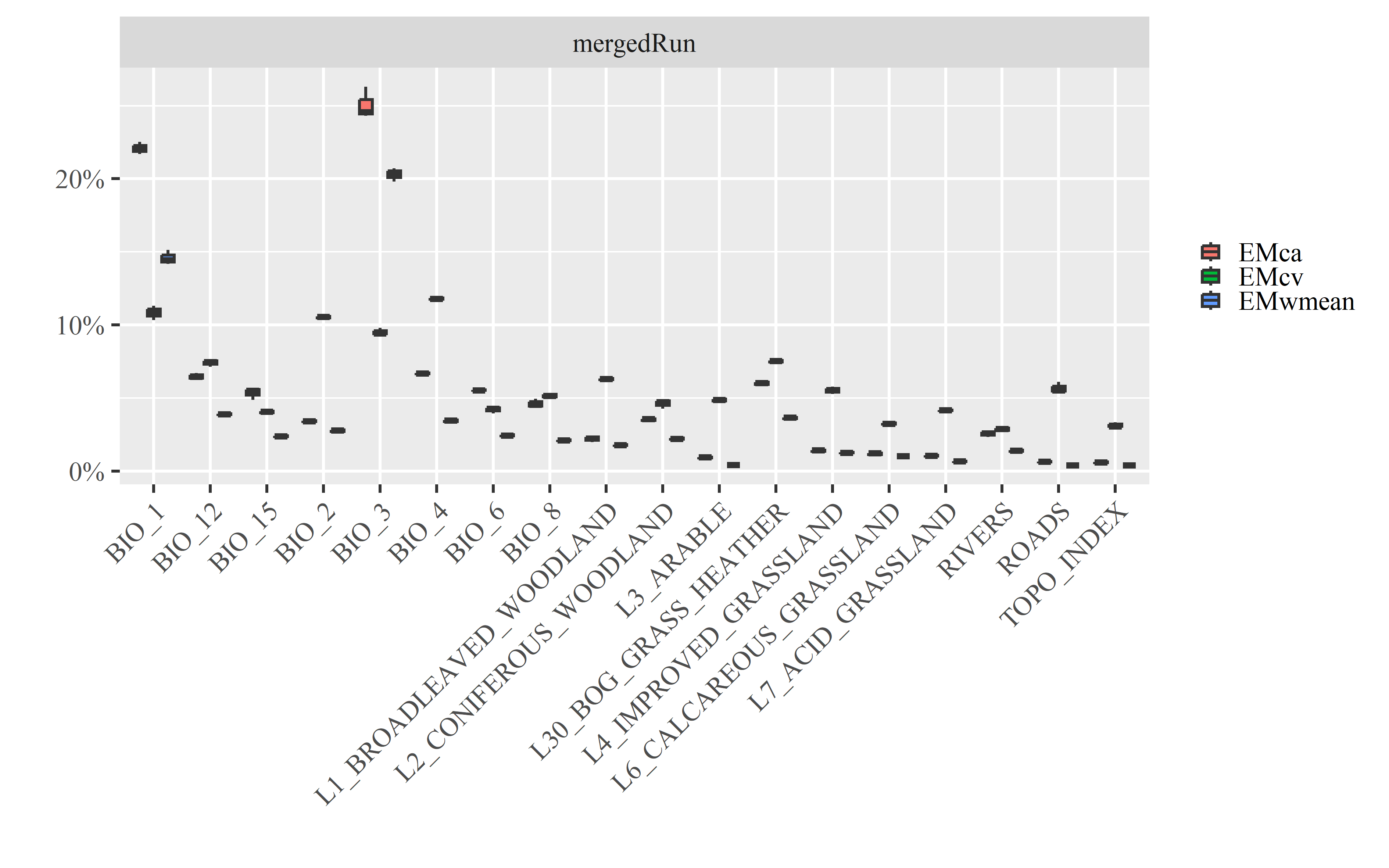

Supplement: Supplementary file 2 — Data S1: ece371956‐sup‐0002‐Supinfo.zip. [file ECE3-15-e71956-s001.zip › SUPPORTING.INFORMATION/SDM.VARIABLE.IMPORTANCE.PLOTS/TWITE.tif]

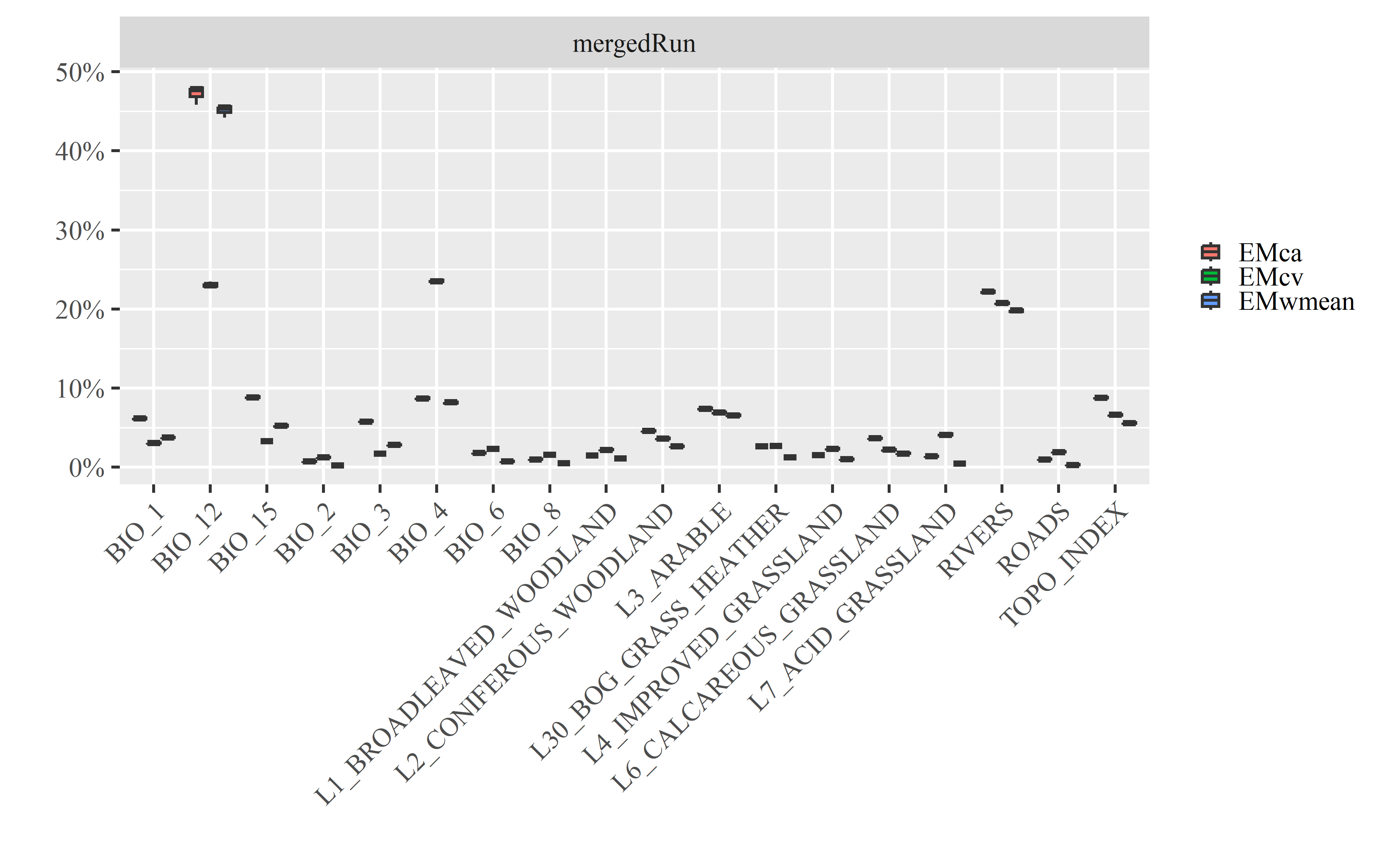

Supplement: Supplementary file 2 — Data S1: ece371956‐sup‐0002‐Supinfo.zip. [file ECE3-15-e71956-s001.zip › SUPPORTING.INFORMATION/SDM.VARIABLE.IMPORTANCE.PLOTS/WATER.VOLE.tif]

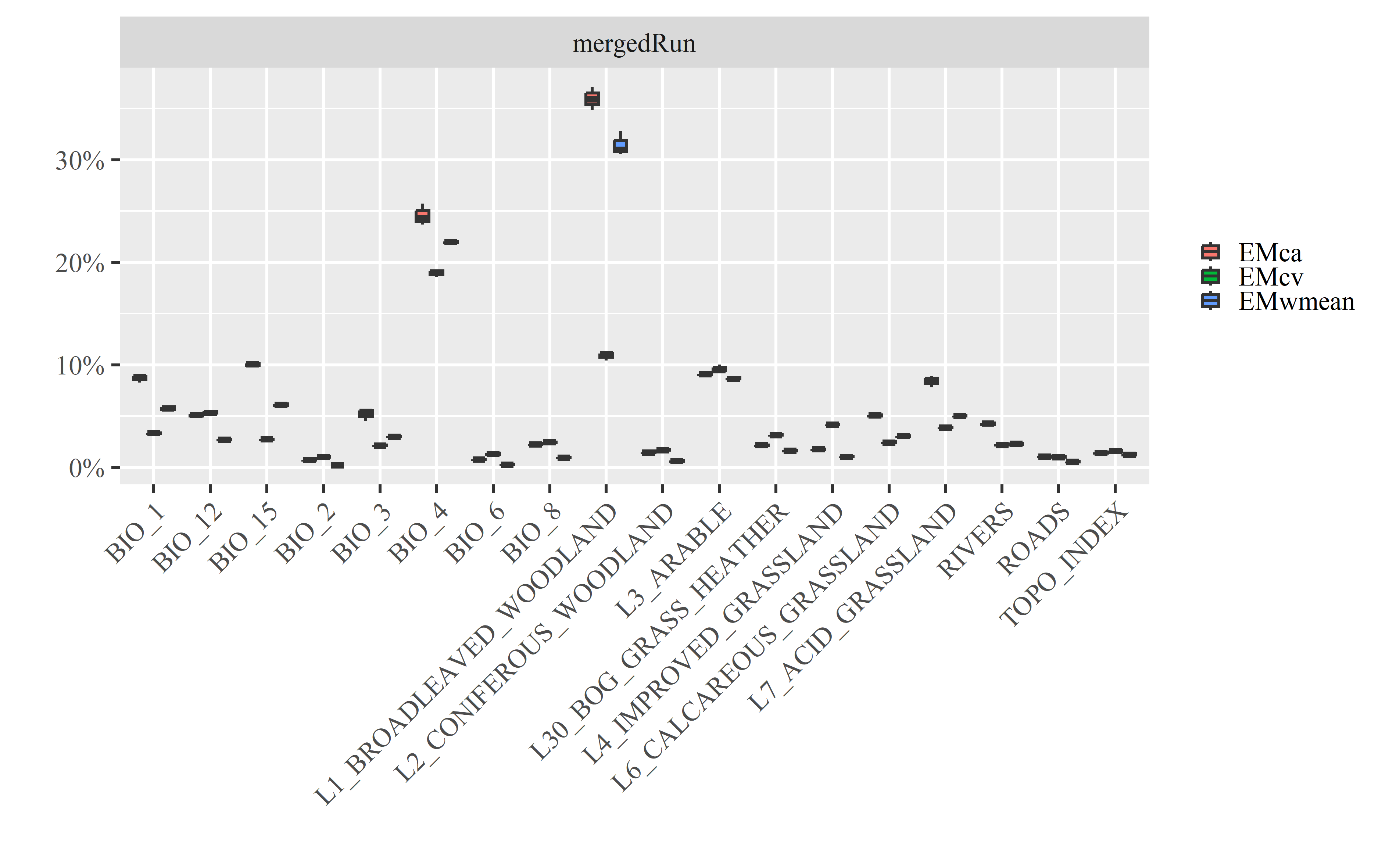

Supplement: Supplementary file 2 — Data S1: ece371956‐sup‐0002‐Supinfo.zip. [file ECE3-15-e71956-s001.zip › SUPPORTING.INFORMATION/SDM.VARIABLE.IMPORTANCE.PLOTS/WH.L. HAIRSTREAK.tif]

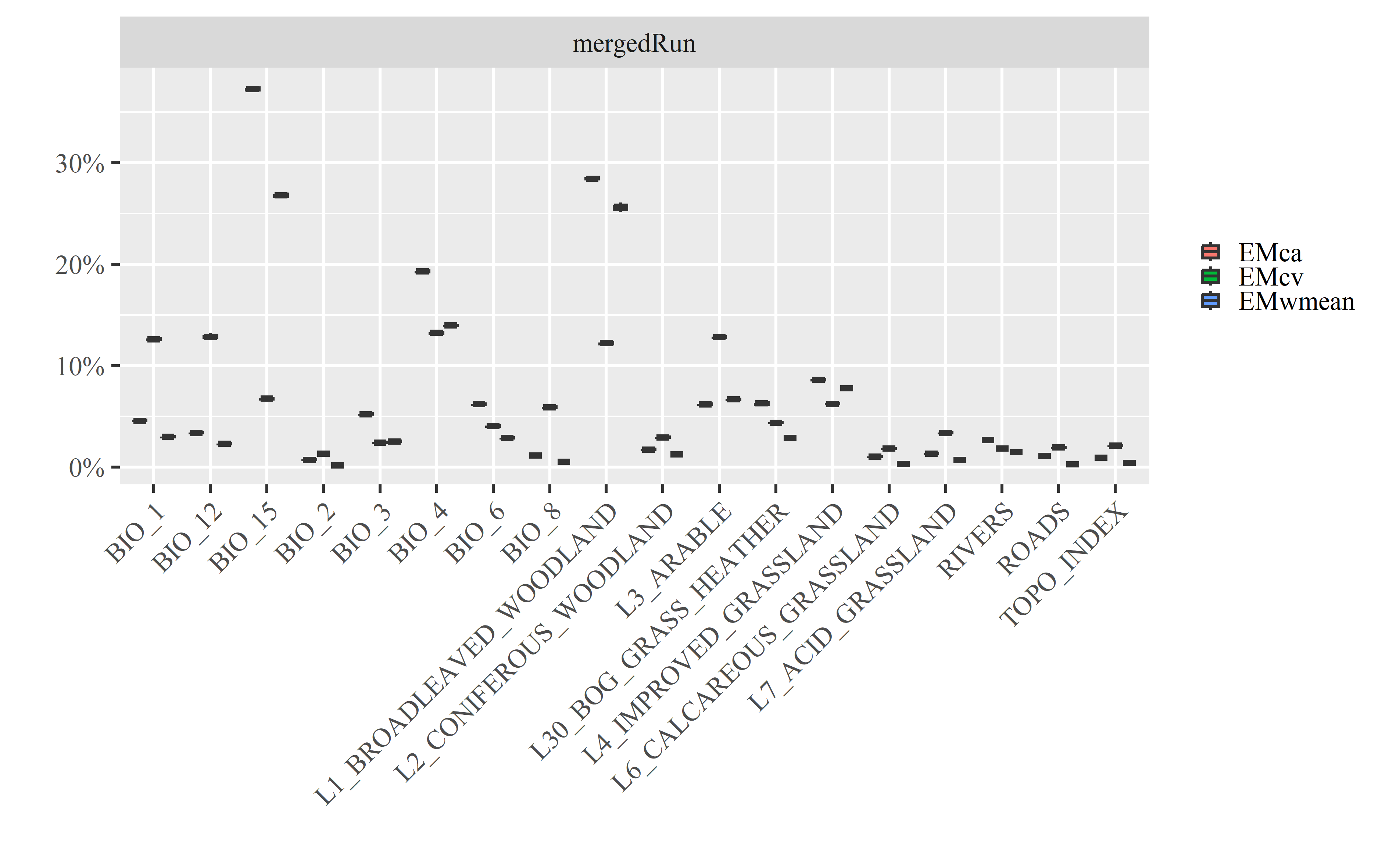

Supplement: Supplementary file 2 — Data S1: ece371956‐sup‐0002‐Supinfo.zip. [file ECE3-15-e71956-s001.zip › SUPPORTING.INFORMATION/SDM.VARIABLE.IMPORTANCE.PLOTS/WILLOW.TIT.tif]

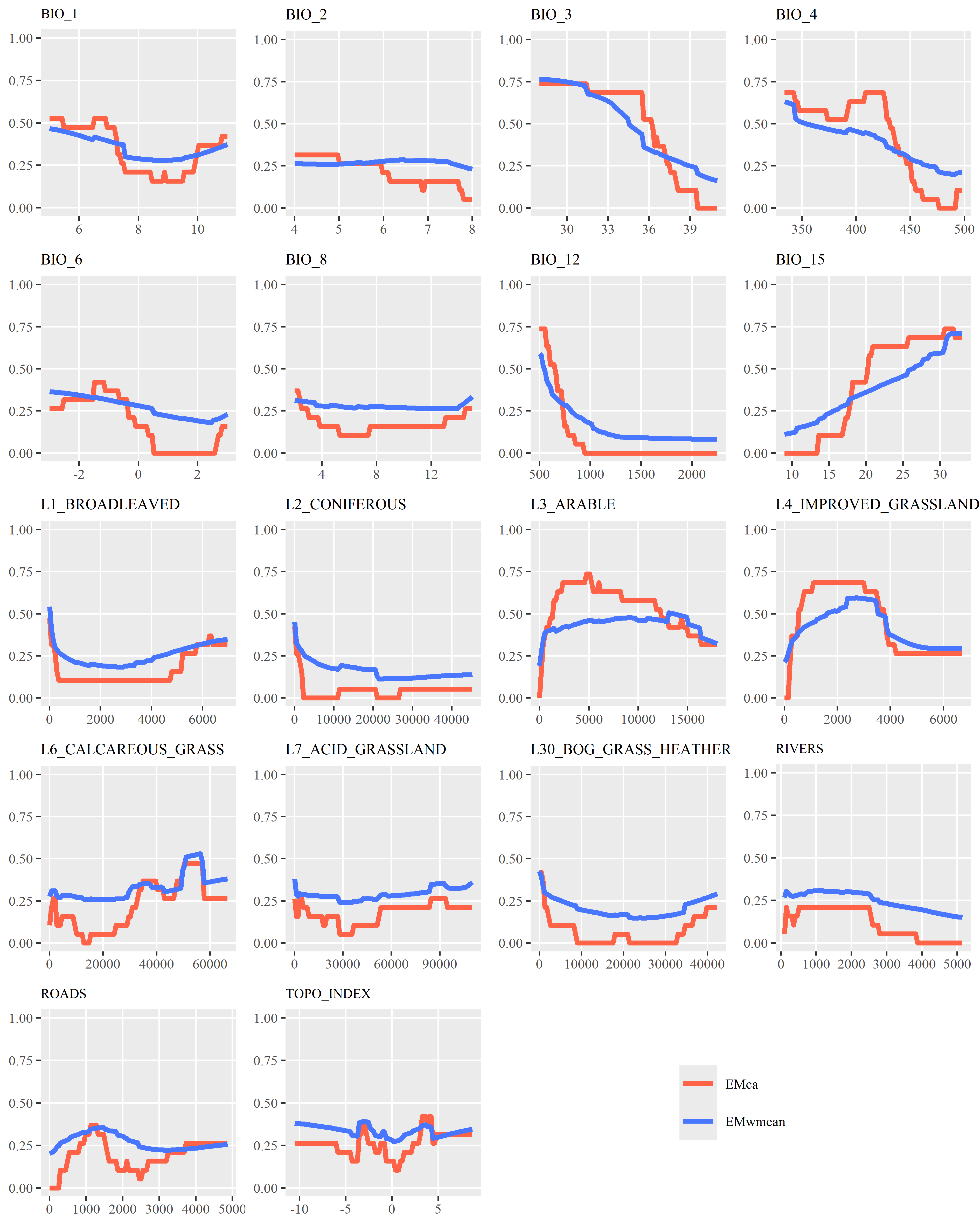

Supplement: Supplementary file 2 — Data S1: ece371956‐sup‐0002‐Supinfo.zip. [file ECE3-15-e71956-s001.zip › SUPPORTING.INFORMATION/SDM.VARIABLE.RESPONSE.PLOTS/ADDER.tif]

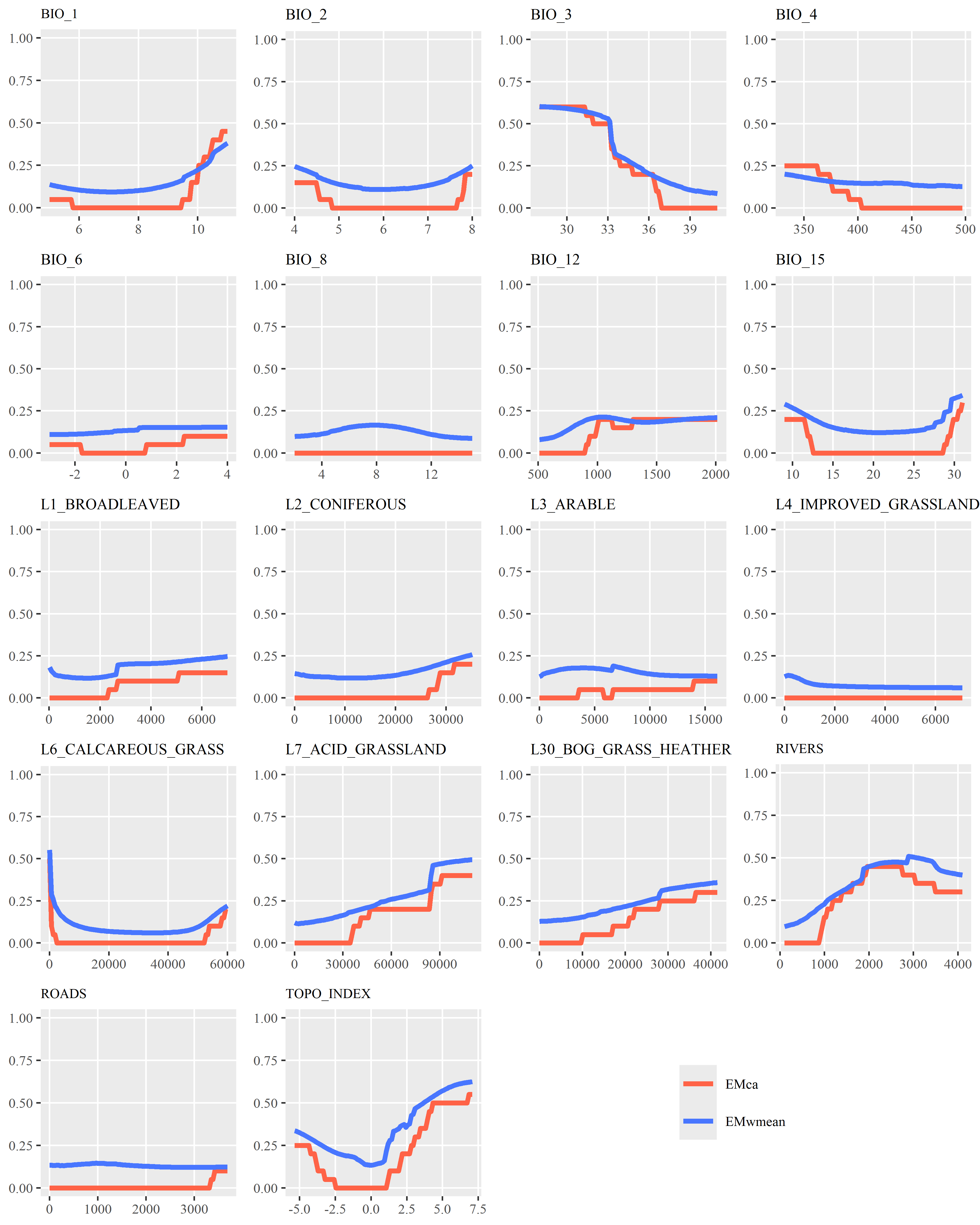

Supplement: Supplementary file 2 — Data S1: ece371956‐sup‐0002‐Supinfo.zip. [file ECE3-15-e71956-s001.zip › SUPPORTING.INFORMATION/SDM.VARIABLE.RESPONSE.PLOTS/CHALK.CARPET.tif]

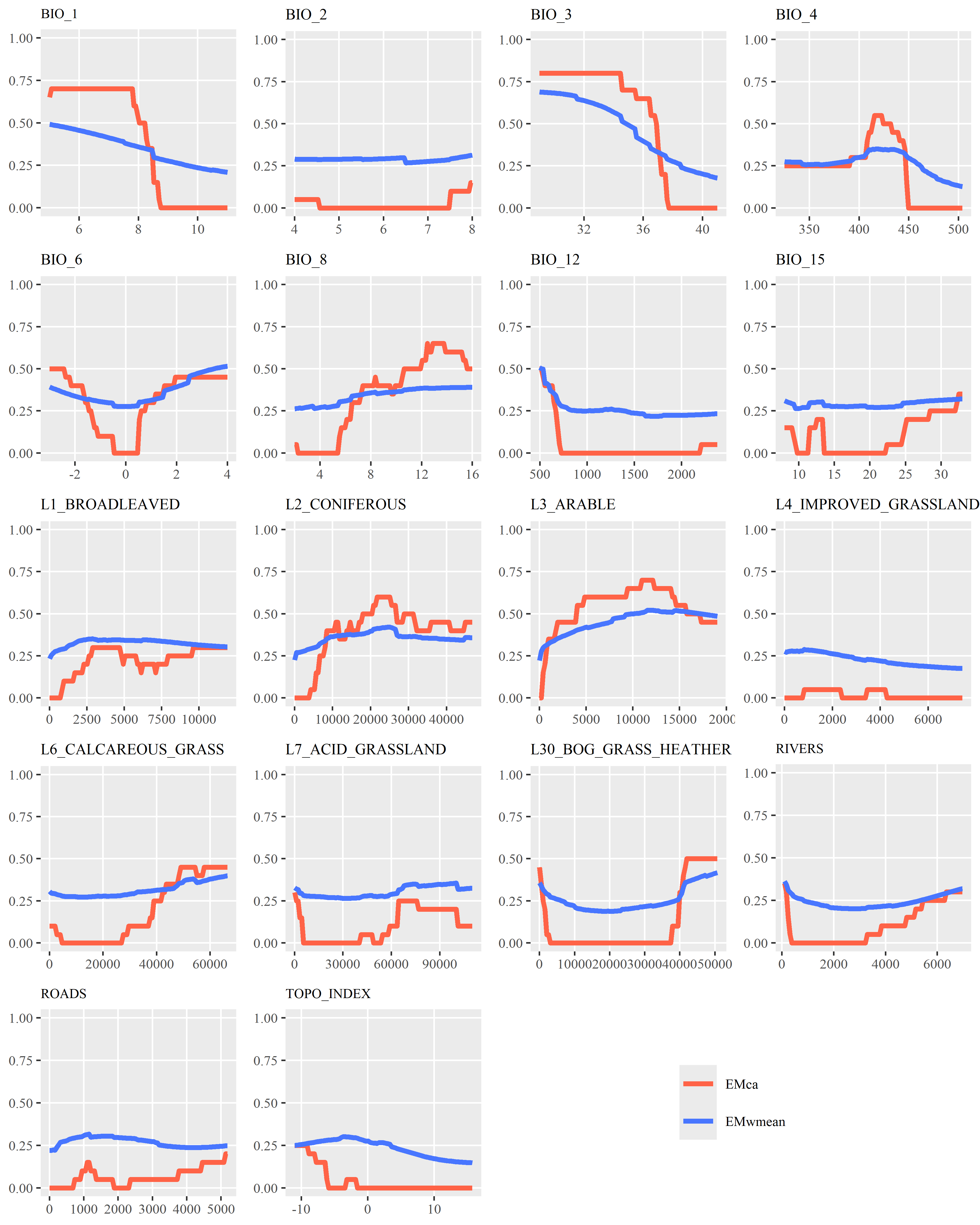

Supplement: Supplementary file 2 — Data S1: ece371956‐sup‐0002‐Supinfo.zip. [file ECE3-15-e71956-s001.zip › SUPPORTING.INFORMATION/SDM.VARIABLE.RESPONSE.PLOTS/CURLEW.tif]

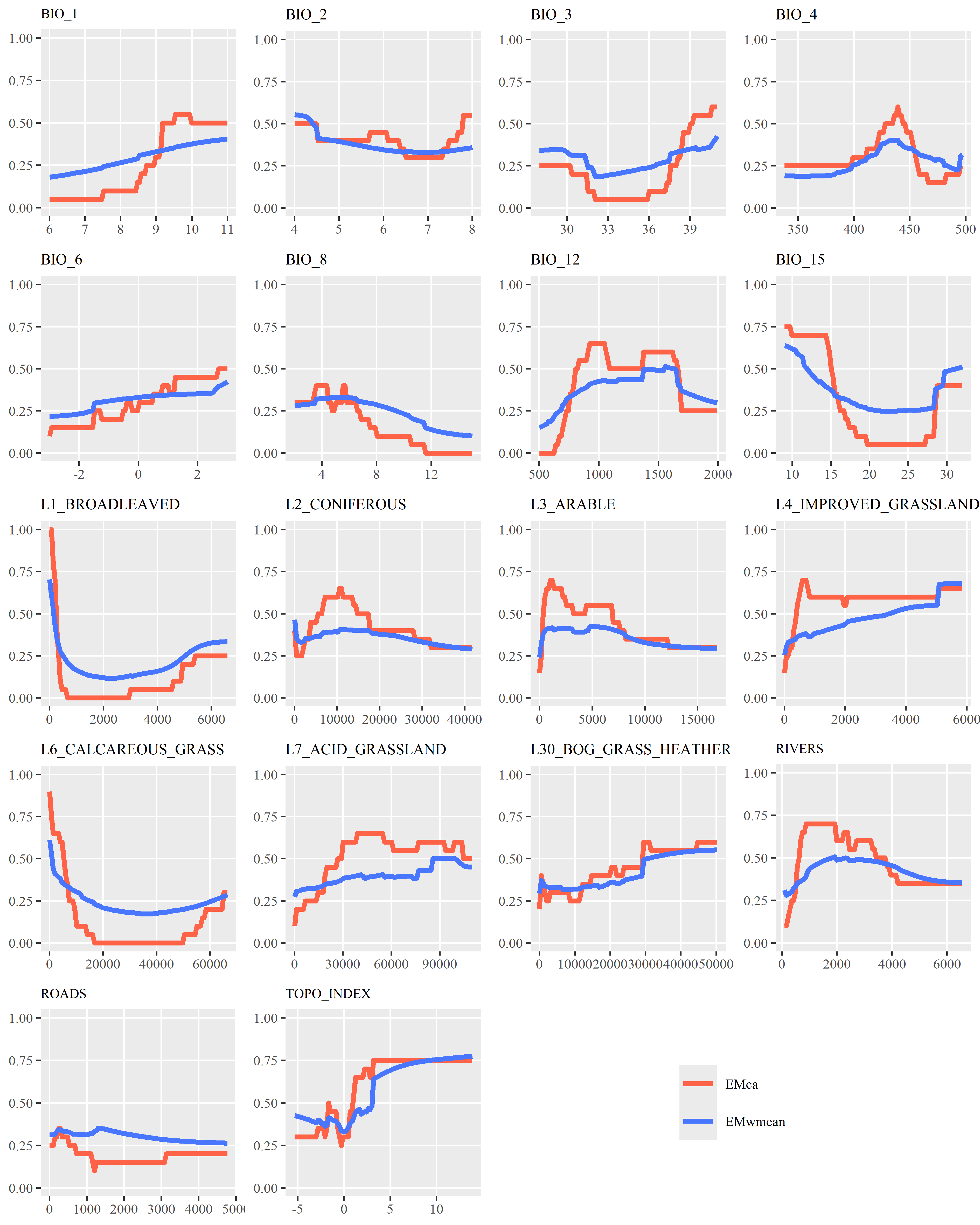

Supplement: Supplementary file 2 — Data S1: ece371956‐sup‐0002‐Supinfo.zip. [file ECE3-15-e71956-s001.zip › SUPPORTING.INFORMATION/SDM.VARIABLE.RESPONSE.PLOTS/DINGY.SKIPPER.tif]

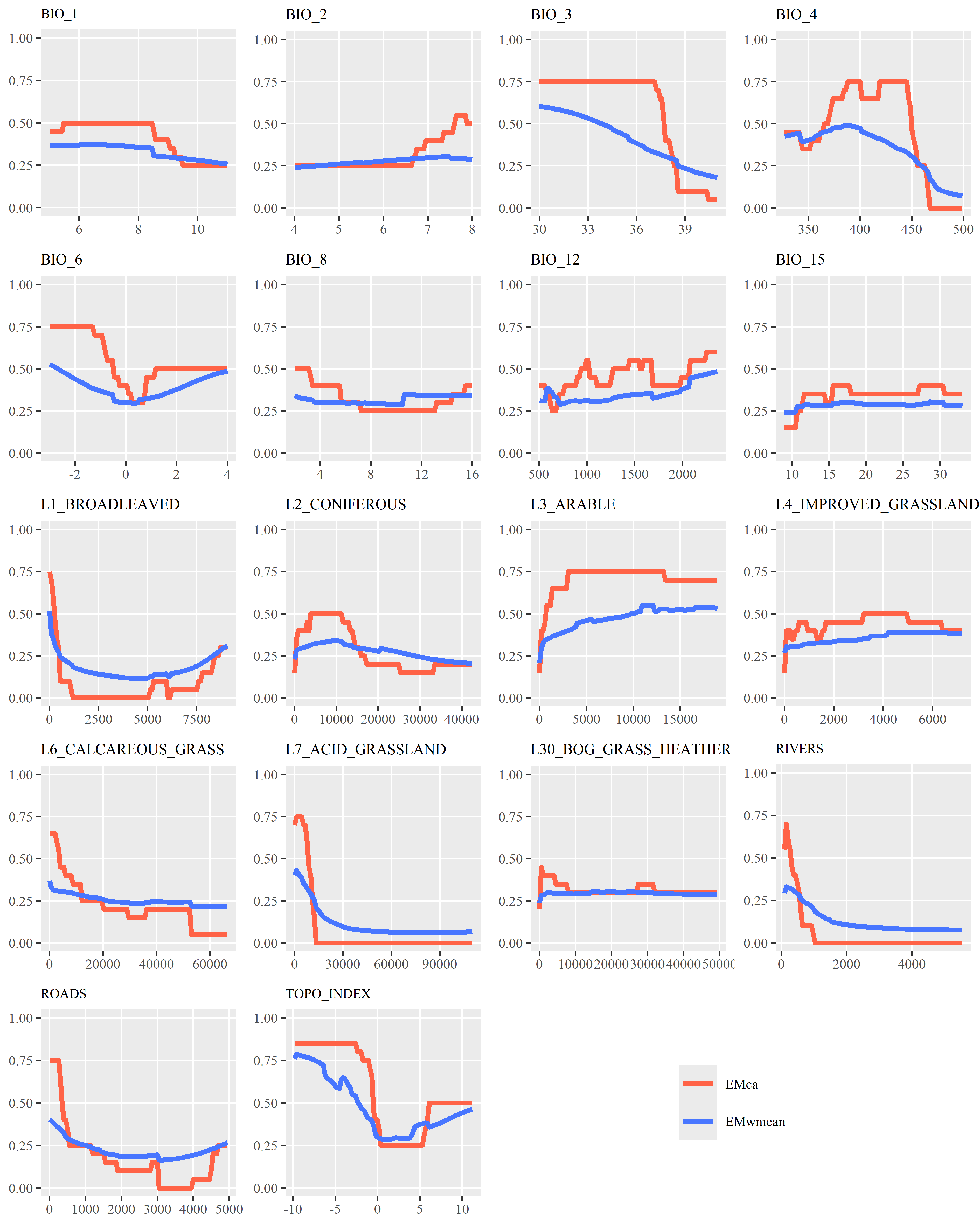

Supplement: Supplementary file 2 — Data S1: ece371956‐sup‐0002‐Supinfo.zip. [file ECE3-15-e71956-s001.zip › SUPPORTING.INFORMATION/SDM.VARIABLE.RESPONSE.PLOTS/DIPPER.tif]

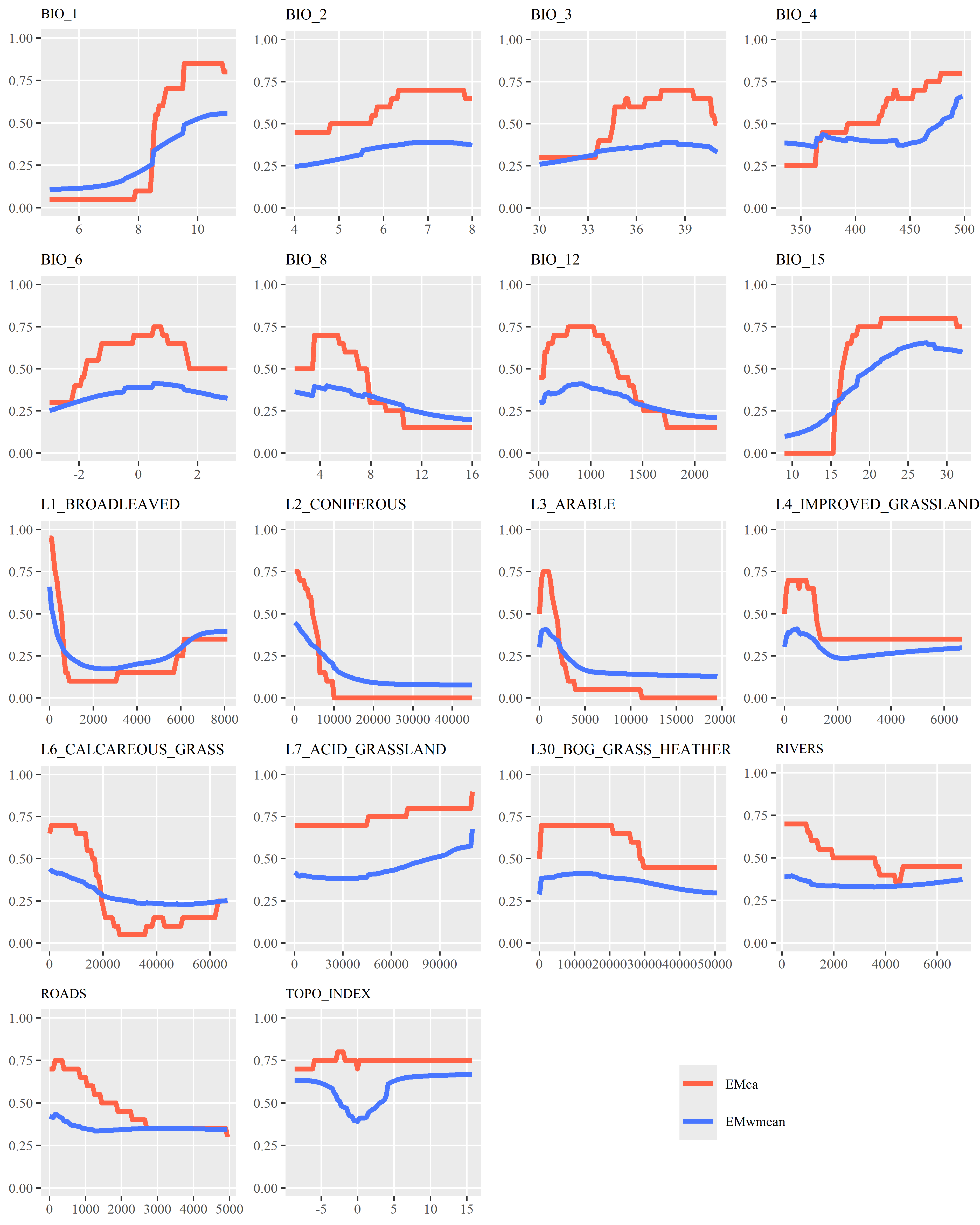

Supplement: Supplementary file 2 — Data S1: ece371956‐sup‐0002‐Supinfo.zip. [file ECE3-15-e71956-s001.zip › SUPPORTING.INFORMATION/SDM.VARIABLE.RESPONSE.PLOTS/HAZEL.DORMOUSE.tif]

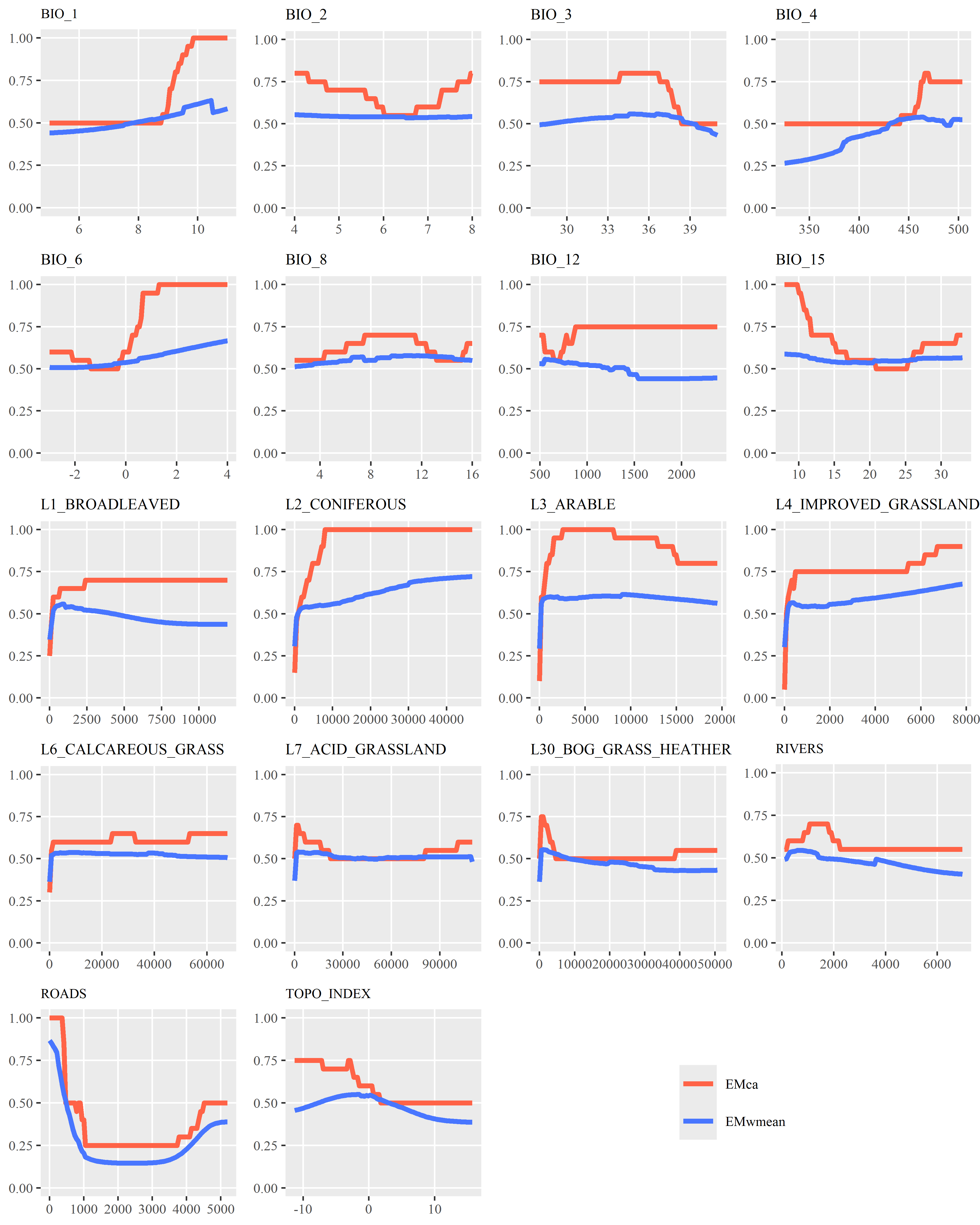

Supplement: Supplementary file 2 — Data S1: ece371956‐sup‐0002‐Supinfo.zip. [file ECE3-15-e71956-s001.zip › SUPPORTING.INFORMATION/SDM.VARIABLE.RESPONSE.PLOTS/HEDGEHOG.tif]

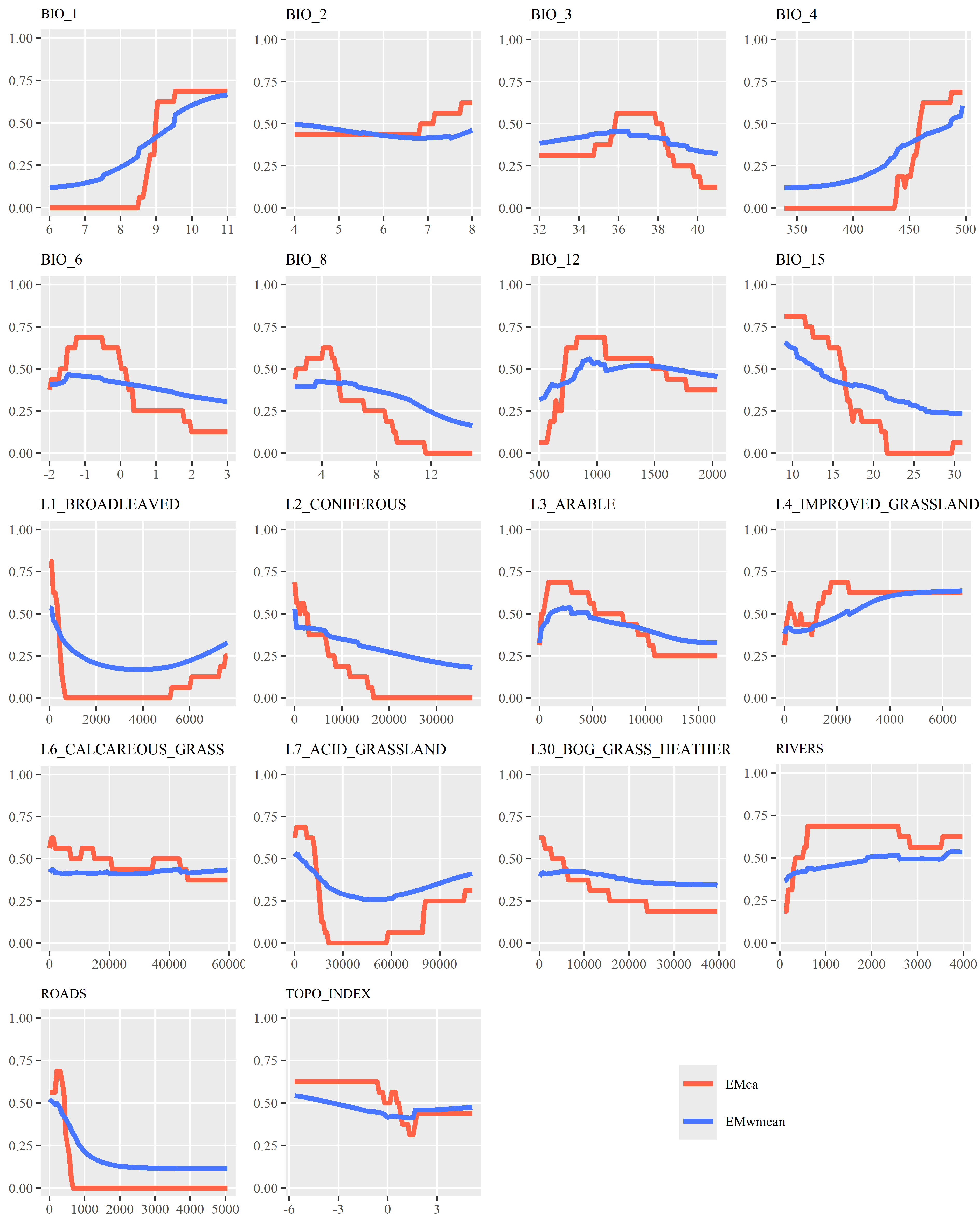

Supplement: Supplementary file 2 — Data S1: ece371956‐sup‐0002‐Supinfo.zip. [file ECE3-15-e71956-s001.zip › SUPPORTING.INFORMATION/SDM.VARIABLE.RESPONSE.PLOTS/LEISLERS.BAT.tif]

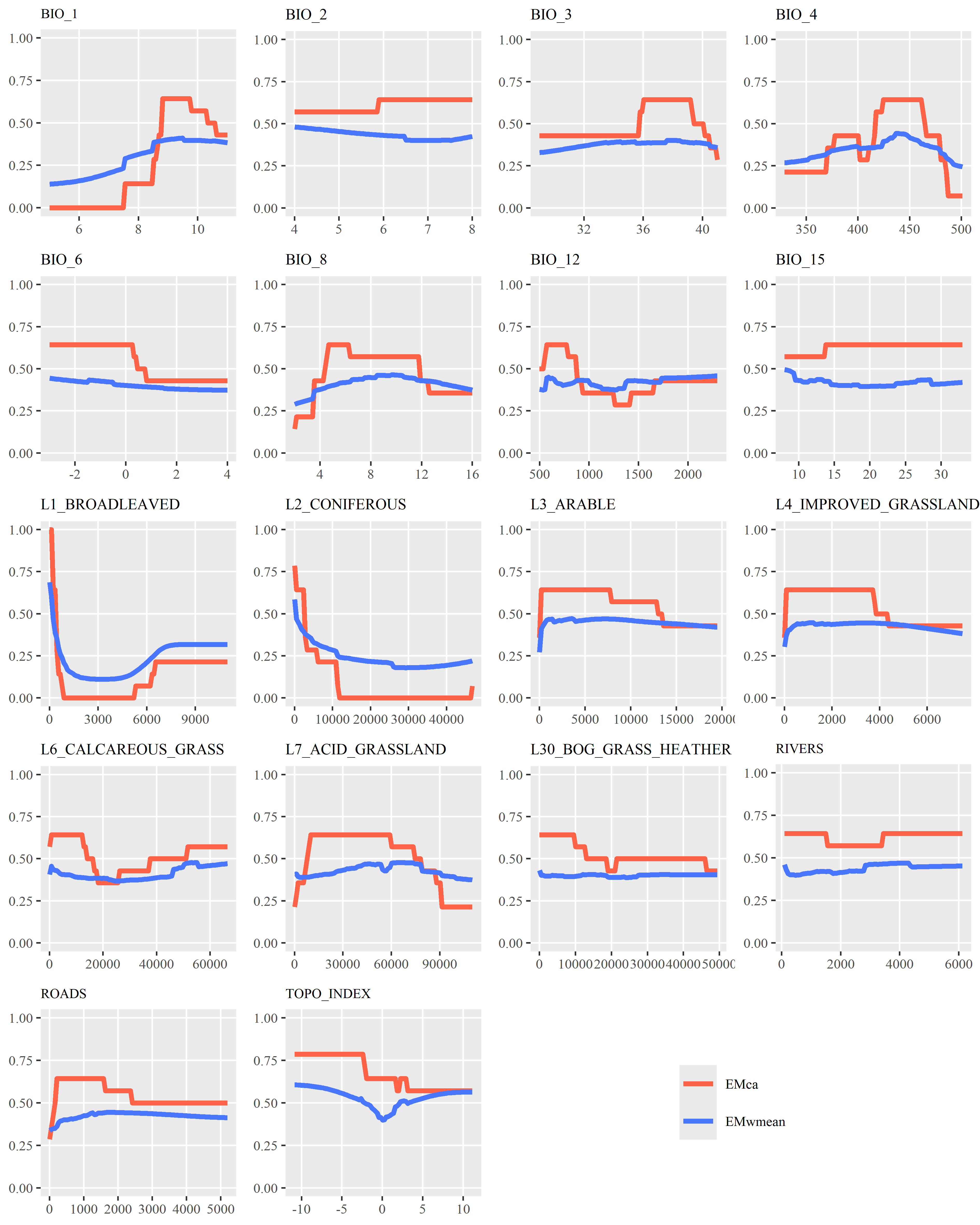

Supplement: Supplementary file 2 — Data S1: ece371956‐sup‐0002‐Supinfo.zip. [file ECE3-15-e71956-s001.zip › SUPPORTING.INFORMATION/SDM.VARIABLE.RESPONSE.PLOTS/MARSH.TIT.tif]

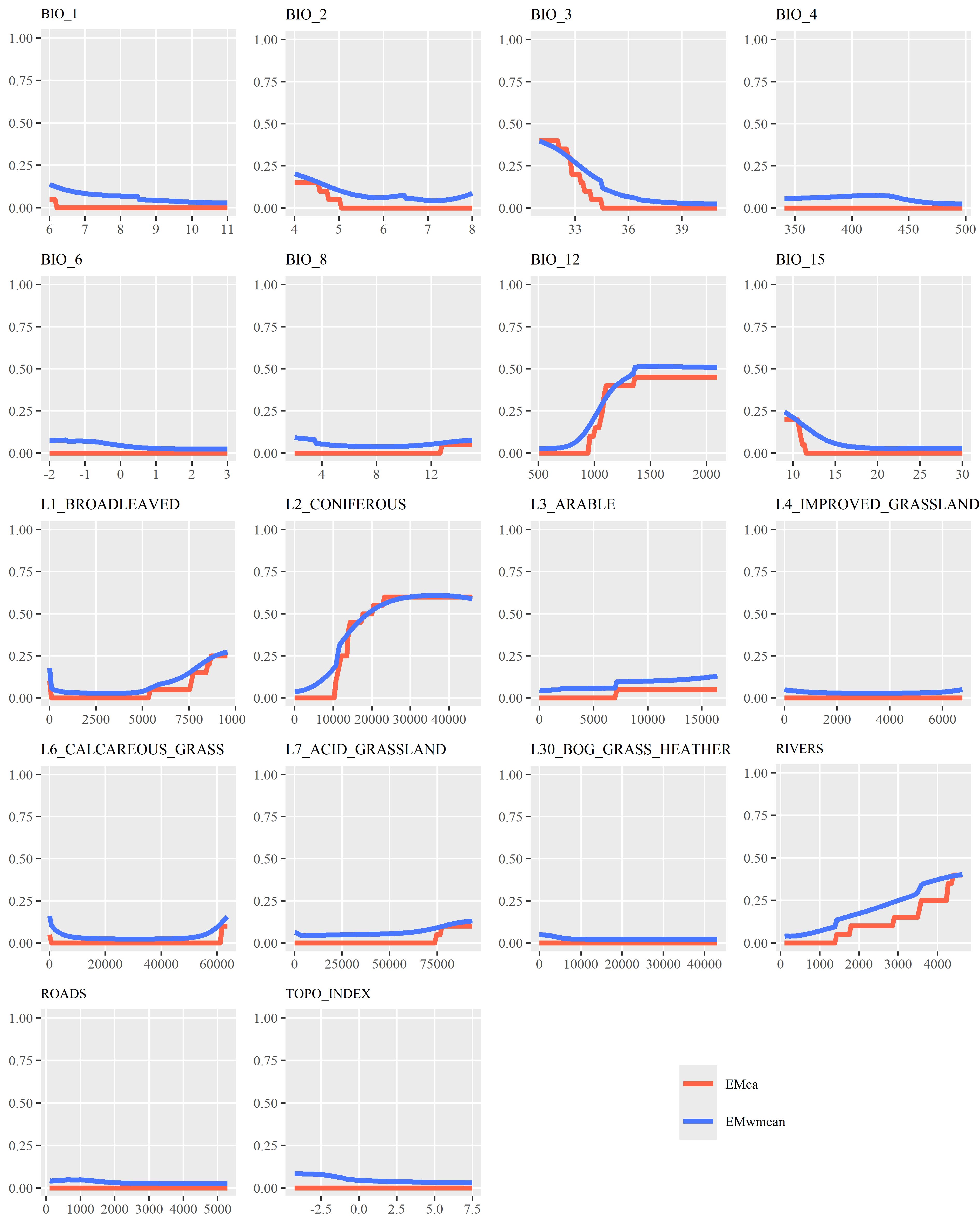

Supplement: Supplementary file 2 — Data S1: ece371956‐sup‐0002‐Supinfo.zip. [file ECE3-15-e71956-s001.zip › SUPPORTING.INFORMATION/SDM.VARIABLE.RESPONSE.PLOTS/NORTHERN.BROWN.ARGUS.tif]

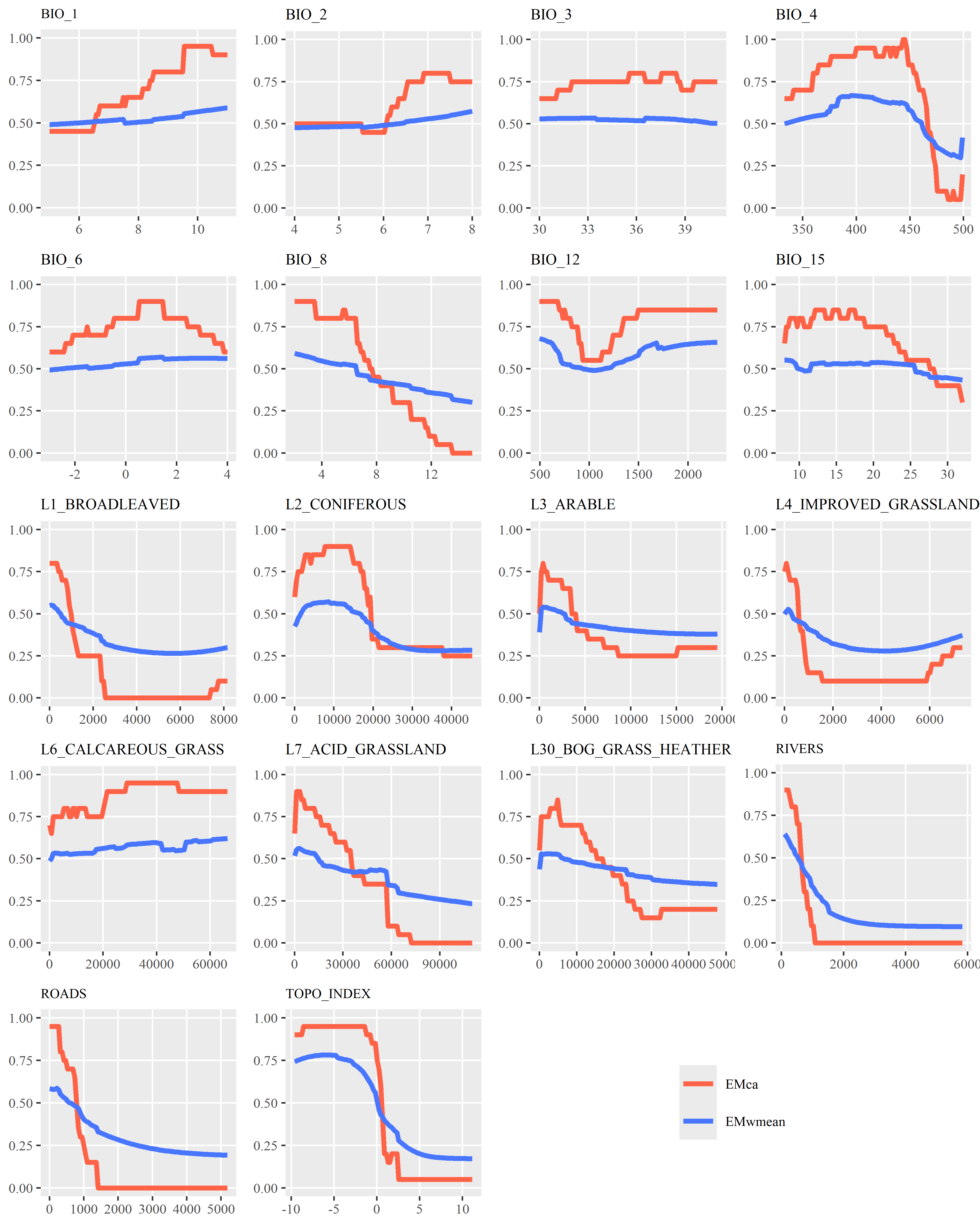

Supplement: Supplementary file 2 — Data S1: ece371956‐sup‐0002‐Supinfo.zip. [file ECE3-15-e71956-s001.zip › SUPPORTING.INFORMATION/SDM.VARIABLE.RESPONSE.PLOTS/OTTER.tif]

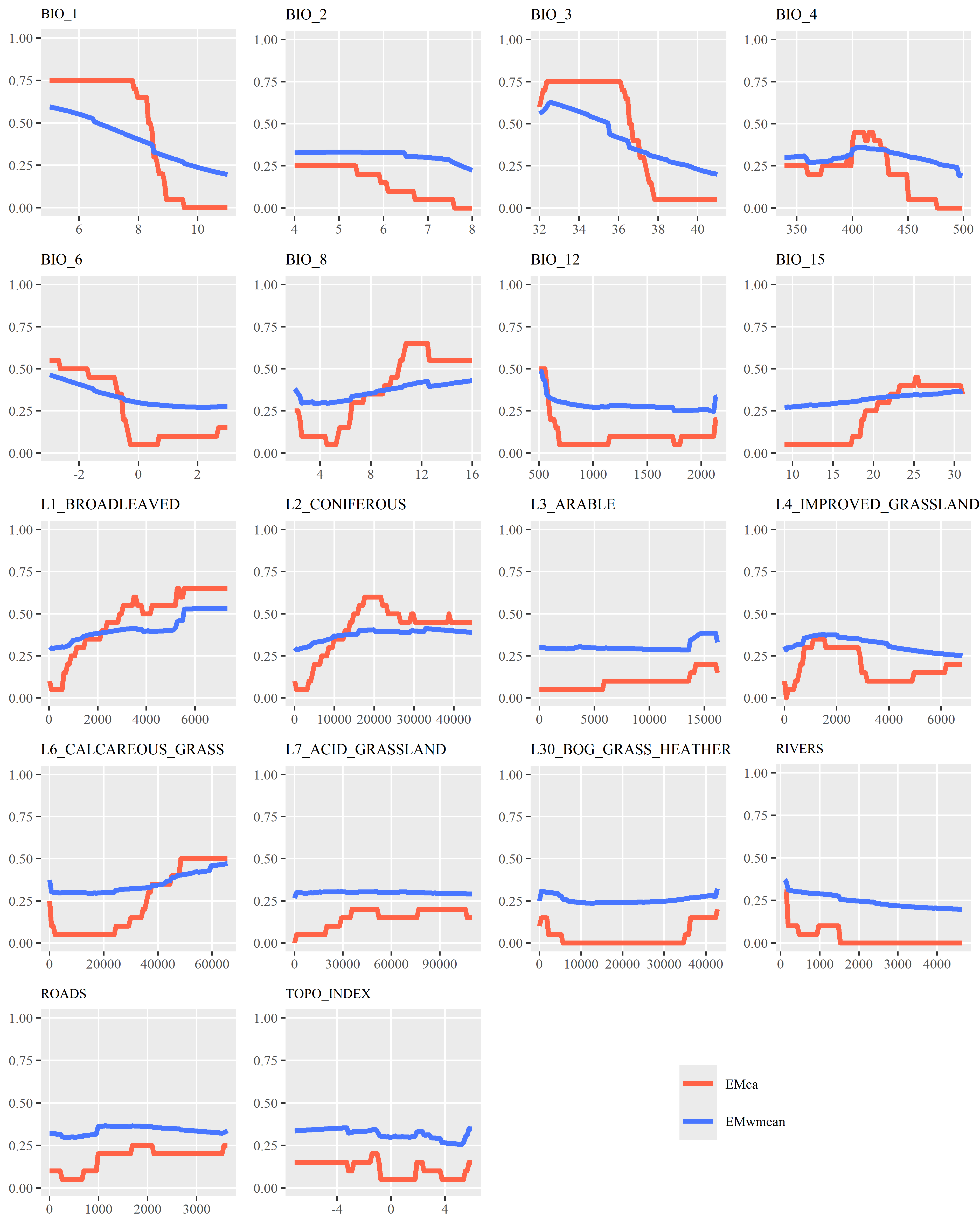

Supplement: Supplementary file 2 — Data S1: ece371956‐sup‐0002‐Supinfo.zip. [file ECE3-15-e71956-s001.zip › SUPPORTING.INFORMATION/SDM.VARIABLE.RESPONSE.PLOTS/TWITE.tif]

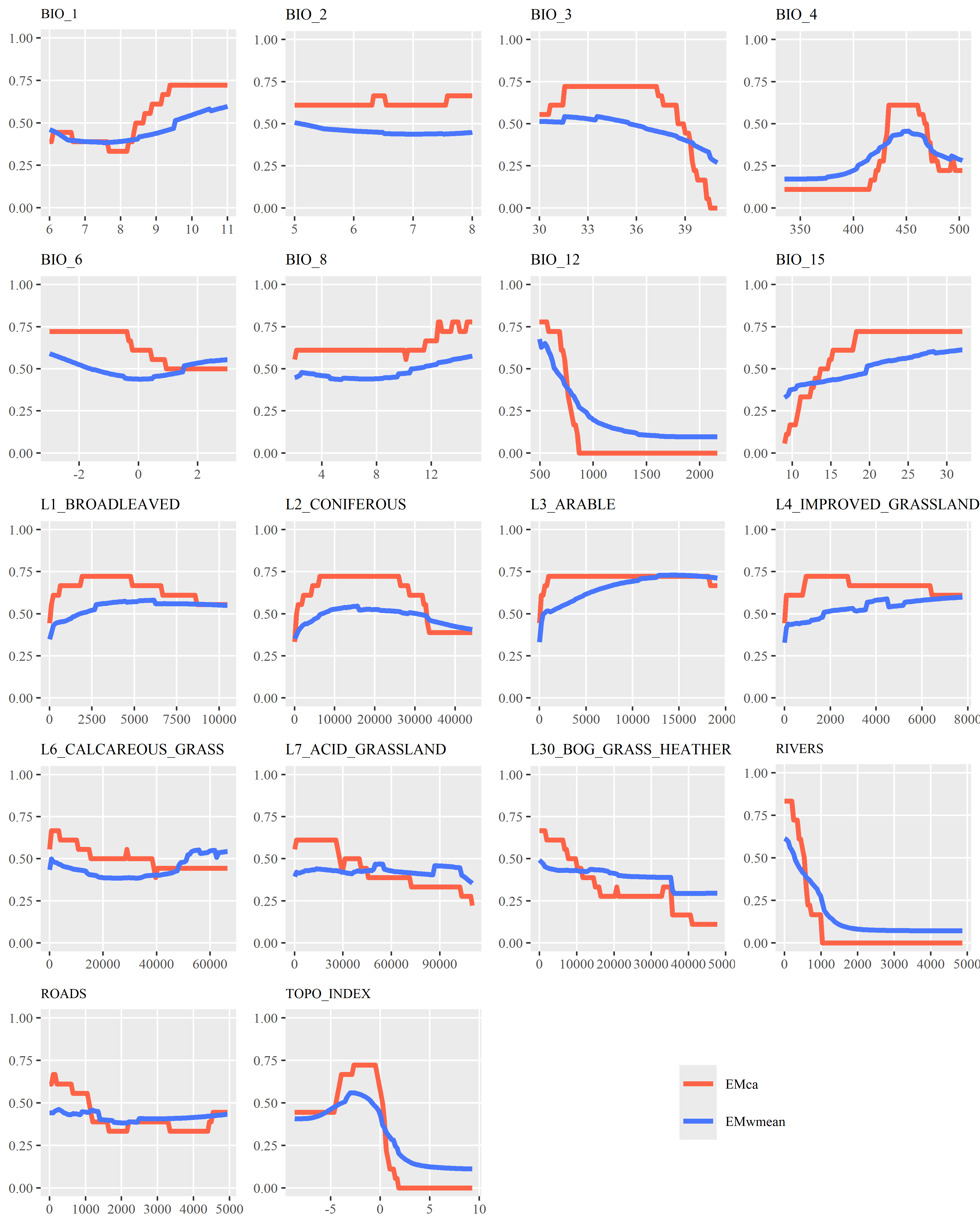

Supplement: Supplementary file 2 — Data S1: ece371956‐sup‐0002‐Supinfo.zip. [file ECE3-15-e71956-s001.zip › SUPPORTING.INFORMATION/SDM.VARIABLE.RESPONSE.PLOTS/WATER.VOLE.tif]

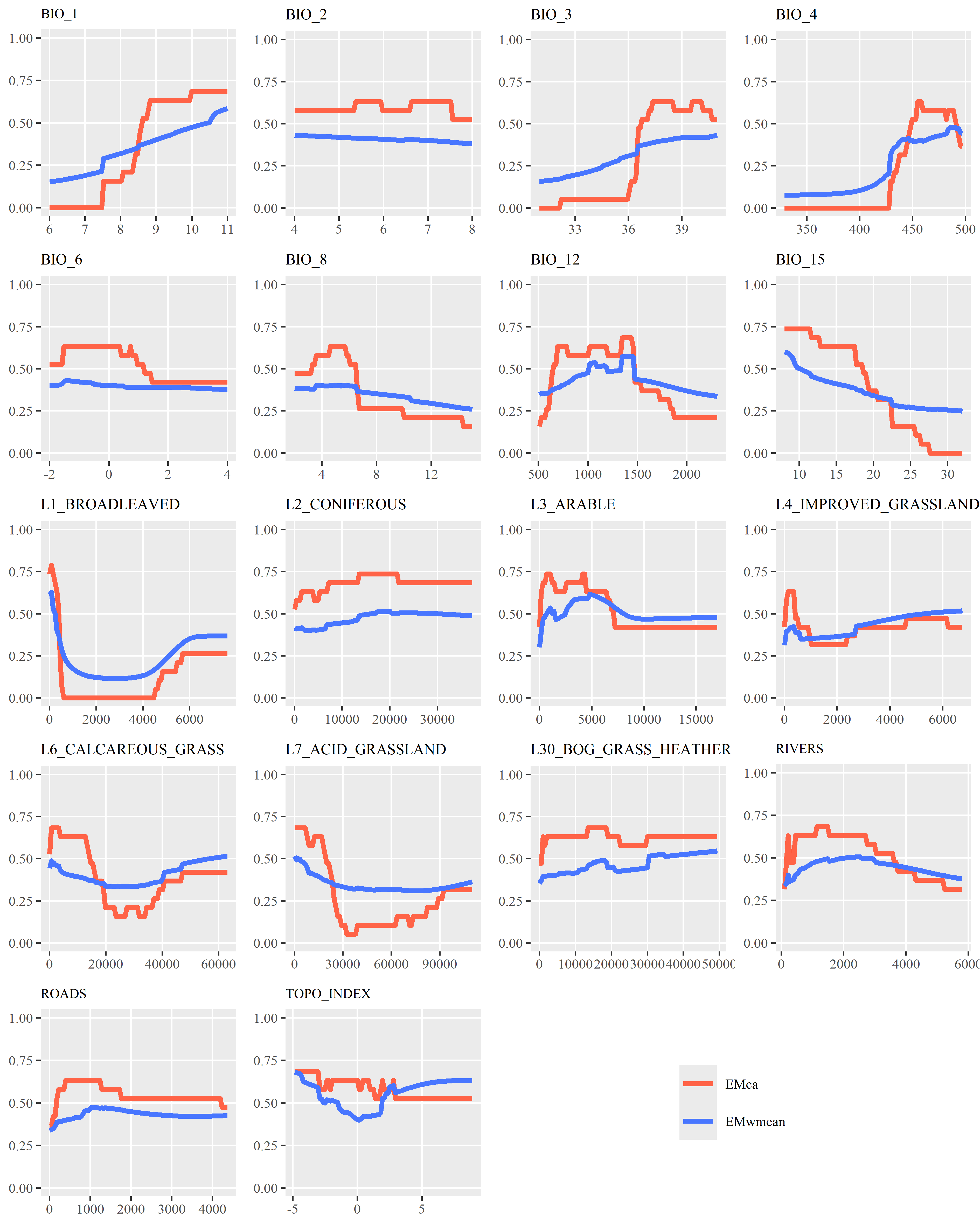

Supplement: Supplementary file 2 — Data S1: ece371956‐sup‐0002‐Supinfo.zip. [file ECE3-15-e71956-s001.zip › SUPPORTING.INFORMATION/SDM.VARIABLE.RESPONSE.PLOTS/WHITE.LETTER.HAIRSTREAK.tif]

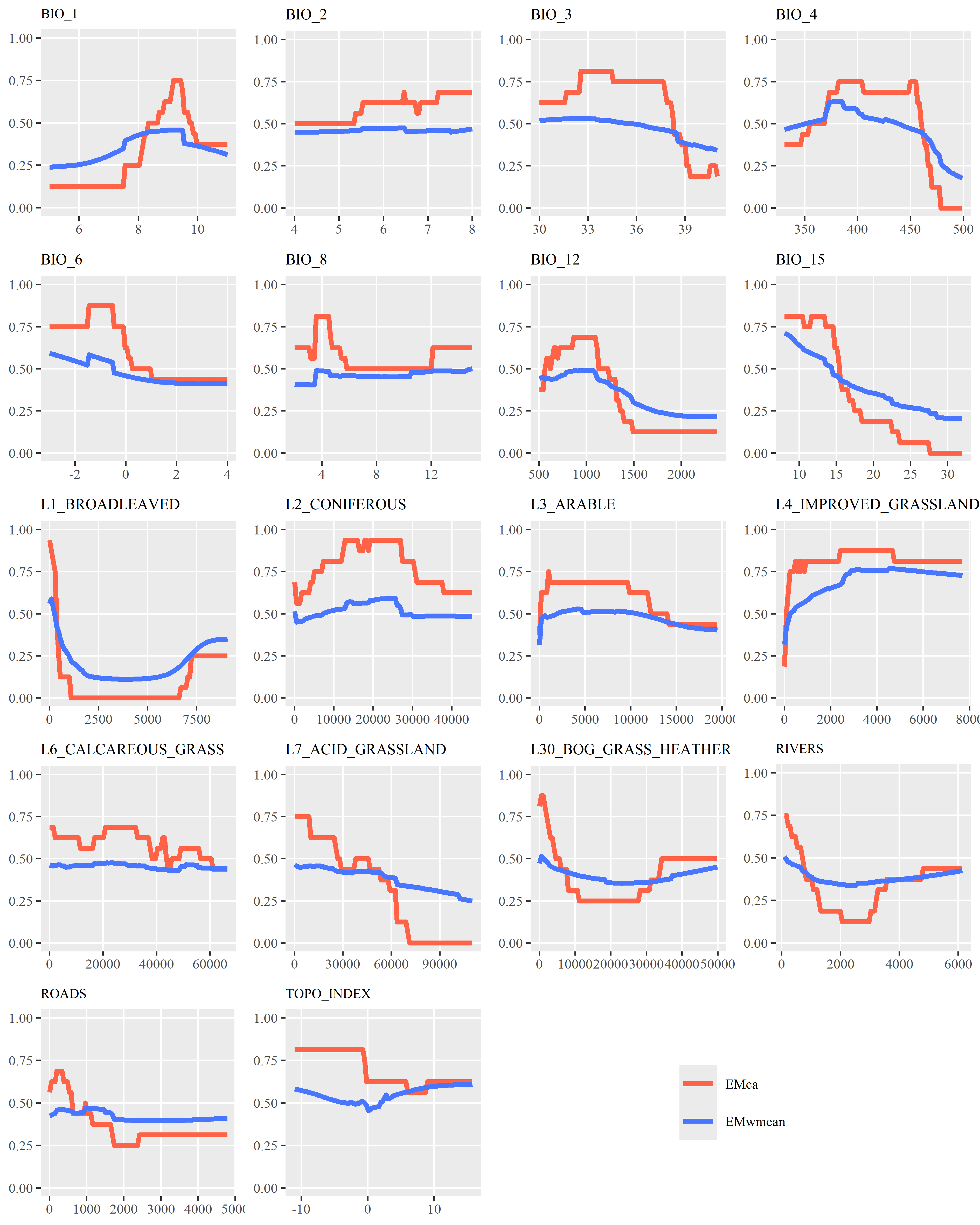

Supplement: Supplementary file 2 — Data S1: ece371956‐sup‐0002‐Supinfo.zip. [file ECE3-15-e71956-s001.zip › SUPPORTING.INFORMATION/SDM.VARIABLE.RESPONSE.PLOTS/WILLOW.TIT.tif]
